# Systemic and local chronic inflammation and hormone disposition promote a tumor-permissive environment for breast cancer in older women

**DOI:** 10.1038/s43587-026-01173-4

**Published:** 2026-07-27

**Authors:** Neil Carleton, Alexander Chih-Chieh Chang, Sanghoon Lee, Ruxuan Li, Jian Zou, Daniel D. Brown, Jagmohan Hooda, Jian Chen, Rahul Kumar, Linda R. Klei, Lora H. Rigatti, Joseph Newsome, Dixcy Jaba Sheeba John Mary, Jennifer M. Atkinson, Raymond E. West, Thomas D. Nolin, Patrick J. Oberly, Ziyu Huang, Donald Poirier, Emilia J. Diego, Peter C. Lucas, George C. Tseng, Michael T. Lotze, Priscilla F. McAuliffe, Ioannis K. Zervantonakis, Steffi Oesterreich, Adrian V. Lee

**Affiliations:** 1 Women’s Cancer Research Center, UPMC Hillman Cancer Center, Pittsburgh, PA USA; 2 Department of Bioengineering, University of Pittsburgh School of Medicine, Pittsburgh, PA USA; 3 Department of Statistics, School of Public Health, Chongqing Medical University, Chongqing, China; 4 Department of Biostatistics, University of Pittsburgh School of Public Health, Pittsburgh, PA USA; 5 Institute for Precision Medicine, University of Pittsburgh Medical Center, Pittsburgh, PA USA; 6 Division of Laboratory Animal Resources, University of Pittsburgh School of Medicine, Pittsburgh, PA USA; 7 Department of Pathology, University of Pittsburgh School of Medicine, Pittsburgh, PA USA; 8 Small Molecule Biomarker Core, University of Pittsburgh School of Pharmacy, Pittsburgh, PA USA; 9 UPMC Hillman Cancer Center Biostatistics Facility, Pittsburgh, PA USA; 10 Laboratory of Medicinal Chemistry, CHU de Québec, Research Centre Université Laval, Quebec City, Quebec Canada; 11 Division of Breast Surgical Oncology, Department of Surgery, University of Pittsburgh School of Medicine, Pittsburgh, PA USA; 12 Department of Laboratory Medicine and Pathology, Mayo Clinic College of Medicine and Science, Rochester, MN USA; 13 Department of Immunology, University of Pittsburgh School of Medicine, Pittsburgh, PA USA; 14 Department of Pharmacology and Chemical Biology, University of Pittsburgh School of Medicine, Pittsburgh, PA USA

**Keywords:** Experimental models of disease, Cancer, Ageing

## Abstract

Estrogen receptor-positive (ER^+^) breast cancer is the most common subtype of breast cancer and is an age-related disease. How systemic and tumor microenvironment hormone signaling intersects with chronic inflammation and how this shapes aging-associated tumor biology remain incompletely understood. Here, using an aged rat model of ER^+^ tumors, we identify age-associated differences in tumor development and immune features. In parallel, analysis of samples from patients with ER^+^/HER2^−^ breast cancer and age-matched controls demonstrated estrone-predominant systemic estrogen patterns and increased tumor HSD17B7 expression in older patients, consistent with enhanced local estrone-to-estradiol conversion. In patient-derived organoids from older women, pharmacologic inhibition of HSD17B7 reduced estrogen conversion and proliferation-associated transcriptional programs. Tumors from older patients also exhibited chemokine enrichment, including CCL2, associated with immunosuppressive macrophage features. Targeting both estrogen signaling and chemokine pathways attenuated macrophage polarization ex vivo. These findings support a model linking age-associated hormonal and inflammatory changes to features of a tumor-permissive microenvironment in ER^+^ breast cancer.

## Abstract (web summary)

Using an aged rat model, patient tissue samples and patient-derived organoids, Carleton et al. profiled age-associated differences in estrogen receptor-positive breast cancer, identifying inflammation and altered estrogen metabolism as possible contributors to age-related tumor permissiveness.

## Main

Breast cancer, including the most common estrogen receptor-positive (ER^+^) subtype, is an age-dependent disease^1–3^. Despite reduced circulating estradiol (E2) levels postmenopause, the proportion of ER^+^ tumors rises with advancing age^4^.

Although accumulating mutations are important contributors to the age-related nature of breast cancer^5,6^, the extent to which the local and systemic environment influences tumor development and growth is less well known. Previous work minimizes these genomic changes in favor of the hypothesis that transcriptomic differences underlie the major difference in age-related breast tumors^5,7^. Tumors from older patients with ER^+^ disease are far less likely to exhibit gene expression changes indicative of faster-growing tumors, such as growth factor receptor activation and p53 enrichment^7,8^. In a large study that included both breast cancer and normal breast tissues, the predominant age-correlated gene expression changes in breast cancers were attributable to changes in physiologic estrogen signaling, indicating a critical but unexplored role of how circulating and local factors, such as estrogen disposition and chronic inflammation, affect breast cancer development and growth in older women^9^.

Aging is accompanied by a persistent, low-grade inflammatory state often referred to as ‘chronic inflammation’ or ‘inflammaging’. This has been implicated in the development of multiple age-related diseases, including cancer^10,11^. Although previous work has emphasized canonical cytokines such as IL-1β, IL-6 and IL-8 as markers of age-associated inflammation, emerging evidence suggests that aging may also be characterized by sustained elevations in chemokines that shape immune cell recruitment, tissue composition and immune function rather than induce acute inflammatory signaling^12^. Despite strong epidemiologic links between chronic inflammation and cancer risk, how specific inflammatory programs in the aging host translate into functional changes within the local tumor microenvironment (TME) remains poorly defined.

Despite the high prevalence of breast cancer in older patients, they remain underrepresented in clinical and genomic studies, limiting age-informed biological insight^13,14^. Here we examine how age-associated systemic changes relate to alterations in the local breast TME, with a focus on hormone signaling and inflammation. Integrating aging models and human datasets, we identify age-related differences in estrogen disposition and inflammatory profiles that are associated with features of an immunologically altered TME.

## Results

### Aged environment promotes tumors in older F344 rats in the DMBA/MPA carcinogen model

**Fig. 1:**
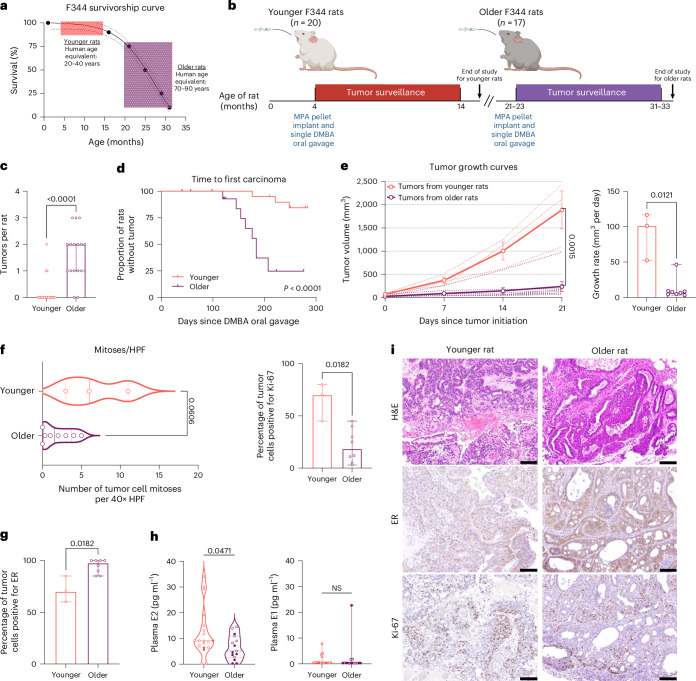
The aged environment promotes accelerated yet indolent tumorigenesis in a rat model of postmenopausal ER^+^ breast cancer. **a**, Survivorship curve showing the human equivalent age for the younger and older rats used in the study, which is based on general human population survivorship curves and F344 rat survivorship curves from the National Institute on Aging. **b**, Schematic of the experimental setup. In brief, younger F344 rats (*n* = 20, ~4 months old) and older F344 rats (*n* = 17, ~21–23 months old) were implanted with a medroxyprogesterone acetate (MPA) pellet, treated with a single dose of the 7,12-dimethylbenz[*a*]anthracene (DMBA) carcinogen and subsequently monitored for tumor development over a 10-month time period. **c**, Older rats harbored significantly more tumors than younger rats. This plot includes all tumors that formed, including both fibroepithelial lesions and mammary carcinomas. Data represent median and 95% CI. **d**, Time-to-event analysis showing the time to first carcinoma. Older rats had a significantly shorter time to first carcinoma compared with younger rats (log-rank *P* < 0.0001). Overall, there were three carcinomas in the younger rats and eight carcinomas in the older rats. **e**, Tumors in older rats generally grew more slowly, as evidenced by both the tumor growth curves (all tumors compared at day 21; solid lines represent the average growth for each age group, whereas the dotted lines show individual tumor trajectories) and the tumor growth rate (computed using the total tumor volume at the time of euthanasia). Data represent median and 95% CI. **f**, Mitoses per high-power field (HPF) and the percentage of tumor cells staining positive for Ki-67; both data computed by a trained animal histopathologist using at least four different HPFs per tumor. The final average of all HPFs examined from each tumor is plotted. All carcinomas were included in the analysis (*n* = 3 younger rats; *n* = 8 older rats). Data show median and range for mitoses per HPF plot and median and 95% CI for Ki-67 plot. **g**, Percentage of tumor cells staining positive for ER. Data show median and 95% CI. All carcinomas were included in the analysis (*n* = 3 younger rats; *n* = 8 older rats). **h**, Plasma E2 and plasma E1 analyzed on isolated serum at the time of euthanasia for all younger (*n* = 20) and older rats (*n* = 17) in the study. Filled-in dots indicate rats that had carcinomas at the time of euthanasia. Data show median with range. **i**, Representative tumor images (H&E, ER staining and Ki-67 staining) for younger and older rats. Scale bars, 100 μm. Two-tailed Mann–Whitney test was performed for all pairwise comparisons; plots show median with 95% CI. NS, not significant. Schematic in **b** created in BioRender; Carleton, N. https://biorender.com/7qnorrj (2026). Source data

**Extended Data Fig. 1:**
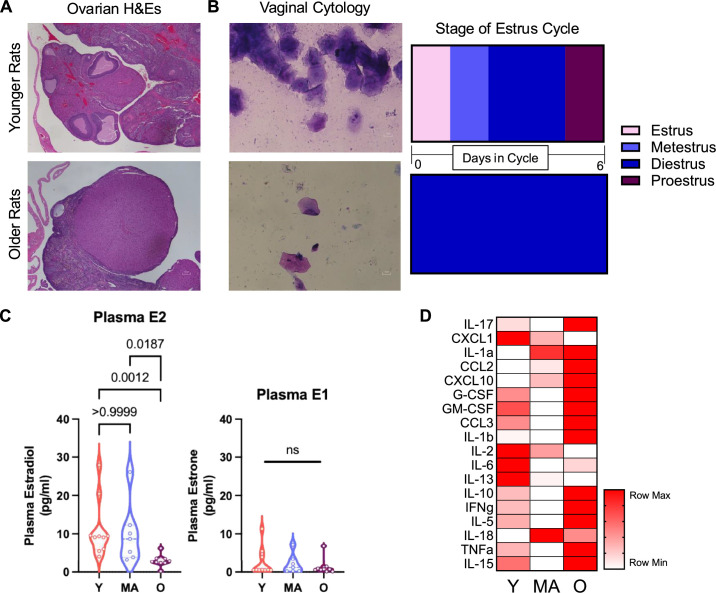
Assessment of physiological aging and estropause in F344 rats that were not exposed to chemical carcinogens. (**A**) Ovarian H&E-stained slides from younger and older rats. Ovaries from younger rats ( ~ 4mo old) showed active follicle formation and cycling while the ovaries from older rats (20-22mo old) showed afollicular ovaries. Representative images shown with similar results for all rats in the cohort (n = 6 younger, n = 6 older). (**B**) Representative images from vaginal cytology, in which n = 6 younger and n = 6 older rats were swabbed for 14 consecutive days. Younger rats were actively cycling every 5-6 days while the older rats were in a constant state of diestrus. (**C**) Analysis of circulating estradiol (E2) and estrone (E1) in young ( ~ 4mo old), middle-aged (12-13mo old), and older (20-22mo old) showed a significant decline in circulating E2 in the older rats, consistent with the human phenotype of menopause. ANOVA with correction for multiple comparisons used for statistical testing. (**D**) Circulating inflammation panel on the same groups of rats showed age-related chronic inflammatory phenotype in older rats. Source Data

The Fischer 344 (F344) inbred rats serve as one of the most commonly used models for aging, cancer and toxicology studies. Despite this, relatively little has been reported on their estrogen levels, menstrual cycles and age-related inflammation. We examined these age-related changes in estrogen disposition and inflammation in young (4 months old), middle-aged (12–13 months old) and older (20–22 months old) F344 rats not exposed to carcinogens. Four months of age corresponds to about the age of 20–30 years for humans, whereas 21–23 months of age is equivalent to about the age of 70 years (Fig. 1a). Hematoxylin and eosin (H&E)-stained sections of ovaries from young and older rats showed that the younger rats had active follicle formation, whereas the older rats had no follicles present (Extended Data Fig. 1a). Vaginal cytology for 14 consecutive days in six young and six older rats showed that young rats tended to progress through the estrus cycle every 5–6 days, whereas the older rats, with prominent and persistent pattern of cornified epithelial cells, were in a state of diestrus (Extended Data Fig. 1b). This was concordant with the measured serum E2 and estrone (E1) levels, which showed that older rats had significantly lower levels of E2 in a post-estropause state (Extended Data Fig. 1c). Measurements of circulating inflammatory factors using an 18-plex assay revealed an inflammatory state in the older rats characterized by increases in several chemokines and TNF-α, IL-1β and IL-10 (Extended Data Fig. 1d).

This characterization of estrogen levels, menstrual cycles and age-related inflammation in F344 rats provided insights into their systemic environment. Next, to determine whether and to what degree the aged environment affects tumor development and growth, we used a carcinogen-induced model of ER^+^ breast cancer, which included a single oral gavage of the carcinogen DMBA and an implanted slow-release pellet of MPA. This model has been previously shown to produce estrogen-sensitive tumors that rely on immune escape for tumor development and growth and generally have a prominent inflammatory reaction within the TME^15–17^.

Younger rats (*n* = 20) were treated with the carcinogen at 4 months of age, whereas older rats (*n* = 17) were treated at 21–23 months of age (Fig. 1b). Following treatment, older rats developed significantly more tumors, including both carcinomas and fibroepithelial lesions, than younger rats (*P* < 0.0001). It is well known that aged F344 rats develop spontaneous mammary fibroepithelial lesions (a phenomenon that is associated with increasing age and thus not observed in younger rats)^18^, although our data suggest that the carcinogen accelerated the growth of both benign and cancerous lesions. The younger rat cohort only developed three tumors after carcinogen exposure. After excluding the fibroepithelial lesions, the older rats had a significantly shorter time to first carcinoma (*P* < 0.0001) (Fig. 1c,d). Notably, tumors in older rats grew significantly slower than those in younger rats (*P* = 0.0015) and accordingly had fewer mitoses per 40× field (*P* = 0.06) and lower Ki-67^+^ tumor cells (*P* = 0.02) (Fig. 1e,f). ER expression was higher in the tumors from older rats (*P* = 0.02) despite lower circulating levels of E2 (*P* = 0.04) (Fig. 1g–i). These data suggest not only that the carcinogen-induced rat model recapitulates key features of age-related, postmenopausal breast cancer in humans but also that tumorigenesis in older rats occurs in a distinct systemic and immune context compared with that in younger counterparts.

**Fig. 2:**
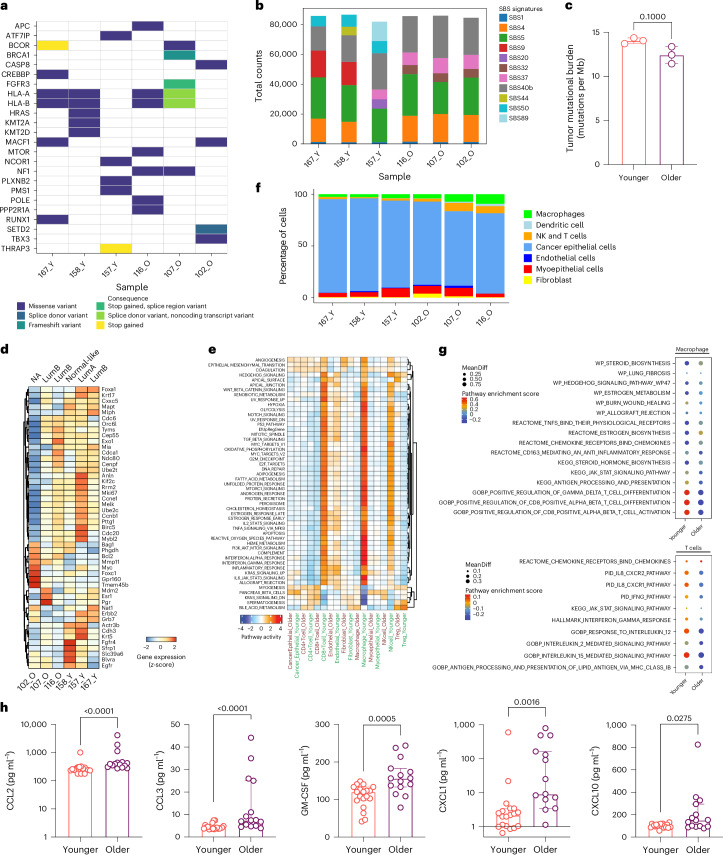
Accelerated tumorigenesis in older rats correlates with substantial inflammation and immune changes but limited differences in cancer cell-intrinsic factors. **a**, Oncoplot for the three tumors from younger rats (labeled with tumor ID and ‘Y’) and three tumors from older rats (labeled with tumor ID and ‘O’) showing random mutations induced by the carcinogens without a pattern related to age of the host. **b**, COSMIC signature analysis shows enrichment of SBS4 and SBS5 signatures across all tumors, related to the carcinogenic nature of the tumors. **c**, Tumor mutational burden was decreased in the tumors from older rats; *n* = 3 older rats and *n* = 3 younger rats. Two-tailed Mann–Whitney test was performed for pairwise comparison; plot shows median with 95% CI. **d**, PAM50 analysis shows no distinct patterns by age; tumors aligned to luminal A-like, luminal B-like and normal-like. One tumor could not be assigned a PAM50 subtype. **e**, Using the snRNA-seq data, we analyzed HALLMARK pathway expression across the immune cell subtypes in tumors from younger versus older rats. **f**, Using snRNA-seq, we analyzed proportions of cells in the TME, showing that cells from the myeloid lineage were higher in the tumors from older rats compared with the tumors from younger rats. **g**, Pathway enrichment analysis from snRNA-seq data between macrophages and T cells in the tumors of younger and older rats. **h**, Cytokine analysis on the blood taken at the time of euthanasia (through cardiac puncture) shows significantly enriched levels of circulating chemokines in the older rats; *n* = 15 older rats and *n* = 19 younger rats included in analysis. Two-tailed Mann–Whitney test was performed for all pairwise comparisons. Data show median and 95% CI. Source data

**Extended Data Fig. 2:**
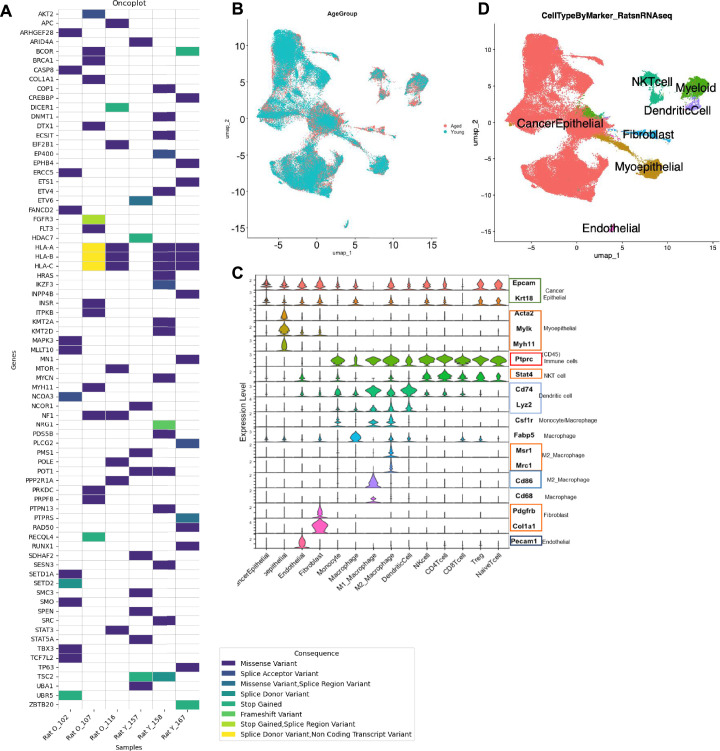
Sequencing studies of rat tumors show random mutation patterns and little genomic differences between tumors from younger and older rats. (**A**) Oncoplot for the three tumors from younger rats (labeled with tumor ID and “Y”) and three tumors from older rats (labeled with tumor ID and “O”) displaying a broader group of genes. Plot shows random mutations induced from the carcinogens without a pattern related to age of the host. (**B**) UMAP from snRNA-seq data showing overlap between cells from tumors from young and older rats. (**C**) Marker genes used to identify cell types within the TME from snRNA-seq data. (**D**) UMAP showing the identified cell types.

Next, we used whole-exome sequencing, bulk RNA sequencing (RNA-seq) and single-nucleus RNA-seq (snRNA-seq) to further characterize age-related changes. Carcinogen-induced tumors showed a random mutational pattern across age groups with no clear age-correlated differences (Extended Data Fig. 2a); when restricting to commonly mutated genes in human breast cancer, we again found minimal differences across age groups (Fig. 2a). COSMIC signature analysis revealed enrichment of SBS4 (signal of direct DNA mutagenesis) and SBS5 (clock-like signature in bladder cancer and other cancer types associated with tobacco smoking) across both age groups, likely reflecting the DMBA-induced nature of the tumors; SBS9 and SBS89, proposed to be markers of carcinogenesis in the first decade of life, were only present in tumors from young rats (Fig. 2b). Interestingly, tumor mutational burden showed a decreased trend in tumors from older rats (*P* = 0.10; Fig. 2c). PAM50 subtyping of the tumors, which showed enrichment for mostly luminal A- and luminal B-like intrinsic subtypes, did not vary by age (Fig. 2d). snRNA-seq of the tumors further confirmed minimal transcriptomic differences between cancer epithelial cells in tumors from younger and older rats (Fig. 2e and Extended Data Fig. 2b). Given the lack of genomic and transcriptomic differences in tumors from younger and older rats, we further explored immune (snRNA-seq; cell marker and identification from snRNA-seq in Extended Data Fig. 2c,d) and inflammatory (circulating inflammation panel) differences. Cells from the myeloid lineage generally increased with age (higher proportions in tumors from older rats, Fig. 2f), yet these infiltrative macrophages in tumors from older rats exhibited lower levels of immune stimulating pathway enrichment (Fig. 2e). When we specifically looked at pathway enrichment differences in older and younger macrophages, pathways such as steroid biosynthesis, activation of chemokine receptors and hedgehog signaling were all enriched in the older macrophages (Fig. 2g). Positive regulation of T-cell activation was enriched in younger macrophages. In the older T cells, pathways indicative of active anti-tumor immunity were enriched in younger T cells (Fig. 2g). When we again analyzed an 18-plex circulating inflammatory panel from these rats, many of the markers were again higher in the older rats; interestingly, however, several chemokines that promote macrophage infiltration (CCL2, CCL3, GM-CSF, CXCL1 and CXCL10) were among the most significantly different across age groups (*P* < 0.05 across all markers; Fig. 2h). These findings suggest that age-associated increases in tumor burden are linked more to immune remodeling, including macrophage-associated dysfunction, than to carcinogen susceptibility or mutational differences. Therefore, we examined samples from human patients to evaluate conservation of age-related hormone disposition and tumor-associated changes.

### Aged circulating environment in humans is characterized by E1 predominance with chemokine-enriched chronic inflammation

**Fig. 3:**
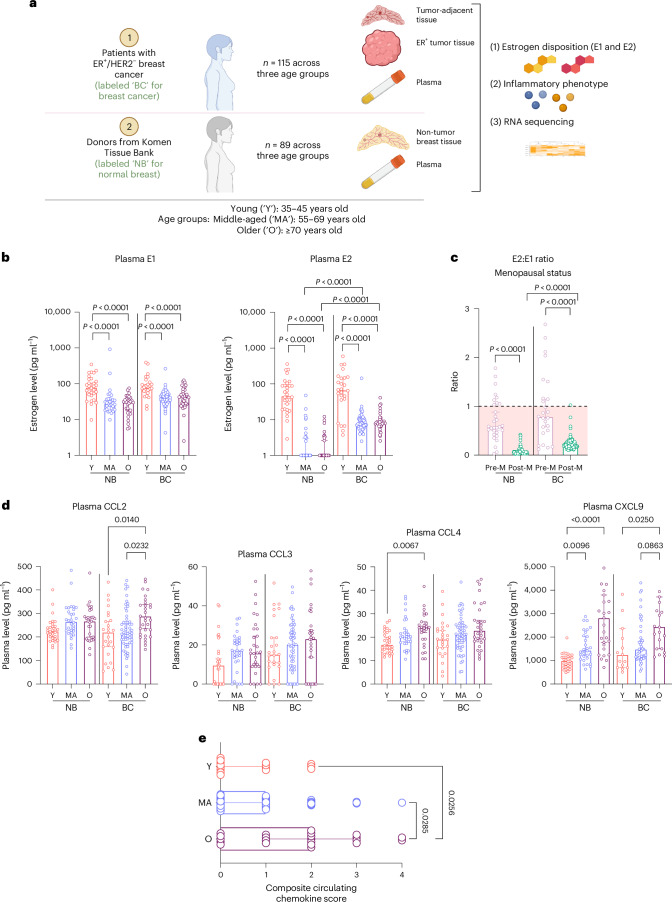
Systemic analysis of estrogen disposition and inflammatory phenotype shows an E1-predominant, chronically inflamed host environment. **a**, Schematic of the key cohorts of human specimens used in the study: cohort of 115 patients with ER^+^ breast cancer with matched plasma, tumor tissue and tumor-adjacent tissue; cohort of 89 donors from the Komen Tissue Bank at Indiana University with matched plasma and non-tumor, normal breast tissue. **b**, Analysis of plasma E1 and E2 from both cohorts. NB indicates data derived from the donors in the Komen Tissue Bank; BC indicates data derived from patients with ER^+^ breast cancer. Age groups indicated by Y (35–45 years old), MA (middle-aged; 55–69 years old) and O (≥70 years old). Data show median with 95% CI. ANOVA with correction for multiple comparisons used for statistical analysis. **c**, E2:E1 ratio computed according to menopausal status. Data show median with 95% CI. ANOVA with correction for multiple comparisons used for statistical analysis. **d**, Circulating levels of CCL2, CCL3, CCL4 and CXCL9. ANOVA with correction for multiple comparisons used for statistical analysis. **e**, The computed composite circulating chemokine score (scale 0–4, patient scored 1 point for each of the four chemokines in **d** that were above two standard deviations of the measured values across age groups) for each age group for patients with ER^+^/HER2^−^ breast cancer. ANOVA with correction for multiple comparisons used for statistical analysis. In all plots, data indicate median with 95% CI. Pre-M, premenopausal; Post-M, postmenopausal. Schematic in **a** created in BioRender; Carleton, N. https://biorender.com/kepjli0 (2026). Source data

We constructed two cohorts to investigate changes in both circulating and local factors with advancing age: (1) matched plasma, tumor tissue and tumor-adjacent tissue from *n* = 115 patients with ER^+^/HER2^−^ breast cancer from the Pitt Biospecimen Core (PBC); and (2) matched plasma and noncancerous, normal breast tissue from *n* = 89 donors from the Komen Tissue Bank. Of the patients with ER^+^/HER2^−^ breast cancer, none received neoadjuvant therapy, and all blood and tissue samples were collected at the time of surgery. We split the samples into the following age groups: young, 35 to 45 years old; middle-aged, 55 to 69 years old; and older, ≥70 years old (Fig. 3a). Although there are a variety of age cutoffs used to define ‘older’ or ‘geriatric’ in clinical practice, 70 was chosen, as there are a number of clinical guidelines currently in place using this cutoff; in reality, aging is a heterogeneous process dependent on a multitude of factors and likely occurs at different rates in different people^19–23^. In general, there were no differences in tumor grade, stage or histology among the three age groups (Supplementary Table 1). In the cohort of donors from the Komen Tissue Bank, there were no differences in donor body mass index (BMI), age at menarche, number of previous pregnancies, race or ethnicity across age groups (Supplementary Table 2). We note that a number of donors in the postmenopausal state had previously used hormone replacement therapy, although no donors were taking this medication at the time of sample collection.

Measurements of plasma E1 and E2 in donors without breast cancer and from patients with ER^+^/HER2^−^ breast cancer showed a loss of E2 in women experiencing postmenopause and E1 as the predominant circulating estrogen (Fig. 3b), consistent with previous reports^24^. There were no differences in E1 or E2 between the middle-aged and older groups of donors and patients. However, we noted elevated circulating E2 and E2:E1 ratio in patients with ER^+^/HER2^−^ breast cancer experiencing postmenopause compared with donors without breast cancer (Fig. 3b,c). From the same plasma samples, we measured a 22-plex panel of inflammatory factors. Both donors without breast cancer and patients with ER^+^/HER2^−^ breast cancer exhibited a marked increase in inflammation with increasing age (Supplementary Fig. 1). Using adjusted linear regression, we identified a series of chemokines, CCL2, CCL3, CCL4 and CXCL9, that were most associated with increasing age across the plasma samples (Fig. 3d), with CCL2 notably significantly elevated in the older patients with ER^+^/HER2^−^ breast cancer. Although CXCL9 has been previously associated with anti-tumor immunity, recent work suggests CXCL9 as a marker of age-related chronic inflammation^25^. These factors, specifically the combined burden of these markers, were highest in the plasma of older donors and patients (Fig. 3e).

### Age-related *HSD17B7* expression linked to E2 enrichment in the local breast TME in patients experiencing postmenopause

**Fig. 4:**
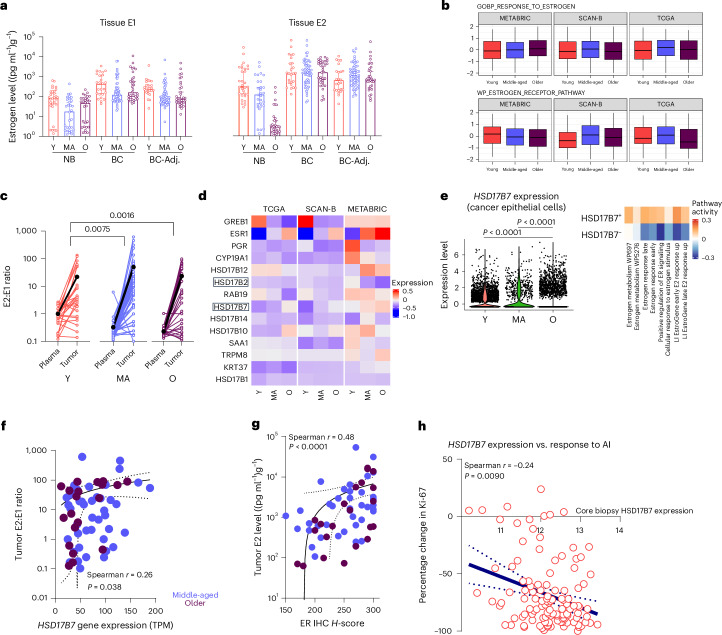
Whereas E1 predominates in the circulating environment, the aged breast local environment is high in E2 and ER, linked to age-related increases in HSD17B7. **a**, Tissue E1 and E2 measured across age groups and among normal breast tissue (*n* = 89), breast cancer tissue (*n* = 115) and matched breast cancer-adjacent tissue (*n* = 115). Comparisons evaluated using ANOVA corrected for multiple comparisons; selected comparisons are shown in figure owing to space constraints. For a full table of comparisons and *P* values, see Supplementary Table 3. Data shown as median with 95% CI. **b**, With local E2 levels equivalent across age groups, downstream estrogen pathway activity shows no trends by age when analyzed using the Mutual Information Concordance Analysis (MICA) pipeline. *N* = 188 patients in Y, *N* = 459 patients in MA and *N* = 251 in O. Data shown as median, interquartile range and range. **c**, E2:E1 ratio comparisons from the plasma to tumor across all ages in the ER^+^ breast cancer tissue. MA (*n* = 53) and O (*n* = 37) patients had significantly higher fold changes in plasma to tumor E2:E1 ratios compared with Y (*n* = 25) patients. ANOVA with correction for multiple comparisons used for statistical analysis. **d**, Profiling of a number of genes using the MICA pipeline to examine expression patterns across age groups using TCGA, METABRIC and SCAN-B bulk RNA-seq datasets. Data show that only *HSD17B7* expression (increasing expression with increasing age) and *HSD17B2* (decreasing expression with increasing age) were found to have consistent trends according to MICA. **e**, *HSD17B7* levels were highest in older patients, as corroborated by single-cell RNA sequencing (scRNA-seq) data from ref. ^68^ (*n* = 3 young, *n* = 4 middle-aged and *n* = 3 older patients). Violin plots show single-cell gene expression values, including zero-expression cells, consistent with standard visualization of scRNA-seq data. Heat map showing selected estrogen pathway enrichment in HSD17B7^+^ cancer epithelial cells. **f**, Scatter plot comparing *HSD17B7* expression levels (as measured using bulk RNA-seq) and measured tumor E2:E1 ratios showing a positive correlation (two-sided Spearman *r* = 0.24, *P* = 0.03). Dot color indicates age group. **g**, Scatter plot comparing ER IHC *H*-score and tumor levels of E2 ((pg ml^−1^) g^−1^ of tissue) showing a positive correlation (two-sided Spearman *r* = 0.45, *P* < 0.0001). **h**, Using data from the POETIC trial^32^, scatter plot compares baseline level of *HSD17B7* expression in the core biopsy against the percentage change in Ki-67 after neoadjuvant endocrine therapy (*n* = 118; two-sided Spearman *r* = −0.24, *P* = 0.009). TPM, transcripts per million; IHC, immunohistochemistry; AI, aromatase inhibitor. Source data

**Extended Data Fig. 3:**
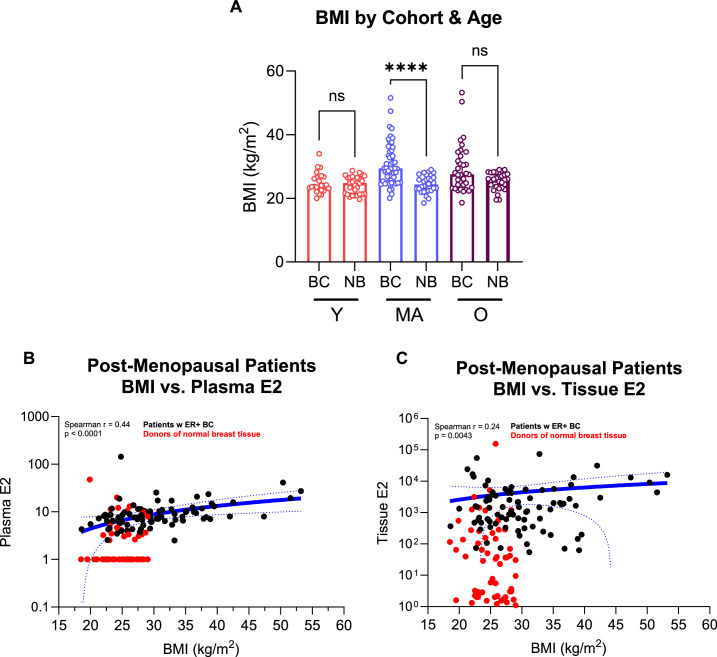
Association between host BMI estrogen levels. (**A**) BMI comparison between the patients with ER+ breast cancer and the donors from the Komen Tissue Bank. **** indicates p-value < 0.0001. N = 25 for Y-BC, N = 30 for Y-NB, N = 57 for MA-BC, N = 30 for MA-NB, N = 37 for O-BC, N = 29 for O-NB. Mann Whitney testing use for statistical testing within age groups. Data show median with 95% CI. (**B**) BMI was positively associated with circulating E2 level in post-menopausal patients and donors (Spearman r = 0.44, p < 0.0001; linear regression p = 0.0113 for slope significantly nonzero). (**C**) BMI was weakly associated with tissue levels of E2 in post-menopausal patients and donors (Spearman r = 0.24, p = 0.0043; linear regression p = 0.37 for slope significantly nonzero). Source Data

Next, we investigated estrogen disposition in the normal breast microenvironment from Komen Tissue Bank donors and in the TME from patients with breast cancer and their adjacent tissues. In the normal breast, E2 in the breast of older donors was significantly lower than E1 (*P* = 0.0033) (Fig. 4a), mirroring the difference observed in plasma. By stark contrast, despite the vast plasma differences in E1 and E2 with menopause and despite similar differences in the normal breast, E2 was the predominant estrogen in the breast for patients with ER^+^ disease, with marked increases over E1 across all age groups, including older patients. In the tumor tissue itself, E2 was similar across all age groups (*P* = 0.99) (Fig. 4a). Of note, we observed differences in BMI across the cohorts (Extended Data Fig. 3a): although BMI was expectedly associated with plasma E2 in women experiencing postmenopause (Extended Data Fig. 3b), it was also weakly associated with tissue E2 (Extended Data Fig. 3c). There were no differences in plasma or tumor estrogen levels in patients with invasive ductal carcinoma (IDC) versus those with invasive lobular carcinoma (Supplementary Fig. 2).

Given the high E2 in the breast cancer TME, we hypothesized that ER activity should remain high in this setting. To test this, we examined a panel of estrogen-related pathways in early-stage ER^+^/HER2^−^ breast cancer in the TCGA, METABRIC and SCAN-B datasets. We used MICA, a statistical approach that evaluates consistent trends in pathway activity (or gene expression) within the three age groups across the three RNA-seq datasets^26^. Concordant with the local E2 levels, downstream activity in estrogen-related pathways was not different across age groups, indicating again that tumors in middle-aged and older patients have just as much estrogen signaling activity compared with those in younger patients (Fig. 4b and Supplementary Fig. 3). When we compared plasma E2:E1 ratio against that found in the tumor tissue, we found that middle-aged and older patients had a significantly higher fold change increase compared with that found in younger patients (Fig. 4c), indicating that the tumors arising in the aged breast either synthesize local E2 de novo or use a mechanism to convert E1 to E2.

**Extended Data Fig. 4:**
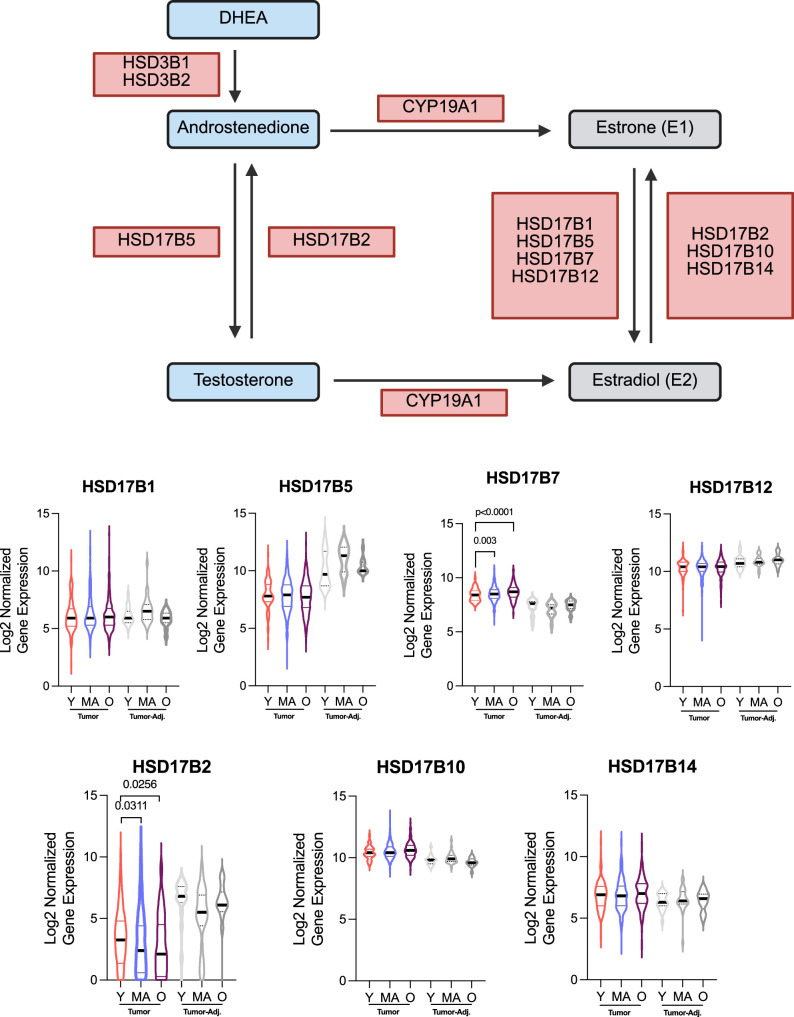
Analysis of hydroxysteroid enzymes. (**A**) Schematic depicting the hydroxysteroid dehydrogenase (HSD) enzymes responsible for E1 to E2 conversion and E2 to E1 conversion. (**B**) Analysis of HSD enzyme gene expression levels across ages in tumor and tumor-adjacent tissue using the TCGA database. ANOVA with correction for multiple comparisons used for statistical testing.

To decipher how E2 is synthesized in the breast, we examined a panel of genes encoding enzymes involved in estrogen synthesis and conversion, again using the MICA method. Among all of the hydroxysteroid enzymes involved in E1-to-E2 and E2-to-E1 interconversion (schematic in Extended Data Fig. 4a), *HSD17B7*, which is involved in E1-to-E2 conversion, was found to be consistently increased with age (*P* = 0.012), and *HSD17B2*, involved in E2-to-E1 conversion, was found to be consistently decreased (*P* = 0.0061) with age across all three datasets, with no trends for other hydroxysteroid enzymes (Fig. 4d). This finding was restricted to tumor tissue, where *HSD17B7* expression was significantly higher when compared with that in normal-adjacent tissues (Extended Data Fig. 4b). Aromatase (*CYP19A1*) was not different across age groups. We found similar age-related increases in *HSD17B7* expression using ER^+^/HER2^−^ breast cancer scRNA-seq data^27^, specifically in the cancer epithelial cells (Fig. 4e); indeed, HSD17B7^+^ cancer epithelial cells had increased estrogen pathway activity compared with HSD17B7^−^ cancer epithelial cells.

**Extended Data Fig. 5:**
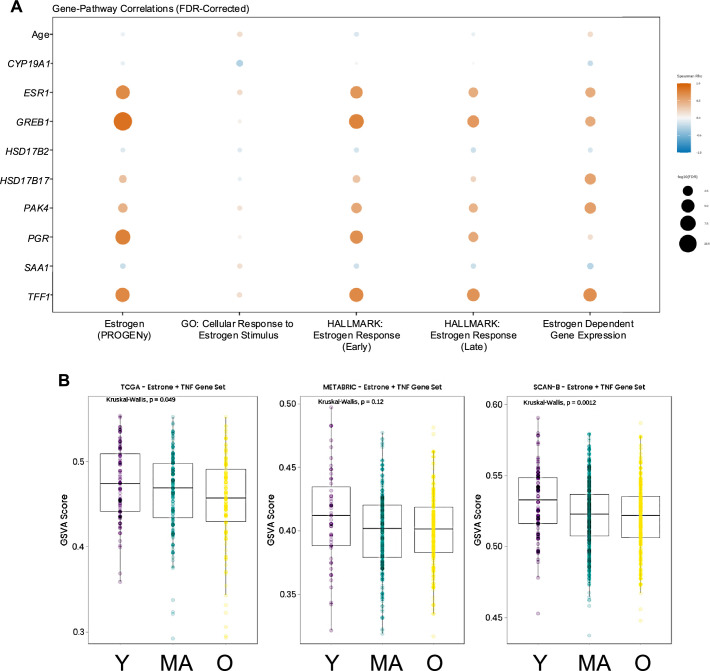
Analysis of estrogen-related pathways across ages. (**A**) Correlation dot plot using the bulk RNA-seq data generated in this study showing correlation between several estrogen-related pathways vs. estrogen-related genes and enzymes. (**B**) Analysis of an Estrone+TNF signature (ref. ^27^) across ages in TCGA, METABRIC, and SCAN-B.

In the same tissues in which estrogen disposition was measured, we performed bulk RNA-seq, which showed that *HSD17B7* expression was positively correlated with a number of estrogen-related pathways (Extended Data Fig. 5a). Importantly, *HSD17B2* expression was negatively correlated with estrogen pathway activity. A derived signature of E1 and inflammatory (E1+TNF) activity^24^ showed consistent decreases with age in TCGA (*P* = 0.049), METABRIC (*P* = 0.12) and SCAN-B (*P* = 0.0012) (Extended Data Fig. 5b). Analysis of the association between *HSD17B7* expression and postmenopausal tumor E2:E1 ratio showed that as enzyme gene expression increased, so too did the balance of E2 to E1 in the TME (Spearman *r* = 0.26, *P* = 0.038) (Fig. 4f).

**Extended Data Fig. 6:**
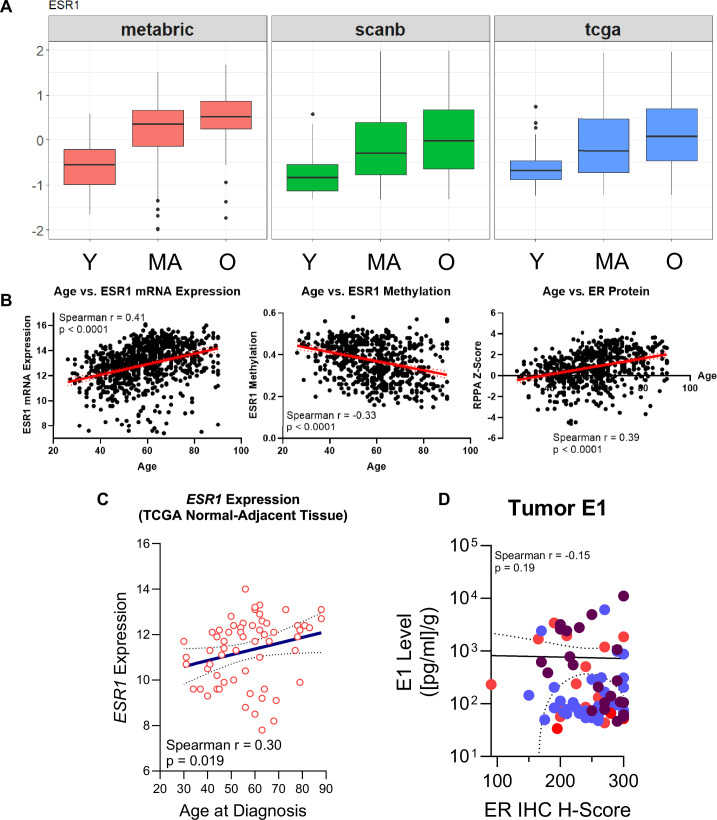
ESR1 changes with age. (**A**) ESR1 expression levels across age in the TCGA, METABRIC, and SCAN-B datasets. (**B**) Scatter plots showing Age vs. ESR1 mRNA expression, ESR1 methylation, and ER protein (all using the TCGA database). (**C**) Scatter plot showing Age vs. ESR1 expression in TCGA normal tumor-adjacent tissues. (**D**) Scatter plot showing ER IHC H-score vs. tumor E1 levels. Source Data

Consistent with previous reports, we found that *ESR1* gene expression increases with age^28,29^, a finding compounded with increasing hypomethylation and concomitant increases in ER protein (Fig. 4d and Extended Data Fig. 6a,b). Intriguingly, these changes were also observed with *ESR1* expression in normal-adjacent tissues from TCGA (Extended Data Fig. 6c), suggesting that *ESR1* is likely sensitized to low plasma and breast E2 levels in women experiencing postmenopause such that even the normal, noncancerous breast harbors higher relative levels of ER than premenopausal counterparts. Uniquely within the aged TME, however, we observed that not only are there locally high E2 levels, but these tumors also retain high levels of ER positivity: tumors with the highest ER *H*-score also had high levels of tumor E2 (Spearman *r* = 0.48, *P* < 0.0001; Fig. 4g), whereas there was no association between ER *H*-score and measured E1 levels (Extended Data Fig. 6d). The estrogen disposition of these tumors also enhances sensitivity to aromatase inhibition, where high levels of *HSD17B7* expression in the tumors of patients in the POETIC trial testing neoadjuvant aromatase inhibition^30^ were associated with the largest percentage change in Ki-67 (Spearman *r* = −0.24, *P* = 0.0090) (Fig. 4h).

**Fig. 5:**
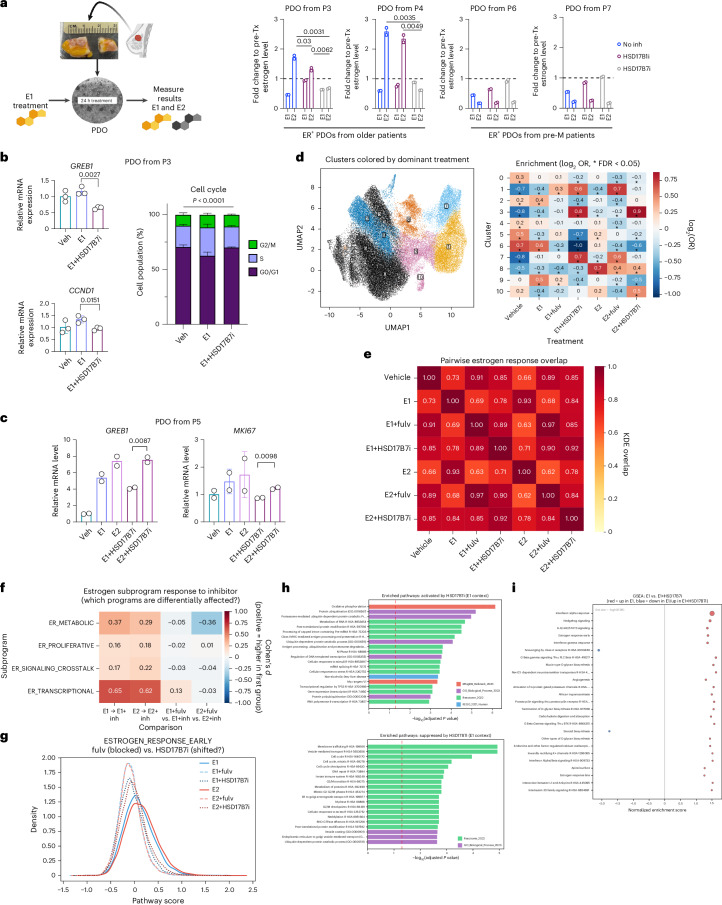
Inhibition of HSD17B7 prevents E1-to-E2 conversion and decreases transcriptional programs of ER responses in PDOs from older patients with ER^+^/HER2^−^ breast cancer. **a**, We treated PDOs with E1 and evaluated the degree to which that E1 was converted to E2 under the conditions of no inhibitor present, in the presence of an HSD17B1 inhibitor or in the presence of an HSD17B7 inhibitor. PDOs from patients 3 and 4 (P3 and P4, respectively) were derived from older patients with ER^+^/HER2^−^ luminal breast cancer, whereas PDOs from patients 6 and 7 (P6 and P7, respectively) were derived from younger patients with ER^+^/HER2^−^ breast cancer experiencing perimenopause (see Supplementary Table 3 for full clinical characteristics of the PDOs used in this study). *N* = 2 biological replicates plotted. Data shown as median with 95% CI. **b**, In the PDO from patient 3 (patient age 78 with luminal breast cancer), HSD17B7 inhibition after E1 treatment decreased *GREB1* and *CCND1* gene expression (ANOVA with correction for multiple comparisons) and decreased the proportion of PDO cells in the S phase (statistical tests performed using two-way ANOVA). *N* = 3 biological replicates plotted. Data shown as median and 95% CI in the bar plot and mean ± s.e.m. in the cell cycle plot. **c**, In the PDO from patient 5 (patient age 83 with luminal breast cancer), HSD17B7 inhibition after E1 but not E2 treatment led to a decrease in *GREB1* and *MKI67* gene expression. *N* = 2 biological replicates plotted. Data shown as median with 95% CI. ANOVA with correction for multiple comparisons used for statistical analysis. **d**, PDOs were treated for 24 h under the following conditions: vehicle control, E1 (1.5 nM), E2 (1.5 nM), E1 (1.5 nM) plus HSD17B7 inhibitor (4 µM), E2 (1.5 nM) plus HSD17B7 inhibitor (4 µM), E1 (1.5 nM) plus fulvestrant (1 µM) and E2 (1.5 nM) plus fulvestrant (1 µM). Cells were subjected to scRNA-seq to assess estrogen response. Uniform Manifold Approximation and Projection (UMAP) shows dominant clusters following treatment. **e**, Heat map showing the degree of estrogen response overlap. **f**, We assessed which estrogen response subprograms were most affected by the inhibitor conditions. The HSD17B7 inhibitor inhibited ER transcriptional and metabolic responses. Heat map shows values of Cohen’s *d*, where positive values indicate higher levels in the first group of any individual column. **g**, Density plots show changes in response dynamics using the Estrogen_Response_Early pathway. Whereas E1 and E2 showed similar responses, the HSD17B7 inhibitor showed a high degree of similarity to fulvestrant. **h**, Pathway enrichment analysis showing that pathways related to stress responses are activated with E1 + HSD17B7 inhibitor versus E1 alone. Pathways related to cell cycle, proliferation and transcriptional machinery are suppressed. **i**, Gene Set Enrichment Analysis (GSEA) indicating pathways enriched in the E1 and E1 + HSD17B7 inhibitor conditions. Pre-Tx, pretreatment; OR, odds ratio. Schematic in **a** created in BioRender; Carleton, N. https://biorender.com/pb3xn85 (2026). KDE, kernel density estimator; FDR, false discovery rate. Source data

**Extended Data Fig. 7:**
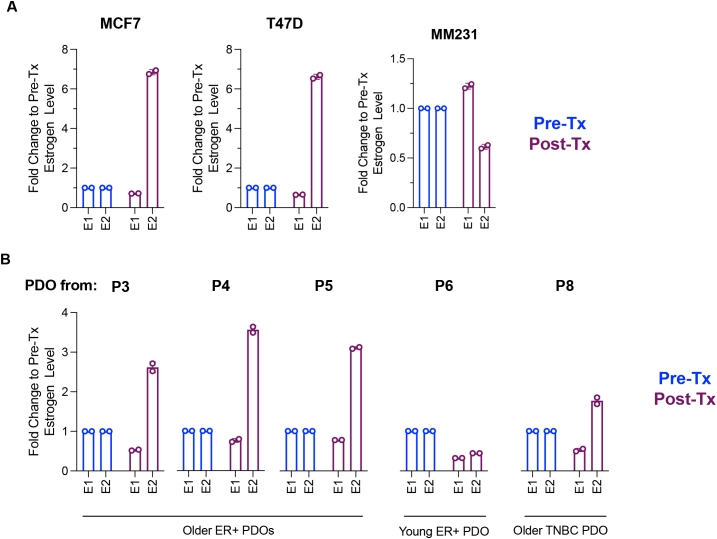
E1 treatment of cell lines and PDOs. (**A** and **B**) We treated cell lines (MCF7, T47D, and MM231) as well as a panel of patient-derived organoids (PDOs) with E1 and evaluated the degree to which that E1 was converted to E2. All post-treatment data is normalized to the pre-treatmsent levels. PDOs from Patient 3 (P3), 4 (P4), 5 (P5), 6 (P6), and 8 (P8) were used in this experiment (see Table S3 in this document for full clinical characteristics of the PDOs used in this study). P3, P4, and P5 were PDOs derived from older patients with ER+ breast cancer; P6 was a PDO derived from a younger, pre-menopausal patient with ER+ breast cancer; and P8 was a PDO derived from an older patient with TNBC. N = 2 biological replicates across all experiments. Source Data

We then used a panel of breast cancer cell lines and prospectively collected patient-derived organoids (PDOs) to test their ability to convert E1 to E2 (Fig. 5a). Treatment with physiologically relevant levels of E1 (500 pg ml^−1^) for 24 h resulted in conversion of E1 to E2 in ER^+^ but not ER^−^ human breast cancer cell lines, reproducing previous reports^31^ (Extended Data Fig. 7a). We then tested a series of PDOs from older patients with ER^+^/HER2^−^ breast cancer, a PDO from a younger patient with ER^+^/HER2^−^ breast cancer in perimenopause and a PDO from an older patient with triple-negative breast cancer (TNBC) (full clinical characteristics of the PDOs used throughout this study can be found in Supplementary Table 4). ER^+^/HER2^−^ PDOs from older patients converted significantly higher levels of E1 to E2 than the PDO from a younger patient and the PDO from the older patient with TNBC (Extended Data Fig. 7b). Given the age-related increase in HSD17B7 in ER^+^/HER2^−^ breast cancer in older patients, we tested the role of HSD17B7 inhibition in preventing conversion of E1 to E2 in the PDOs using a small molecule inhibitor^32–35^ (compound 10). When we treated the PDOs with this HSD17B7 inhibitor (and used an inhibitor of HSD17B1, CC-156, as this enzyme does not increase with age though is also responsible for E1-to-E2 conversion), the HSD17B7 inhibitor decreased E1-to-E2 conversion in the ER^+^/HER2^−^ PDOs from older patients but had little effect on the other PDOs (Fig. 5a). The HSD17B1 inhibitor had little to no effect on E1-to-E2 conversion. Given the HSD17B7 inhibitor’s ability to decrease E1-to-E2 conversion, we tested its effect on downstream activity in the PDOs derived from older patients. In the PDO derived from P3 (an older patient with luminal IDC), treatment for 24 h with E1 and the HSD17B7 inhibitor significantly decreased *GREB1* (ER response gene) and *CCND1* expression (Fig. 5b). Concordantly, the inhibitor significantly decreased the number of cells in the S phase, restoring them to the G0/G1 phase (Fig. 5b). In a second PDO from an older patient (an older patient with luminal IDC), we again observed similar induction of *GREB1* with both E1 and E2. The inhibitor was most efficacious when it was given in combination with E1 and not E2, with significant decreases in *GREB1* and *MKI67* gene expression with E1 and the inhibitor (Fig. 5c).

**Extended Data Fig. 8:**
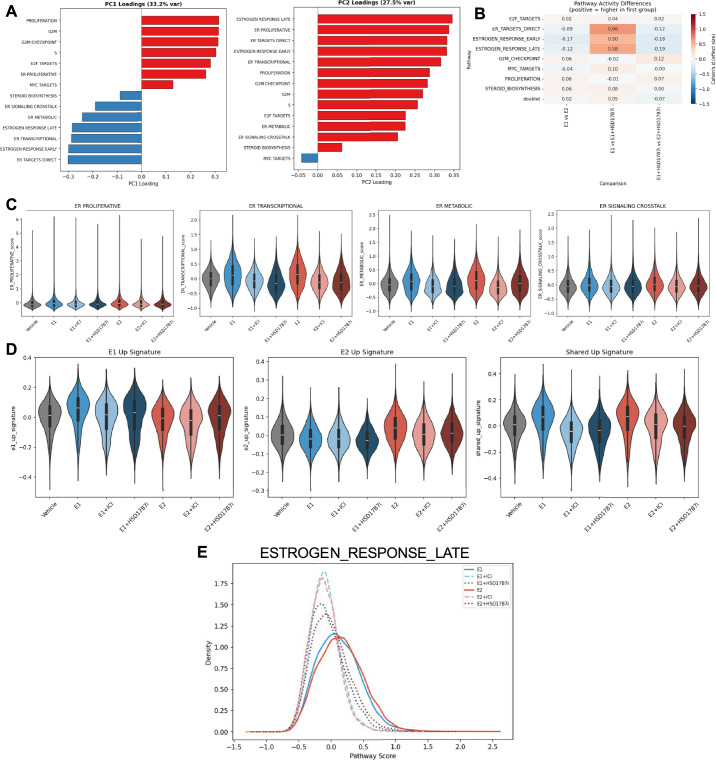
scRNA-seq of E1, E2, and anti-estrogen treated-PDO from an older patient indicate antiproliferative effects of the HSD17B7i. (**A**) PCA showing that proliferation pathways and estrogen-related signaling pathways dictate PC1 and PC2. (**B**) Heatmap showing an expected overlap in E1 and E2 signaling pathways with divergence in estrogen response early and late with the addition of the HSD17B7 inhibitor. (**C**) Shows the score of four estrogen-related subprograms across all seven treatment conditions. Plots quantify the score for each single cell from the PDO treated within a given condition. (**D**) Shows the enrichment of an E1, E2, and shared signature across all seven treatment conditions. Plots quantify the score for each single cell from the PDO treated within a given condition. (**E**) Density plot showing pathway score of ESTROGEN_RESPONSE_LATE across treatment conditions.

To fully characterize the downstream action of the HSD17B7 inhibitor, we carried out scRNA-seq of the PDO from P5 after treatment with E1 and E2, and with and without the HSD17B7 inhibitor and fulvestrant. Leiden clustering showed limited heterogeneity given that all cells were from a single PDO, although differences across clusters were expectedly dominated by proliferation and estrogen response signatures (Fig. 5d and Extended Data Fig. 8a). When evaluating the degree of estrogen response overlap between E1 and E2 and the inhibitor conditions, E1 and E2 showed high degrees of estrogen response, whereas E1 versus E1 + HSD17B7 inhibitor showed relative dissimilarity (Fig. 5e). Interestingly, E1 + HSD17B7 inhibitor showed high degrees of overlap with E2 + HSD17B7 inhibitor and both conditions involving fulvestrant. Further investigation of the estrogen-related subprograms most affected by the HSD17B7 inhibitor indicated perturbation of ER transcriptional and metabolic activities, although again the inhibitor showed similar effects when paired with both E1 and E2 (Fig. 5f and Extended Data Fig. 8b,c). We hypothesized that this similarity was due to E2 cycling back to E1 in this in vitro setting. When we looked at E1- and E2-specific signatures across conditions, we observed that E2 + HSD17B7 inhibitor showed higher E1-dominant signature than E2 alone (*d* = −0.11, *P* = 5.0 × 10^−15^) but lower E2-dominant signature than E2 alone (*d* = +0.34, *P* = 7.5 × 10^−96^) (Extended Data Fig. 8d), supporting this hypothesis. Nevertheless, the E1 + HSD17B7-treated PDO cells, compared with E2 + HSD17B7 inhibitor-treated PDO cells, were more likely to behave as if they were treated with fulvestrant (Fig. 5g). Pathway enrichment analysis showed that stress-related pathways, such as oxidative phosphorylation, were activated, and pathways related to proliferation and cell cycle activity were suppressed with the addition of the HSD17B7 inhibitor to E1 (Fig. 5h,i).

These data support an aged TME characterized by both high ER and high E2 levels, a process that is targetable in PDOs through HSD17B7 inhibition.

### Aged breast TME exhibits features of chronic inflammation but is characterized by marked immune dysfunction

**Fig. 6:**
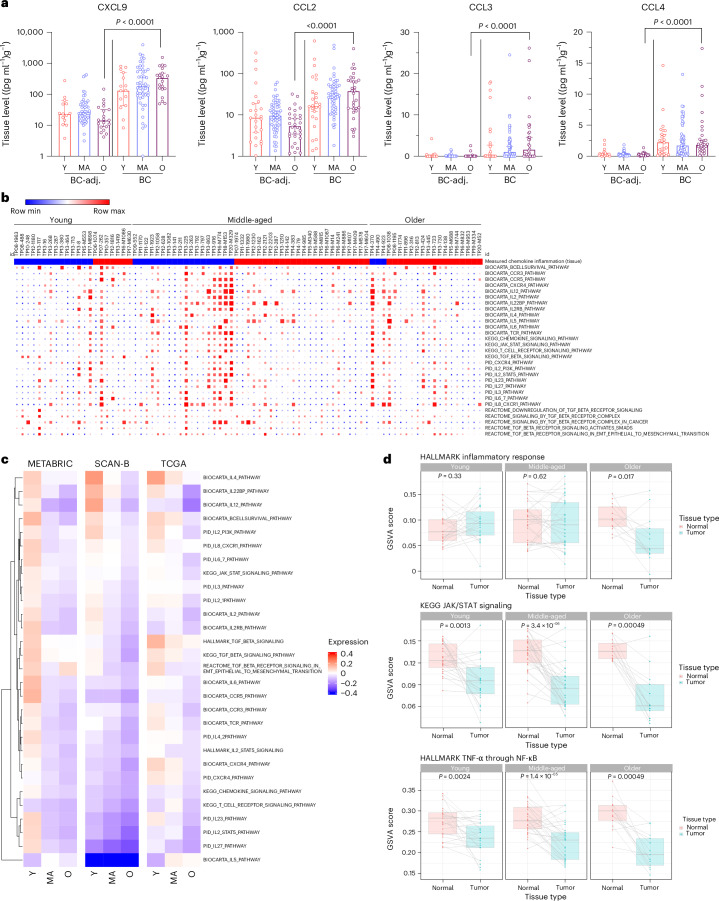
The aged breast TME recapitulates a chemokine-enriched chronic inflammation. **a**, Tumor lysates were analyzed using the same Luminex inflammatory panel. Similar to the circulating environment, the aged breast TME was characterized by high levels of chemokines, most notably CCL2. ANOVA used for comparison across multiple groups with correction for multiple comparisons. *N* = 25 for Y, *N* = 53 for MA and *N* = 37 for O. Data shown as median with 95% CI. **b**, In the same tumors (and tumor lysates) that were analyzed for the inflammatory panel, we also performed bulk RNA-seq to connect the measured inflammation from the TME to the cell-intrinsic signaling patterns. The heat map shows that tumors that had the highest levels of chemokine-defined inflammatory scores (indicated by the ‘Measured chemokine inflammation’ row; red values indicate patients who had chemokine scores higher than the median across all patients; blue indicates patients who had chemokine scores below the median) also had the lowest levels of inflammatory pathway enrichment or the highest level of immune dysfunctionality. **c**, Heat map showing a series of inflammation-related pathways from MSigDB, indicating that across all three bulk RNA-seq datasets, older patients consistently had the lowest levels of pathway enrichment. **d**, Comparisons between normal (tumor adjacent) and tumor tissue from TCGA according to enrichment of several HALLMARK pathways. Tumor tissue, but not normal tumor-adjacent tissue, from older patients showed the lowest level of pathway enrichment. *P* values indicated in figure. *N* = 21 matched pairs for Y, *N* = 27 matched pairs for MA and *N* = 13 for O. Data shown as median, interquartile range and range. BC-adj., breast cancer-adjacent. Source data

Given the chronic inflammatory phenotype observed in the plasma, next, we investigated whether the breast microenvironment showed similar changes associated with age. Although there was evidence of a modest chronic inflammatory response in normal breast tissue with advancing age, older patients with ER^+^/HER2^−^ breast cancer harbored high levels of a chemokine-enriched inflammatory TME compared with the tumor-adjacent tissue (Fig. 6a and Supplementary Fig. 4). This age-associated inflammatory environment was again most notably characterized by elevated levels of chemokines (although many factors, such as IL-6, IFN-γ, IL-1β and IL-8, did not change with age), indicating that the aged TME was chronically inflamed relative to the TME of both younger and middle-aged patients. Next, we examined the degree to which the chemokine-inflamed TME shaped immune signaling in our patient cohort. We first segregated patients across age groups according to chemokine-defined inflammatory scores. Patients with the highest levels of chemokine inflammation in the TME harbored the lowest levels of immune pathway activation, indicating broad immune dysfunction in these patients, which was most prominent in older patients (Fig. 6b).

Despite the inflamed nature of the TME, when we used the MICA pipeline to profile all inflammation-related pathways within MSigDB, we observed broad age-related decreases in key inflammatory and immune-activating pathways, again with the lowest levels of pathway enrichment in older patients (Fig. 6c). Among the pathways differentially enriched, those related to reactive oxygen species and oxidative phosphorylation were consistently enriched in older versus both middle-aged and younger patients (Supplementary Fig. 5). However, pathways related to inflammatory signaling, such as TNF-α through NF-κB, HALLMARK inflammatory response and IL-6–JAK/STAT, were consistently enriched in younger and middle-aged patients compared with older patients (Supplementary Fig. 5). In TCGA with matched tumor and tumor-adjacent tissues, although these pathways increased with age in the tumor-adjacent tissues, the tumors from older patients again exhibited the lowest scores (Fig. 6d). Overall, although we observed a prominent inflammatory environment within the TME of older patients with ER^+^/HER2^−^ breast cancer, these data suggest that the chronic inflammation (that is, significant levels of chemokines present in the TME itself) is associated with immune dysfunction and suppression.

### ER^+^/HER2^−^ tumors from older patients are characterized by immunosuppressive macrophages

**Fig. 7:**
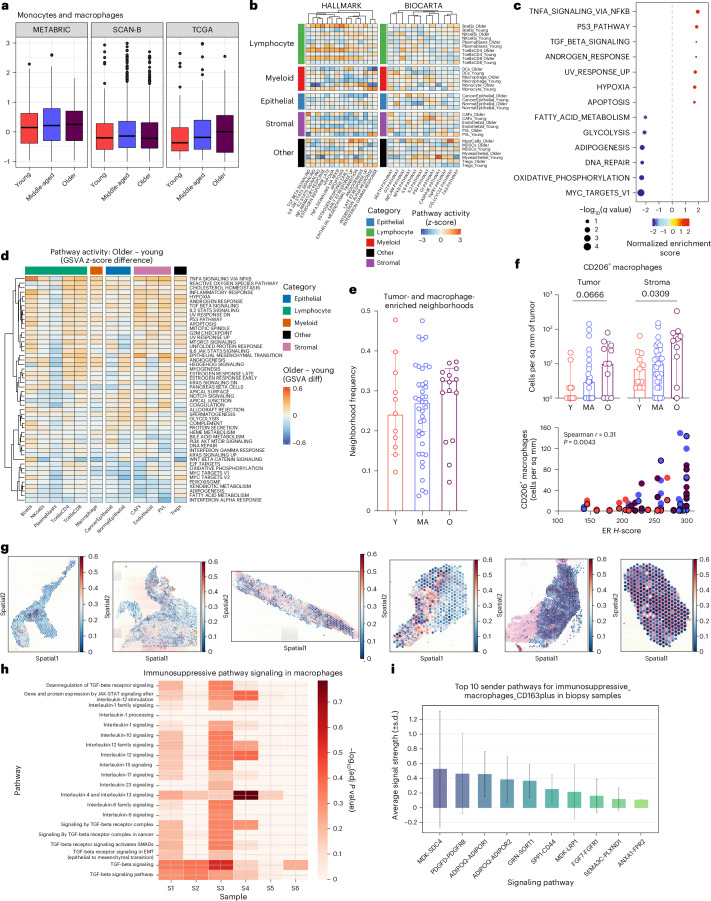
A chemokine-rich aged TME is associated with accumulation of immunosuppressive macrophages. **a**, Using immune deconvoluted data from TCGA, METABRIC and SCAN-B, the MICA pipeline identified that cells from the monocytic and macrophage lineages showed consistent increases with age. *N* = 188 patients in Y, *N* = 459 patients in MA and *N* = 251 in O. **b**, Using publicly available scRNA-seq data (ref. ^68^; data used in Fig. 6b–d; only used tumors that were treatment-naive ER^+^/HER2^−^), macrophages and monocytes were among the most transcriptomically different across age groups. **c**, Differentially expressed gene analysis with subsequent pathway analysis using the differentially expressed genes between macrophages from older patients and younger patients showed an enrichment of TNF-α and TGF-β signaling in older patients (indicated by red dots). **d**, Broader pathway analysis across epithelial, lymphoid, myeloid and stromal cells. **e**, Using previously generated multispectral immunohistochemistry (mIHC) data from our laboratory^39^, we reanalyzed the neighborhood analysis data from ER^+^/HER2^−^ tumors according to age. There was a higher frequency of tumor- and macrophage-enriched neighborhoods in older patients. Representative mIHC images of these neighborhoods shown in Extended Data Fig. 10. *N* = 11 for Y, *N* = 39 for MA and *N* = 15 for O. Data shown as median with 95% CI. **f**, Using the mIHC data from ref. ^39^, CD206^+^ macrophages were higher in both the tumor and stromal regions in older patients with ER^+^/HER2^−^ breast cancer. *N* = 11 for Y, *N* = 37 for MA and *N* = 11 for O in both tumor and stromal regions. Data shown as median with 95% CI. ANOVA with multiple comparisons used for statistical tests. We observed a positive association between tumor ER *H*-score and the amount of CD206^+^ macrophages present (Spearman *r* = 0.31, *P* = 0.0043). Dots colored according to age. Dots with black outline indicate cells from stromal regions, whereas those without a border indicate cells from tumor regions. **g**, Spatial transcriptomics and proteomics completed on a cohort of older patients with treatment-naive ER^+^/HER2^−^ breast cancer from a clinical trial we ran^38^ (NCT05914792) showed detection of immunosuppressive macrophages in the TME in proximity to cancer cell-enriched regions. Each image (*n* = 6) represents a core biopsy from each of the six specimens included in the analysis. **h**, Further characterization of these immunosuppressive macrophages showed enrichment of pathways related to IL-4 and IL-13 signaling and a lack of enrichment of anti-tumor, immune-responsive pathways. **i**, Cell–cell communication analysis was performed using Communication analysis by Optimal Transport (COMMOT) to define ligand–receptor interactions and specifically unidirectional secreted signals from the immunosuppressive macrophages. Source data

Given the chemokine-enriched nature of both the circulating and local inflammatory environments, we hypothesized that macrophages may be a prominent component of and may shape the aged TME. Indeed, the proportion of cells from the monocytic lineage increased with age when examining deconvoluted data from bulk RNA-seq datasets (*P* value for trend derived from MICA = 0.0012; Fig. 7a). We then investigated publicly available scRNA-seq data^27^, only including those tumors that were early stage, untreated and ER^+^/HER2^−^ (Supplementary Fig. 6). Across a broad range of immune-related pathways, including those that are typically found primarily in lymphocytes and myeloid cells, tumor-associated macrophages (TAMs) in older patients were among the most distinct from those in their younger (Fig. 7b) and middle-aged (Supplementary Fig. 6) counterparts. Notably, most immune-activating pathways were lower in TAMs from older patients; only pathways such as hedgehog signaling, a pathway associated with immunosuppressive features, were uniquely different in TAMs from older patients (Fig. 7b and Supplementary Fig. 6).

We then analyzed differentially expressed genes in TAMs from older patients compared with younger and middle-aged patients, which showed that although there was broad overlap in genes enriched in TAMs from younger and middle-aged patients, the TAMs from older patients seemed to harbor a unique set of enriched genes (Supplementary Fig. 6). Analysis on the differentially expressed genes indicated that pathways associated with chronic inflammation, such as TNF-α through NF-κB and TGF-β signaling, were enriched in TAMs from older patients compared with both younger and middle-aged patients (Fig. 7c). TNF-α pathway activation was unexpected but likely related to damage-associated molecular pattern activity and macrophage Toll-like receptor signaling in the aged TME (Supplementary Fig. 6). Further subtyping of the TAMs according to the paradigm described by Ma et al.^36^ showed that although TAMs from older patients exhibited features of mapping to multiple TAM categories, these cells consistently had prominent markers associated with the establishment of an immunosuppressive TME, including chemokine signaling (most closely associated with inflammation TAMs), *MRC1* (CD206) expression and *CD274* (PD-L1) expression, which were collectively elevated in the middle-aged and older patients (Supplementary Fig. 6). Characterization of pathway activation across immune compartments indicated again that immune cells in younger patients had higher levels of interferon signaling, whereas the immune cells (especially TAMs) in older patients blunted the anti-tumor response (Fig. 7d).

**Extended Data Fig. 9:**
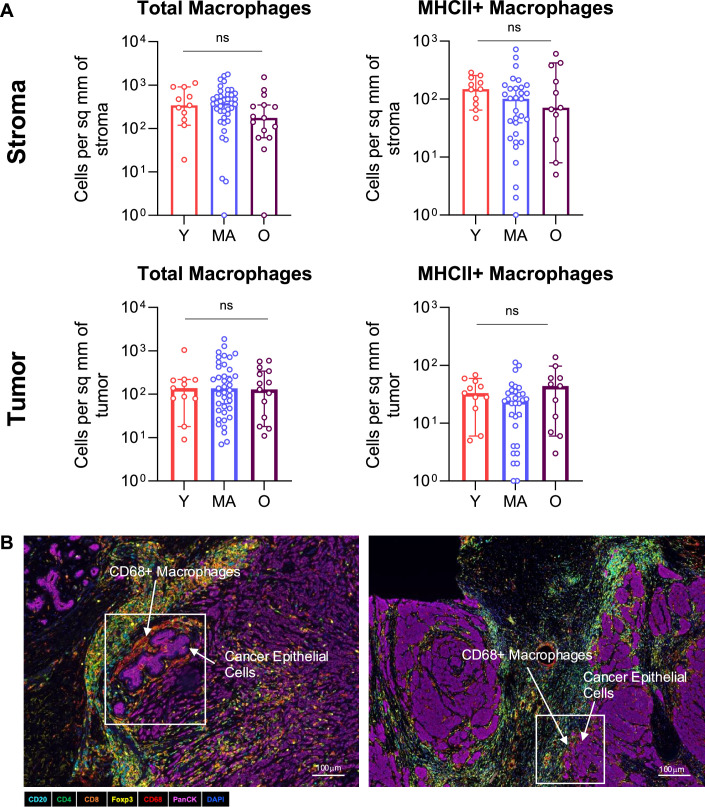
Multispectral immunohistochemistry (mIHC) data showing total macrophage and MHCII+ macrophage quantification. (**A**) Using mIHC data from ref. ^35^ (Onkar et al.), we reanalyzed the data according to age group, showing little difference in total macrophage and MHCII+ macrophage levels across age groups. N = 11 for Y, N = 39 for MA, and N = 15 for O. ANOVA with correction for multiple comparisons used for statistical testing. (**B**) Represent mIHC images showing macrophage-rich neighborhoods, as indicated with the white box. Source Data

To validate and support the scRNA-seq findings, we analyzed two additional datasets that included mIHC stained slides from a cohort of patients with ER^+^/HER2^−^ breast cancer^37^ and spatial transcriptomics from a cohort of older patients with ER^+^/HER2^−^ breast cancer enrolled on a clinical trial^38^ (NCT05914792). Compared with that in younger and middle-aged patients, although total macrophages did not change with age (either in the stroma or within the tumor; Extended Data Fig. 9a), the proportion of tumor- and macrophage-enriched neighborhoods increased in the TME in older patients (Fig. 7e; representative images of tumor- and macrophage-enriched neighborhoods denoted with white box in Extended Data Fig. 9b), in part attributable to significant increases in tumor and stromal CD206^+^ TAMs with age (Fig. 7f) without any age-associated changes to the MHCII^+^ TAM infiltrate (Extended Data Fig. 9a). Importantly, the proportion of CD206^+^ TAMs was significantly associated with the degree of ER positivity in the tumor (Spearman *r* = 0.31; *P* = 0.0043) (Fig. 7f). We then examined six diagnostic core biopsy specimens from older patients with ER^+^/HER2^−^ breast cancer for the presence of immunosuppressive macrophages. In general, we observed immunosuppressive macrophages across all six specimens (heat map showing cellular location of immunosuppressive macrophages in Fig. 7g), and these cells were primarily localized to the adjacent stromal tissue abutting tumor nests. Characterization of these macrophages indicated enrichment of IL-4 and IL-13 signaling and a lack of enrichment in anti-tumor signaling axes (Fig. 7h). Unidirectional sender signal analysis showed that anti-inflammatory molecules such as SPP1, MDK and PDGF-C were among the top secreted markers from these cells.

Collectively, these data suggest that immunosuppressive TAMs accumulate in the TME of older patients with ER^+^/HER2^−^ breast cancer and are associated with an immunosuppressive transcriptional program and features of reduced adaptive immune signaling.

### Aged TME linked to immunosuppressive macrophage state

**Fig. 8:**
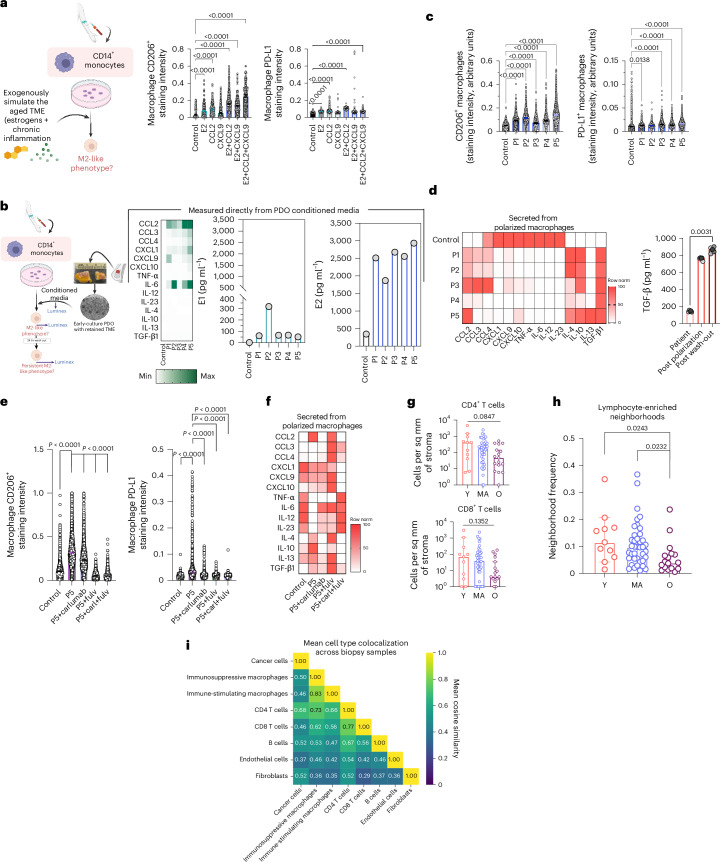
Aged TME features polarize macrophages to an immunosuppressive state that is targetable through the CCL2 axis or through endocrine therapies. **a**, We first exogenously treated macrophages with components of the aged TME (including E2, CCL2, CXCL9 or their combination) and measured CD206 or PD-L1 staining intensity via immunofluorescence. Data show median with 95% CI. ANOVA with correction for multiple comparisons used for statistical tests. Data shown as median with 95% CI. **b**, We then extracted conditioned media from just-in-culture PDOs from patients 1–5 (P1–P5; full clinical characteristics of the collected PDOs can be found in Supplementary Table 3). Heat map shows non-normalized values of inflammatory markers present in the conditioned media or the control media (baseline breast organoid media) for each patient. We also measured E1 and E2 in the conditioned media, showing high levels of E2 in the milieu of the aged TME (*N* = 1 measurement per patient for cytokine and estrogen measurements). **c**, Adding the conditioned media from P1 to P5 significantly increased CD206 and PD-L1 staining intensity on macrophages. ANOVA with correction for multiple comparisons used for statistical tests. Data shown as median with 95% CI. **d**, After macrophage polarization, we analyzed the media to measure what the macrophages were secreting. Heat map shows row-normalized values. TGF-β was significantly increased after macrophage polarization and persisted after a 24 h wash-out period. Friedman test with correction for multiple comparisons used for statistical tests given matched measures. **e**, Using the same experimental setup, we then treated macrophages with P5 conditioned media alone or in combination with carlumab (monoclonal antibody targeting CCL2) and/or fulvestrant (selective ER degrader). ANOVA with correction for multiple comparisons used for statistical tests. Data shown as median with 95% CI. **f**, Heat map showing inflammatory factors in the media after macrophage polarization or lack thereof in each of the conditions. **g**,**h**, Using the mIHC data from ref. ^35^, CD8^+^ and CD4^+^ T cells were lowest in the stromal regions from older patients with ER^+^/HER2^−^ breast cancer; these tumors had significantly lower neighborhood frequencies characterized by lymphocyte enrichment. *N* = 11 for Y, *N* = 39 for MA and *N* = 17 for O. Data shown as median with 95% CI. ANOVA with correction for multiple comparisons used for statistical tests. **i**, Colocalization analysis from spatial transcriptomics indicated that immunosuppressive macrophages had the highest colocalization with CD4 and CD8 T cells. Schematics in **a** and **b** created in BioRender; Carleton, N. https://biorender.com/xvczqdp (2026). Source data

To mechanistically examine and validate the degree to which the aged TME, which is characterized not only by a chronically inflamed TME but also by high local E2 levels, enhances macrophage polarization, we used macrophages differentiated from monocytes (isolated from human donor peripheral blood mononuclear cells (PBMCs)). To simulate the aged TME, we treated these macrophages with E2, CCL2, CXCL9 or their combination and quantified the staining intensity of the immunosuppressive markers CD206 and PD-L1. Treating with E2 alone significantly increased CD206 staining; the combination of all three agents promoted macrophage transition toward the CD206^+^/PD-L1^+^ state (Fig. 8a). Next, we used our prospectively collected panel of ER^+^/HER2^−^ PDOs from older patients (clinical characteristics detailed in Supplementary Table 4). To capture the secreted contents of all cells in the aged TME, we generated conditioned media from zero-passage, just-in-culture PDOs (*n* = 5; brightfield images of PDOs presented in Supplementary Fig. 7) and then incubated the conditioned media with monocyte-derived macrophages (Fig. 8b). We measured baseline levels of inflammatory cytokines and chemokines as well as estrogens present in the PDO conditioned media. CCL2 and IL-6 were the most abundant inflammatory cytokines, whereas E2 was nearly 10–20-fold higher than E1. The conditioned media significantly increased both CD206 and PD-L1 positivity in macrophages, further confirming that features of the aged TME promote the accumulation of immunosuppressive macrophages (Fig. 8c). Interestingly, when we again ran the same inflammatory panel on the media after the macrophages were polarized (containing secreted factors from the polarized macrophages), we observed a prominent secretory phenotype of IL-4, IL-10, IL-13 and TGF-β, functionally supporting the immunosuppressive nature of the macrophages and validating features of the macrophages observed in our spatial transcriptomic data (Fig. 8d). In particular, TGF-β significantly increased across the measured conditions (Fig. 8d).

Given the degree of secretory immunosuppression from the PDO conditioned media, which contained both high levels of inflammatory factors (the highest of which was CCL2 as demonstrated in the heat map in Fig. 8b) and high levels of E2, we tested a number of inhibitors for potential modulation of macrophage polarization. When we again treated the macrophages with conditioned media from P5, the macrophages again showed consistent polarization to a CD206^+^ state with increased secretion of CCL2, IL-4, IL-10, IL-13 and TGF-β (Fig. 8e,f). When we added either carlumab (anti-CCL2 monoclonal antibody) or fulvestrant (selective ER degrader), macrophage polarization was significantly decreased, although to a far greater degree with fulvestrant (Fig. 8e). Interestingly, however, it was the combination treatment that both reduced IL-4, IL-10, IL-13 and TGF-β secretion and restored anti-tumor inflammatory factor secretion.

Lastly, with the immunosuppressive nature of the macrophages, we examined levels of lymphocyte infiltration in the tumors from older patients. Pathway enrichment analysis showed significant decrements in T cell receptor signaling with age (Supplementary Fig. 8). CD8 and CD4 T-cell numbers similarly showed a trend toward decreased levels in our cohort of ER^+^/HER2^−^ patients (analyzed using multiplexed IHC; Fig. 8g). Compared with younger and middle-aged patients, older patients with ER^+^ tumors exhibited significantly lower levels of lymphocyte-enriched neighborhoods (Fig. 8h). Concordantly, in our spatial analysis, immunosuppressive macrophages had the highest degree of colocalization with CD4 and CD8 T cells (Fig. 8i).

**Extended Data Fig. 10:**
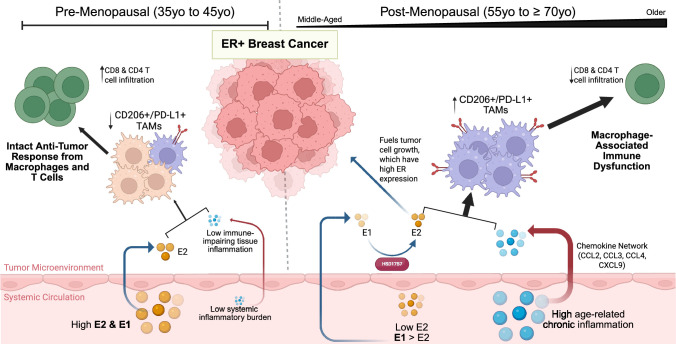
Schematic of the key findings of the study. Figure created in BioRender; Carleton, N. https://BioRender.com/fbmmjay (2026).

Overall, these data demonstrate that key components of the aged TME – the combination of high local E2 and chemokine-enriched inflammation – are sufficient to induce immunosuppressive macrophage accumulation and polarization with evidence of blunted anti-tumor T-cell immunity (Extended Data Fig. 10).

## Discussion

It is becoming increasingly recognized that ER^+^ breast cancer is an age-related disease, and owing to the substantial prevalence of this patient population in the clinic, increased attention to both differences in clinical management and tumor etiology and biology is required^39–41^. More so than younger patients, clinical management of older patients, typically defined as those older than 70 years, can be challenging in that tumor biology, life expectancy, competing risks of mortality, and patient preferences may all play a role in determining optimal therapies^39,40^. ER^+^ tumors in older patients tend to exhibit different clinical behaviors^42^, leading to recent changes in treatment paradigms in both the surgical setting^43,44^ and the adjuvant setting^45,46^. Despite this, our understanding of the underlying tumor biology, immune biology and role of the host environment and their contributions to tumor development, growth and ultimately behavior is limited.

The F344 rat model captures age-associated immune and inflammatory remodeling but does not fully recapitulate the E1-predominant endocrine milieu of women experiencing postmenopause. In aged rats, tumors arose in a context characterized by chemokine-enriched systemic inflammation and increased TAM features, rather than clear differences in mutational burden. Although tumors in older rats seemed more indolent despite immunosuppressive signals, a pattern also observed in human breast cancer, this may reflect differences between early immune escape and sustained tumor progression. It is possible that the suppressed immune environment plays a more prominent role in initial immune escape and less so with regard to sustained tumor progression^47,48^. Although this work implicates chronic inflammatory changes in the circulating environment and local immune contexture, tumor cell age-related changes certainly play a role in age-related tumorigenesis. Recent rodent work has explained potential tumor cell-driven changes, such as secretion of midkine, that reshape other mammary gland epithelial cells in the TME^49,50^. Together, these findings underscore the need to consider both host-derived inflammatory changes and tumor-intrinsic factors in understanding age-associated tumorigenesis.

Regarding hormone disposition, although it is paradoxical that so many ER^+^ breast cancers form following menopause, the source of local estrogens in postmenopausal breasts has been unresolved in the field for several decades^51^. However, because of the efficacy of aromatase inhibitors, interest in hormone disposition in the breast largely waned. Despite this, identification and characterization of the role of local hydroxysteroid dehydrogenase activity, specifically in the aged breast, along with a potential strategy for inhibition, may rekindle interest in an emerging therapeutic avenue.

Although older patients harbored a chronic inflammatory circulating environment with an E1-predominant estrogen disposition, the aged local TME was uniquely defined by high tumor ER levels *and* high levels of local E2. Why these tumors retain high levels of ER expression with mounting levels of E2 is unclear and warrants future exploration, given that in the postmenopausal noncancerous breast, low levels of E2 drive high levels of ER expression. An equally intriguing finding was the higher circulating E2 levels in patients with breast cancer versus the non-cancer donors. Because the blood was taken at the time of surgery, we did not know whether these patients had higher circulating E2 levels or were thus more likely to develop breast cancer, or whether this increase in E2 occurred after tumor formation (perhaps a spillover effect from the breast TME).

Nonetheless, the finding of HSD17B7-associated E2 accumulation in the aged TME is particularly engaging. Seminal studies several years ago suggested that local synthesis of E2 in the breast through aromatase activity was not the predominant modality driving intratumoral E2 levels^52–54^. It was shown instead that hydroxysteroid dehydrogenase activity was the single most important determinant of in-breast E2 levels^52^. Our work here suggests that for older patients, circulating E1 serves as an ‘estrogen pool’ that is readily converted by the HSD17B7 enzyme, which was the only dehydrogenase to show age-related expression increases specifically in tumor tissue. Resultingly, postmenopausal breast cancers have E2 levels similar to those in premenopausal breast cancers. Our findings here also minimize the potential role of local E1 activity in the setting of age-related breast cancer, given the decrements in *HSD17B2* expression and far higher E2:E1 ratios in the breast tissue, even in older patients^24,55^. Interestingly, preceding the increase in local E2 is the fact that the normal breast likely increases *ESR1* expression (and thereby increases ER expression) owing to the low circulating and local levels of E2 (refs. ^56,57^). As the breast tumor forms, increases in HSD17B7 expression create a local environment that not only already has high levels of ER but also has high levels of local E2 to serve as a proliferation amplifier, which may explain why these tumors are typically sensitive to endocrine therapy in early-stage disease settings. Even though aromatase inhibitor therapies provide sufficient coverage for preventing peripheral formation of both E1 and E2 from androgens, our study raises the possibility that local targeting of HSD17B7, a concept first proposed in 2011 (ref. ^51^), could potentially have a role in breast cancer therapy given the advent of newly synthesized inhibitors specific to the estrogenic role of these enzymes^34,35^. Our data on an array of PDOs generated directly from patients with ER^+^/HER2^−^ breast cancer show that this mechanism may be specific for older patients, as HSD17B7 inhibition had little activity in PDOs from younger patients. Given the systemic nature of aromatase inhibitors, side effects are numerous and often preclude adherence for the recommended 5 years. Local inhibition thus becomes attractive and may be achieved in a number of ways. Although our pharmacologic inhibition studies support a functional role for HSD17B7 in modulating local estrogen availability in ex vivo systems, genetic perturbation and in vivo validation are warranted to establish enzyme specificity and determine its necessity in tumor development or progression. Thus, HSD17B7 should be viewed as a promising candidate mediator of local estrogen metabolism.

An equally important finding of this work is the chemokine-enriched chronic inflammation that defined both the systemic and local environments. It is increasingly well established that older patients with breast cancer exhibit altered inflammatory profiles and immune changes with age^58–60^. However, understanding how systemic inflammatory changes drive local changes has been challenging. In this study, chemokines, or chemotactic molecules, especially CCL2, are associated with immunosuppressive macrophage accumulation in the aged breast TME, a finding corroborated by previous work in the setting of ER^+^ breast cancer^61^. We did not find local production of chemokines to play a prominent role in establishing an inflamed TME – gene expression of chemokines (and other cytokines) did not change with age across cell types when analyzed via scRNA-seq. This suggests that chronic inflammation of the host in the periphery likely shapes local tissue inflammation and the immune response in the local TME^62^.

Interestingly, although we found high levels of chemokines within the TME, indicating a chronically inflamed environment, we also observed broad immune dysfunction. This CCL2 axis may play an important role in establishing early immune escape for these early-stage ER^+^ tumors through orchestration of impaired macrophage–T cell communication^12,63,64^. Although total macrophage levels did not change across age groups, we show that CD206^+^ macrophages, known for their pro-tumor functions, were highest in the aged TME, in line with previous studies in the mouse mammary gland^65^. Targeting CCL2 not only reduced macrophage polarization to a CD206^+^/PD-L1^+^ state but also reduced pro-tumor, immunosuppressive factors such as TGF-β. These findings raise the idea that targeting CCL2 not only may work to treat the host environment, thereby reducing systemic chronic inflammation, but also may serve to re-establish an intact anti-tumor immune response locally. In the setting of ER^+^ breast cancer, potential immune induction approaches targeting CCL2 may be a worthwhile and feasible strategy, either alone or in combination with endocrine therapy; data from this study suggest at least some synergism when targeting both CCL2 and ER, which not only reduce macrophage polarization but also restore the balance between anti- and pro-tumor cytokines that communicate with adaptive immune cells. An important implication of the work described here is the increasingly recognized idea that macrophages (and potentially other immune cells) are sensitive to estrogens and endocrine therapies^66,67^. Here we show that macrophages may serve as ‘hubs’ of integration, integrating the unique signals of the TME, such as chronic inflammation (especially the chemokines), and the high local E2 levels. Indeed, macrophages seem to not only be sensitive to E2 but also be polarized toward a CD206^+^ state when treated with E2 alone. Ongoing observational trials seek to better understand the role that endocrine therapies play in modulating systemic and local immunity^68^ and will be important for better understanding how macrophages may aid anti-tumor responses after endocrine therapy. We recognize that macrophage biology within human tumors is highly heterogeneous and cannot be fully captured by simplified polarization frameworks^36,69^. Rather than representing discrete binary states, TAMs likely exist along a continuum of activation programs shaped by local cytokine, hormonal and metabolic cues. Our analyses identify enrichment of immunosuppressive transcriptional features and surface markers consistent with macrophage-mediated immune modulation but do not resolve specific lineage trajectories or functional subsets. Future single-cell lineage tracing and in vivo perturbation studies will be required to more precisely define macrophage states in aging-associated tumors.

This study offers insights into the systemic and local factors contributing to ER^+^/HER2^−^ breast cancer in older women, but several limitations should be noted. Although our human cohorts included matched blood and tissue samples, they were observational and cross-sectional in nature, and the analysis was largely correlation based; therefore, we cannot assign causality or fully capture dynamic longitudinal changes in the TME. Our analyses of circulating inflammatory markers are associative; however, our functional studies demonstrate that chemokines and E2 are sufficient to induce macrophage polarization, providing mechanistic insight. Although we integrated bulk and single-cell transcriptomic data with molecular and functional validation using PDOs, the number of PDOs used was limited, particularly in comparing aged and non-aged contexts. We identified potential therapeutic avenues (for example, HSD17B7 and CCL2 inhibition), but these findings require further in vivo validation and prospective clinical evaluation to determine translational potential. CCL2 emerged as the most consistently age-associated chemokine across cohorts; however, other chemokines may contribute cooperatively to shaping the aged TME. Furthermore, a more detailed analysis of other immune effectors within the TME, particularly T cells, is warranted. Although we observed reduced TCR signaling pathway enrichment and spatial proximity between immunosuppressive macrophages and T cells, direct functional assays of T-cell cytokine production were not performed and remain an important area for future investigation.

In summary, the work presented here provides insights into the unique systemic and local hormonal and inflammatory milieu that creates a permissive environment for ER^+^ breast cancer development and growth. This study uncovers a number of therapeutic avenues, such as targeting local HSD17B7 enzymes or CCL2 to dampen the immunosuppressive features of the aged environment, that will require in vivo and early-phase clinical trials for evaluation. These data provide a strong rationale for age-specific therapeutic modalities for future in vivo studies and human trials.

## Methods

### Ethical declarations

All studies with human tissue were conducted in accordance with protocols approved by the Institutional Review Boards of the University of Pittsburgh (STUDY20040297 and STUDY21100091) and the University of Pittsburgh Medical Center (IRB0502025/HCC protocol 04-162). Written informed consent from each patient or donor was obtained before tissue collection and banking. Some samples used in this study were prospectively collected from a clinical trial^38^ (NCT05914792). To assemble an age-matched control cohort of women without clinical breast disease, we obtained fresh-frozen normal breast tissue cores and matched serum from the Susan G. Komen Tissue Bank at the IU Simon Comprehensive Cancer Center. At the time of donation, participants provided written informed consent and were enrolled under a protocol approved by the Institutional Review Board at Indiana University. Written informed consent from each patient or donor was obtained before tissue collection and banking. All animal (rat) experiments were performed following protocols 20108061 and 23103979, which were both approved by the University of Pittsburgh Institutional Animal Care and Use Committee.

### Rat experiments

All animals were housed in the Ford/Assembly Research Building at the University of Pittsburgh in an approved animal facility. All animal experiments were performed following protocols 20108061 and 23103979, which were both approved by the University of Pittsburgh Institutional Animal Care and Use Committee. Virgin F344 rats aged 6–8 weeks were purchased from Envigo and served as the ‘young’ rats in the study. Aged F344 rats were obtained from the National Institute on Aging Rodent Colonies at 19–21 months old and served as the ‘older’ rats in the study. All rats were housed in ventilated autoclaved cages (Allentown Caging) on hardwood chip bedding, had 12 h/12 h light–dark cycles, were provided with hyperchlorinated reverse osmosis water and were fed ad libitum rodent chow (Purina). Rats lived in the facility for at least 2 months before any experimental treatment to allow for equilibration.

To better understand the ovulatory status of the rats in each age group, vaginal cytology was performed on a cohort of rats not treated with DMBA or MPA and was performed for 14 consecutive days for 6 young and 6 older rats using a protocol that evaluates neutrophils and the size and nuclei of shed epithelial cells^70^. Cytology specimens were dried overnight and stained using the Diff-Quik staining protocol.

### DMBA/MPA model

To investigate the effect of age on tumor incidence and growth, we used a chemical carcinogen model. All rats were surgically implanted with a 50 mg MPA pellet in a subcutaneous fashion (90-day continuous release; Innovative Research of America, #NP-161). One week following pellet implant, rats were dosed with a single oral gavage of DMBA (65 mg/kg; Thermo Scientific Chemicals, #408181000) dissolved in sesame oil (Thermo Scientific Chemicals, #241002500). All rats recovered fully from pellet implant surgery and tolerated the DMBA dose.

### Tumor growth analysis and tissue collection

Mammary tumor development and growth were monitored biweekly by palpating all six mammary gland pairs. Once a tumor formed, we assessed growth dynamics using a volumetric calculation of tumor growth ((*L* × *W* × *W*)/2). The experimental endpoint was reached once the tumor reached 3,000 mm^3^ (maximum allowed under Institutional Animal Care and Use Committee protocol) or when the rat showed signs of clinical decline. No tumors exceeded the maximum allowable volume. Tumors were collected and stored as cryopreserved specimens, flash frozen or further processed for paraffin embedding (10% formalin overnight, stored thereafter in 70% ethanol). All tumors were stained for ER (abcam, #ab32063, 1:10,000 dilution) and Ki-67 (Novus Biologicals, NB600-1252, 1:100 dilution).

All tumors were histologically confirmed by an experienced animal pathologist. All subsequent grading (mitoses per HPF, percentage of tumor cells positive for ER, percentage of tumor cells positive for Ki-67 and stromal tumor infiltrating lymphocytes) was performed in a blinded fashion with respect to the age of the animal and was calculated as the average of four different areas of each tumor specimen.

### Rat tumor sequencing and computational analysis

A group of six flash-frozen tumors (all three tumors from younger rats and three tumors with adequate tissue from older rats) was processed and subjected to snRNA-seq, low-pass whole-genome sequencing, whole-exome sequencing and bulk RNA-seq.

### Patient cohorts and clinical specimens

#### Patients with ER^+^/HER2^−^ breast cancer from PBC

To assemble a cohort of patients with ER^+^/HER2^−^ breast cancer across age groups, we obtained archived specimens from patients who had matched tumor tissue, tumor-adjacent tissue and blood from the University of Pittsburgh’s PBC. No patients included in this cohort received neoadjuvant systemic therapy and were considered treatment naive at the time of tissue and blood collection. Tumor-adjacent tissue was taken approximately 3–4 mm away from the tumor in lumpectomy cases and 5–6 mm away from the tumor in mastectomy cases. All patients signed informed consent forms for their specimens to be used in research applications. We collected patient and tumor characteristics associated with the specimens. All patients were diagnosed with ER^+^/HER2^−^ early-stage breast cancer. We obtained specimens from the young (35–45 years old), middle-aged (55–69 years old) and older (≥70 years old) groups. We intentionally excluded the perimenopausal group, as estrogen levels change variably across these ages, and we wanted to have a stronger comparison between the three age groups without potential confounding from the perimenopausal group.

We used the standard procedure of the RNeasy kit to extract total RNA from ER^+^ breast cancer samples of 83 tumors or 85 tumor-adjacent normal tissues obtained from the PBC and grouped the samples by chronological age. The NovaSeq 6000 library for DNA sequencing was prepared using the TruSeq Stranded mRNA Library Prep Kit (Illumina) following the protocol provided by the manufacturer. Library size and quality were checked using a Fragment Analyzer (Agilent) and fluorescent quantification on the Infinite F Nano+ (Tecan). The libraries were normalized and pooled and then sequenced using the NovaSeq 6000 platform (Illumina) to generate an average of 25M 101-bp paired-end reads. Sequencing data were given as raw data with a Phred Q30 score of 80 or better. For processing, we used Rsubread^71^ (Bioconductor release 2.16) to align sequence reads to the reference genome and used the edgeR^72^ and limma^73^ R packages (Bioconductor release 4.0 and 3.58, respectively) to normalize gene expression levels to log2 transcripts per million^74^. We aligned sequence reads to the GRCh38 release 14 human genome reference sequence and mapped the aligned sequences to Entrez Gene. After normalization, we removed genes with zero expression across all samples, resulting in 39,404 genes for further pathway analysis.

Our investigation into pathway activities involved the use of two distinct R packages, PROGENy^75^ (version 1.22) and GSVA^76^ (version 1.48.3), with default parameters. PROGENy is a resource that leverages a large compilation of signaling perturbation experiments for a common core of pathway-responsive genes. GSVA provides heightened sensitivity for detecting subtle changes in pathway activity within a sample population. In our GSVA analysis, we used gene set collections associated with the estrogen pathway. These gene sets were sourced from the HALLMARK, Reactome, WikiPathways and Gene Ontology biological pathways. These two tools allowed us to infer the activities of biological pathways. We calculated Spearman’s correlation coefficients between the inferred pathway activities in a single sample and the expression of genes involved in estrogen conversion.

### Specimens from donors participating in the Komen Tissue Bank

Details of the Komen Tissue Bank project (http://komentissuebank.iu.edu/) have been previously described^77,78^. We obtained specimens from the young (35–45 years old), middle-aged (55–69 years old) and older (≥70 years old) groups. Relevant exposures, such as age at menarche and hormone replacement therapy use, are recorded in Supplementary Table 2.

#### Spatial transcriptomics and proteomics from a cohort of older patients with ER^+^/HER2^−^ breast cancer

Spatial transcriptomic profiling was performed on six diagnostic core biopsy specimens. In brief, formalin-fixed paraffin-embedded tissue sections were processed using the 10x Genomics Visium CytAssist platform with the FFPE v2 Gene Expression workflow and the Human Immune Cell Profiling Panel, enabling spatially resolved transcriptomic and proteomic measurement while preserving tissue architecture. Libraries were generated according to the manufacturer’s protocol, quality controlled and sequenced on the Illumina NextSeq 2000 platform. Spatial gene expression data were processed using established preprocessing and normalization pipelines, followed by cell-type deconvolution and spatial analysis using the CITEgeist framework^79^. These spatially resolved transcriptomic data were subsequently used for downstream analyses of tumor and immune cell states and their spatial interactions.

### Quantification of inflammatory factors

To analyze the inflammatory burden in the human serum, tissue microenvironment, rat serum and PDO conditioned media, we used the Luminex multiplex system. In brief, beads of defined spectral properties conjugated to analyte-specific capture antibodies and samples (including standards of known analyte concentration, control specimens and unknowns) were pipetted into the wells of a filter-bottom microplate and incubated. During the first incubation, analytes bound to the capture antibodies on the beads. After washing the beads, analyte-specific biotinylated detector antibodies were added and incubated with the beads. During the second incubation, the analyte-specific biotinylated detector antibodies recognized their epitopes and bound to the appropriate immobilized analytes. After the incubation of biotinylated detector antibodies, streptavidin conjugated to the fluorescent protein, R-phycoerythrin (streptavidin–R-phycoerythrin), was added (without washing) and incubated. During the final incubation, streptavidin–R-phycoerythrin bound to the biotinylated detector antibodies associated with the immune complexes on the beads, forming a four-member solid-phase sandwich. After washing to remove unbound streptavidin–R-phycoerythrin and biotinylated detector antibodies, the beads were analyzed using the Bio-Plex 200 instrument with the Bio-Plex Manager 6.2 software.

For all human studies (including human serum and tissue lysates), we ran a 22-plex panel consisting of GM-CSF, CXCL1, IFN-γ, IL-1β, IL-6, IL-8, IL-10, IL-17A, CCL2, CCL3, CCL4, TNF-α, G-CSF, M-CSF, IL-1α, IL-2, IL-5, IL-13, IL-15, IL-18, CXCL10 and CXCL9. For all rat serum studies, we ran an 18-plex panel consisting of IFN-γ, IL-1β, CXCL1, IL-6, IL-10, IL-17, G-CSF, IL-18, IL-5, CCL3, IL-1α, CCL2, IL-2, CXCL10, IL-13, GM-CSF, TNF-α and IL-15. Lastly, for the human PDO conditioned medium analysis, we ran a 15-plex consisting of CCL2, CCL3, CCL4, CXCL1, CXCL9, CXCL10, TNF-α, IL-6, IL-12, IL-23, IL-4, IL-10, IL-13 and TGF-β. Calculation of the composite circulating chemokine score was performed to determine the collective burden of circulating CCL2, CCL3, CCL4 and CXCL9. It was calculated as the number of markers that were two standard deviations above the marker mean for each patient.

### Mass spectrometry for estrogen (E1 and E2) measurements

A previously validated liquid chromatography-tandem mass spectrometry (LC-MS/MS) assay for the quantification of E2 and E1 in human serum was adapted and used for this study^80^. In brief, an LC-MS/MS system consisting of a Thermo Scientific Vanquish UPLC and TSQ Altis that was equipped with a heated electrospray ionization source was used, whereas the selected reaction monitoring transitions used for quantitation were m/z 504.3 → 171.1 for E1 and m/z 506.2 → 171.1 for E2. The mixture, comprising 500 µl of serum and 25 µl of internal standard, was subjected to liquid–liquid extraction with 3 ml of *N*-butyl chloride. The sample was briefly vortexed and centrifuged at 3,000 × *g* for 5 min before the organic layer was transferred and evaporated under nitrogen. The samples were derivatized using 50 µl of 50 mM sodium bicarbonate buffer in addition to 50 µl of dansyl chloride (1 mg ml^−1^) and were then heated for 3 min at 60 °C. The samples were transferred to LC vials, and 7.5 µl was injected into the LC-MS system. Chromatographic separation of the samples was accomplished using a Waters ACQUITY BEH C18 (2.1 × 150 mm, 1.7 µm) column with a gradient elution of 0.1% formic acid (A) and acetonitrile (B) at a flow rate of 0.3 ml min^−1^. The total runtime was 6.5 min.

### Publicly available datasets used

#### scRNA-seq of treatment-naive ER^+^/HER2^−^ breast cancer

We compiled scRNA-seq datasets from ten ER^+^ breast tumor samples, focusing exclusively on cells from pre-treatment patients. The processed scRNA-seq data, originally published by Wu et al.^27^, were obtained from the NCBI Gene Expression Omnibus under accession number GSE176078. To analyze the data, we imported the matrix.mtx, features.tsv and barcodes.tsv files into a Seurat object using the Read10X function in the Seurat R package^81^ (version 5.0.3). Our cell selection criteria included an expression of fewer than 6,000 genes, with a minimum of 200 expressed genes and 400 unique molecular identifier counts. In addition, we filtered out cells with more than 15% of reads mapped to mitochondrial gene expression. To address technical variations and batch effects, we applied SCTransform to each Seurat object^82^. SCTransform is a technique designed to mitigate technical variations and alleviate batch effects in scRNA-seq datasets. Principal component analysis was performed on the filtered feature-by-barcode matrix, and Uniform Manifold Approximation and Projection embeddings^83^ were based on the first 30 principal components. Overall, our processed dataset comprised 29,733 genes across 30,959 cells. For cell-type annotation, we followed the major and subset cell types provided by Wu et al.

We used CellPhoneDB^84^ (version 4) to investigate potential ligand–receptor interactions between macrophages and other immune cells. Specifically, we analyzed raw count matrices separately for the younger and older groups. For each group, we calculated the mean average expression levels of interacting ligands in the sender population and interacting receptors in the receiver population. To assess the statistical significance of each interaction score, we performed a one-sided Wilcoxon signed-rank test.

### Bulk RNA-seq datasets: TCGA, METABRIC and SCAN-B

We used the TCGA^85^, METABRIC^86^ and SCAN-B^87,88^ datasets for bulk RNA-seq analysis. Within each dataset, we only selected ER^+^/HER2^−^ tumors within the designated age groups defined in this study. To identify genes and pathways with consistent expression or enrichment patterns across various age groups in the TCGA, METABRIC and SCAN-B datasets, we used the MICA framework^26^. MICA is a statistical framework designed to identify gene expression trends that are consistently associated with a variable of interest (in this case, age) across independent datasets. Rather than relying on a single cohort, MICA prioritizes genes that demonstrate concordant directional changes across multiple studies, thereby increasing robustness and reducing the likelihood of cohort-specific artifacts. Initially, it performs a global test to evaluate overall concordance, followed by a post-hoc analysis to pinpoint specific studies contributing to this concordance. This approach ensures robust detection of biomarkers exhibiting consistent expression across multiple datasets and was validated alongside conventional statistics.

#### mIHC cohort of ER^+^/HER2^−^ IDC and invasive lobular carcinoma

To examine changes in immune cells and cellular neighborhoods across age groups, we used a previously developed cohort of patients with ER^+^/HER2^−^ IDC and invasive lobular carcinoma^37^. We reanalyzed the data according to the age groups defined in this study; further details on antibodies and staining, image acquisition and cell quantification, and cellular neighborhood analysis can be accessed through the work by Onkar and colleagues^37^.

### PDO generation, culture and conditioned media

PDOs were prospectively collected from consecutive patients older than 70 years with ER^+^/HER2^−^ tumors who signed consent forms for specimens to be included in research. Primary human breast cancer tissue was obtained by the PBC from patients who consented under the Breast Disease Research Repository: Tissue and Bodily Fluid and Medical Information Acquisition Protocol (HCC 04-162). PDOs were generated from this tissue in accordance with Institutional Review Board STUDY22030183 by the Institute for Precision Medicine according to established protocol^89^. Breast organoid media consisted of Advanced DMEM:F12 (+1× Glutamax, 10 mM HEPES and 1× antimicrobial/antimycotic), 1 nM β-estradiol, 500 nM A83-01, 5 ng ml^−1^ EGF, 250 ng ml^−1^ R-Spondin, 100 ng ml^−1^ Noggin, 5 nM Heregulin β-1, 5 ng ml^−1^ FGF7, 20 ng ml^−1^ FGF10, 500 nM SB-202190, 5 µM Y-27632, 1.25 mM *N*-acetyl-l-cysteine, 5 mM nicotinamide, 1× B27 supplement and 50 µg ml^−1^ Primocin. In brief, tumors were digested with 2 mg ml^−1^ collagenase (Sigma C9407) on a rotator, sheared, filtered at 100 µm and embedded in 40 µl domes of Cultrex growth factor reduced basement membrane extract type 2 (Fisher 35-330-1002) in 24-well non-treated plates (Fisher 12-566-82). At passage 0, as soon as the organoids were established in culture, 600 µl breast organoid medium was added to each well, and conditioned medium from organoids was collected after 2–3 days.

### PDO and cell line experiments testing for conversion from E1 to E2

PDOs from older patients with ER^+^/HER2^−^ breast cancer, from a younger patient with ER^+^/HER2^−^ breast cancer experiencing perimenopause and from an older patient with TNBC were seeded approximately 10 days before experiment commencement. We noted that all PDOs were collected from patients who did not receive any neoadjuvant systemic therapy. Twenty-four hours before treatment with E1, a wash-out of the PDOs was performed, and breast organoid media without E2 were added to the culture. On the first day of the experiment, PDOs were then treated in duplicate with 1.5 nM E1 without E2 for 24 h. After 24 h, the media were collected and underwent mass spectrometry analysis as described above. We subsequently used the same system to test two inhibitors to reduce E1-to-E2 conversion, including compound 10 (HSD17B7 inhibitor, treatment at 1 µM for 24 h)^33^ and CC-156 (HSD17B1 inhibitor, treatment at 1 µM for 24 h)^90^. MCF7, T47D and MM231 (source ATCC, authentication performed by STR profiling) cell lines were also used in preliminary experiments to test conversion.

### scRNA-seq of a PDO from an older patient after estrogen and anti-estrogen treatments

A PDO established from an ER^+^/HER2^−^ breast tumor from an older patient (P5) was used for scRNA-seq following hormonal manipulation. Before treatment, organoids were cultured in estrogen-free breast organoid media for 3 days. Organoids were then treated for 24 h under the following conditions: vehicle control, E1 (1.5 nM), E2 (1.5 nM), E1 (1.5 nM) plus HSD17B7 inhibitor (4 µM), E2 (1.5 nM) plus HSD17B7 inhibitor (4 µM), E1 (1.5 nM) plus fulvestrant (1 µM) and E2 (1.5 nM) plus fulvestrant (1 µM). Following treatment, organoids were dissociated into single-cell suspensions and processed using 10x Chromium Flex with a custom GRCh38-2024-A reference genome in which probe filtering was disabled (filter-probes=false) to retain HSD17B7, the drug target absent from the default v1.1.0 probe set. After Scrublet doublet detection (expected rate 0.06) and quality control filtering (≥500 genes, ≥1,000 UMIs and <15% mitochondrial), cells were normalized (counts per million, target sum 10,000; log1p), and 3,000 highly variable genes were selected (seurat_v3 method) and clustered using the Leiden algorithm (resolution 0.5) on 30 principal components. Pathway activity was inferred using GSEAPreranked (MSigDB Hallmark, 1,000 permutations, seed 42), PROGENy (top 300 footprints) and DoRothEA (levels A/B/C) via decoupler. Eight custom gene sets and four ER subprograms were scored at single-cell resolution (sc.tl.score_genes) with the Mann–Whitney *U* test, the two-sample Kolmogorov–Smirnov test, Cohen’s *d* effect sizes and the Benjamini-Hochberg false discovery rate correction. Cell cycle phase distributions were compared across treatments using chi-square tests. Proliferating cells were classified using a two-component Gaussian mixture model on proliferation scores. HSD17B7 inhibitor mechanism was investigated through E1/E2-dominant gene signature classification and projection analysis. Intra-treatment heterogeneity was assessed via neighborhood mixing entropy, silhouette scores and an estrogen response continuum analysis. All stochastic analyses used seed 42.

### Macrophage polarization experiments

PBMCs were isolated from healthy donors using Ficoll (Cytiva, #17-5446-52) and SepMate-50 tubes (STEMCELL, #85460) for density gradient centrifugation according to the manufacturer’s procedure. Monocytes were isolated from PBMCs (pan monocyte isolation kit Miltenyi, #130-096-537) before seeding in a 96-well plate and differentiation to macrophages with 5 ng ml^−1^ human M-CSF for 2 days. PDO conditioned medium was collected and diluted at a 1:1 ratio with fresh Roswell Park Memorial Institute 1640 medium. After washing out the M-CSF with phosphate buffered saline, diluted PDO conditioned medium was added to the monocyte-derived macrophages for polarization. After 3 days, samples were fixed with 4% paraformaldehyde, permeabilized with 0.1% Triton X-100 and blocked for immunofluorescent staining. Immunofluorescent staining images were analyzed using CellProfiler^91^ to determine the mean intensity of macrophage polarization markers of each cell. We quantified the expression levels of CD206 (BioLegend, #321102) and PD-L1 (BioLegend, #329701) in macrophages. To test whether we could block macrophage polarization, we added carlumab (an anti-CCL2 antibody; MedChemExpress HY-P99188), fulvestrant (a selective ER degrader; ICI 182780) and/or an IgG control antibody to the macrophage–PDO conditioned medium system.

### Statistics and reproducibility

To assess differences in baseline characteristics in the human tissue cohorts, Fisher’s exact test was performed for categorical variables and the Mann–Whitney test was performed for continuous variables. Correlation analyses throughout the paper were assessed using a nonparametric Spearman’s rank test. A multivariable linear model was used to test the association between plasma and tissue inflammatory factors and chronological age. The two-tailed Mann–Whitney test was performed for all pairwise comparisons; ANOVA with a correction for multiple comparisons was performed for all other multigroup comparisons. Data distribution was assumed to be normal, but this was not formally tested.

Studies using archived frozen and formalin-fixed paraffin-embedded human specimens were coded, processed and analyzed in a blinded fashion, whereas studies using prospectively collected PDOs were not. However, endpoints were quantified using objective imaging-based measurements (such as CellProfiler) to limit bias. No statistical method was used to predetermine sample size; the sample size was determined by the number of available specimens with matched blood, tumor tissue and tumor-adjacent tissue in our institutional biospecimen repository. All available biospecimens were used. In some cases, we had very limited tissue and prioritized estrogen analysis over inflammatory analysis; thus, sample sizes may not be equal across all analyses. Analyses were conducted using complete-case data for each variable of interest. No imputation was performed. Inclusion criteria for these biospecimens were as follows: age within the bounds of the prespecified younger, middle-aged and older categories; and early-stage (stage I through III; excluded patients with de novo stage IV disease and those with stage 0 ductal carcinoma in situ) invasive breast cancer, any histology permitted. In the rat studies, power calculations to determine sample size were based on differences in expected tumor incidence rates in the young and older rat groups. Blind independent review of tumor histology, ER staining and Ki-67 was performed by a trained animal pathologist; a minimum of four tumor areas were assessed.

The experiments were not randomized. Human participants were not compensated for their biospecimens. Any sample exclusion is as described above or in the figure legend. All in vitro studies were replicated a minimum of three times as described in the figure legends.

### Reporting summary

Further information on research design is available in the Nature Portfolio Reporting Summary linked to this article.

## Supplementary information

Supplementary Information Supplementary Figs. 1–8, Supplementary Tables 1–4, Supplementary Methods.

Reporting Summary

Peer Review File

## Source data

Source Data Single file containing all source data with clearly named tabs for each figure and extended data figure. This file includes tabs for Figs. 1–8 and Extended Data Figs. 1, 3, 6, 7 and 9.

## Data Availability

Source data are provided as Supplementary Information. All raw genomic data were deposited to Gene Expression Omnibus under the following accession numbers: GSE276755 (human tumor and tumor-adjacent bulk RNA-seq), GSE276757 (rat tumor bulk RNA-seq), GSE276759 (rat tumor whole-exome sequencing), GSE276758 (rat tumor snRNA-seq) and GSE325982 (scRNA-seq of PDO). Proteomic data derived from the Luminex platform quantifying inflammatory panels are deposited to figshare and available for download at 10.6084/m9.figshare.32620215 (ref. ^92^).

## Code availability

The code can be accessed via GitHub at https://github.com/leeoesterreich/ERpBRCA_OlderWomen.
