# Supplementary material for: Systemic and local chronic inflammation and hormone disposition promote a tumor-permissive environment for breast cancer in older women
Source: Nat Aging. 2026 Jul 27;6(8):1695–716. doi: 10.1038/s43587-026-01173-4 (PMC13472878; doi:10.1038/s43587-026-01173-4)
Supplement: Supplementary file 1 [file 43587_2026_1173_MOESM1_ESM.pdf]

nature aging

Supplementary information https://doi.org/10.1038/s43587-026-01173-4

Systemic and local chronic inflammation and hormone disposition promote a tumor-permissive environment for breast cancer in older women

In the format provided by the authors and unedited

Supplementary Information

Systemic and local chronic inflammation and hormone disposition promote a tumor-permissive environment for breast cancer in older women

This file includes:

Supplementary Figures 1 through 8
Supplementary Tables 1 through 4
Supplementary Methods

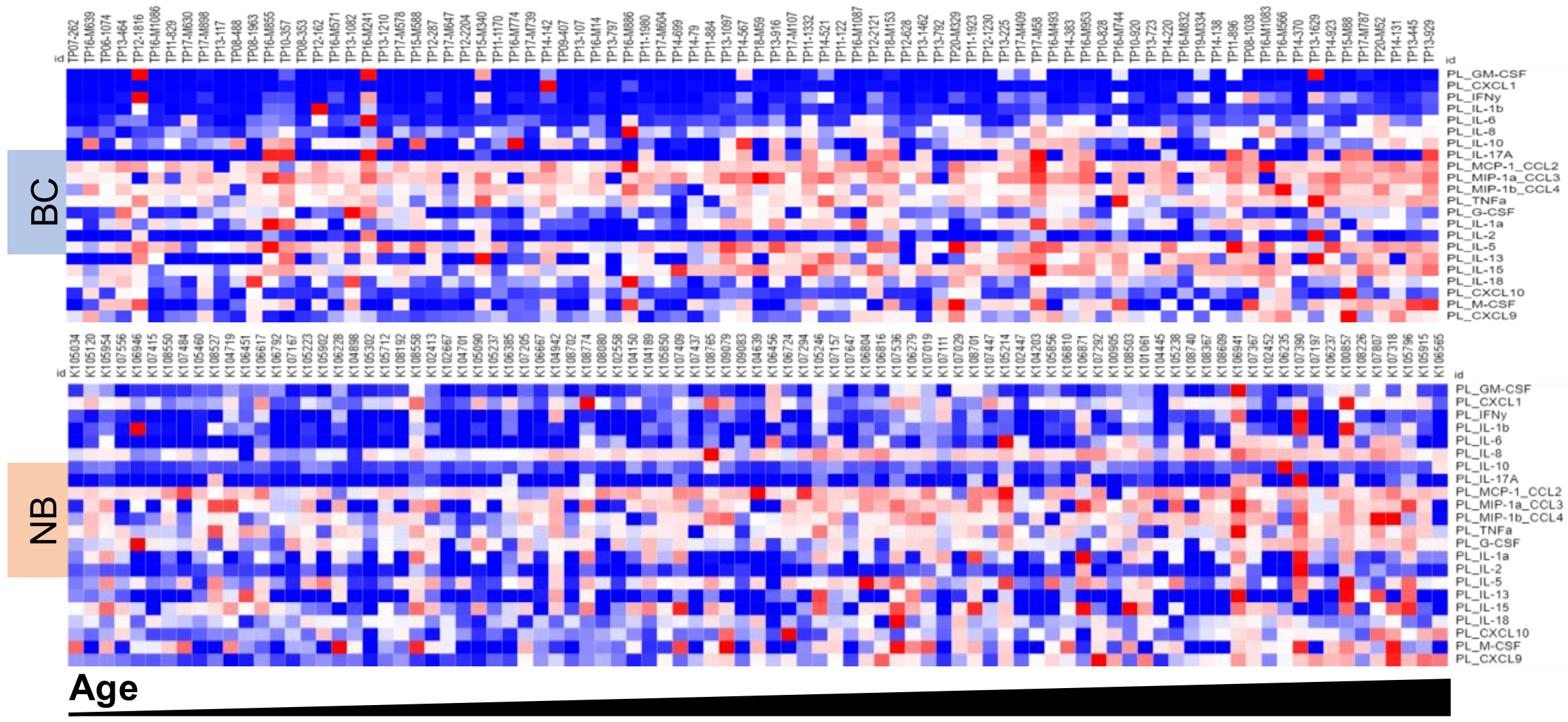

Supplementary Fig. 1: Heatmaps displaying the circulating inflammatory environment. Heatmap shows all patient (with ER+ breast cancer, "BC") and all donor (from the Komen Tissue Bank, "NB") plasma analyzed using the 22-marker Luminex panel. Patients and donors are arranged according to chronological age. N = 115 patients with BC and N = 89 donors.

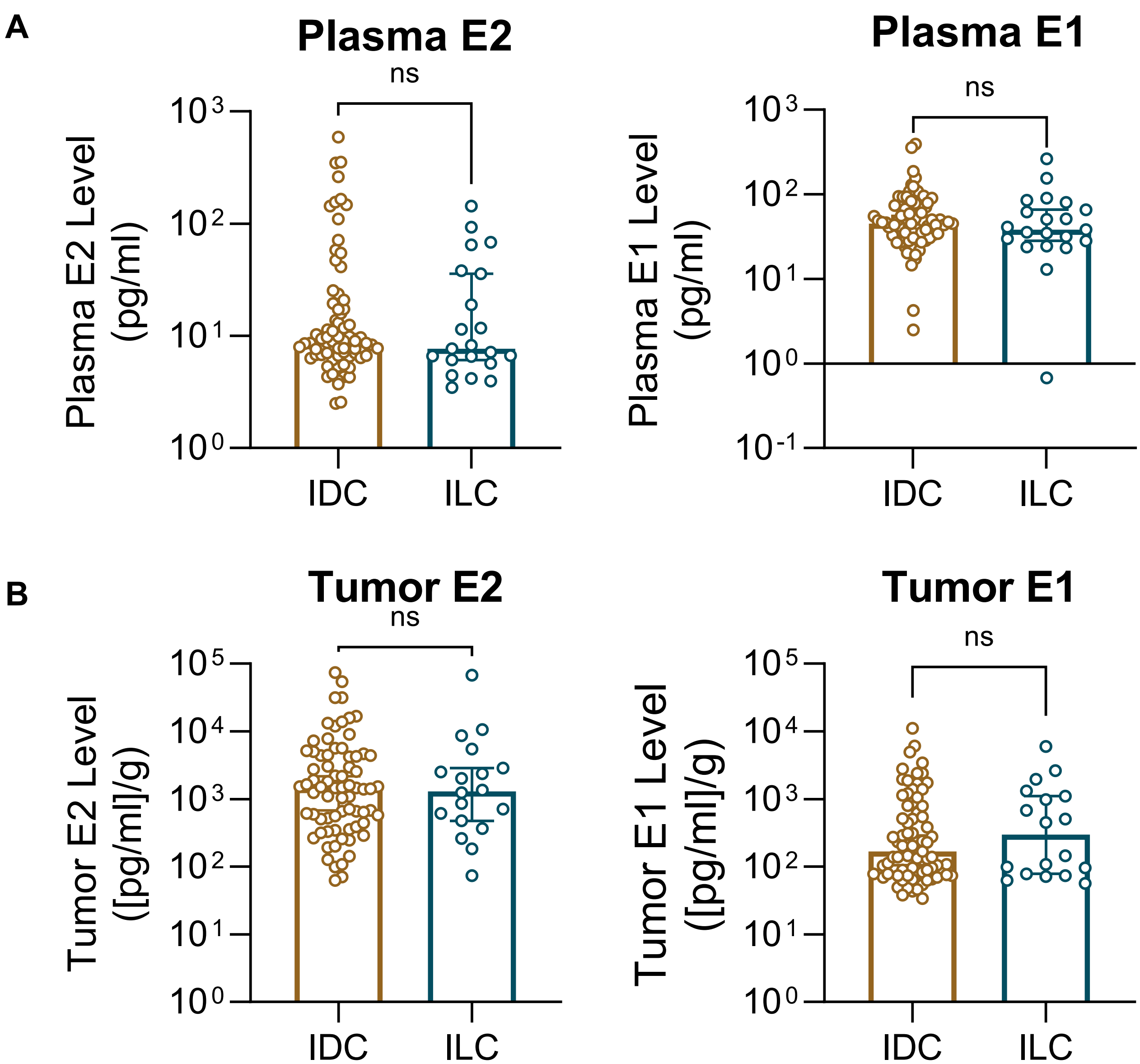

Supplementary Fig. 2: Association between estrogen levels and tumor histology. (A and B) Plasma E1 and E2 and tumor E1 and E2 were not significantly different between patients with IDC and ILC. N = 115 patients.

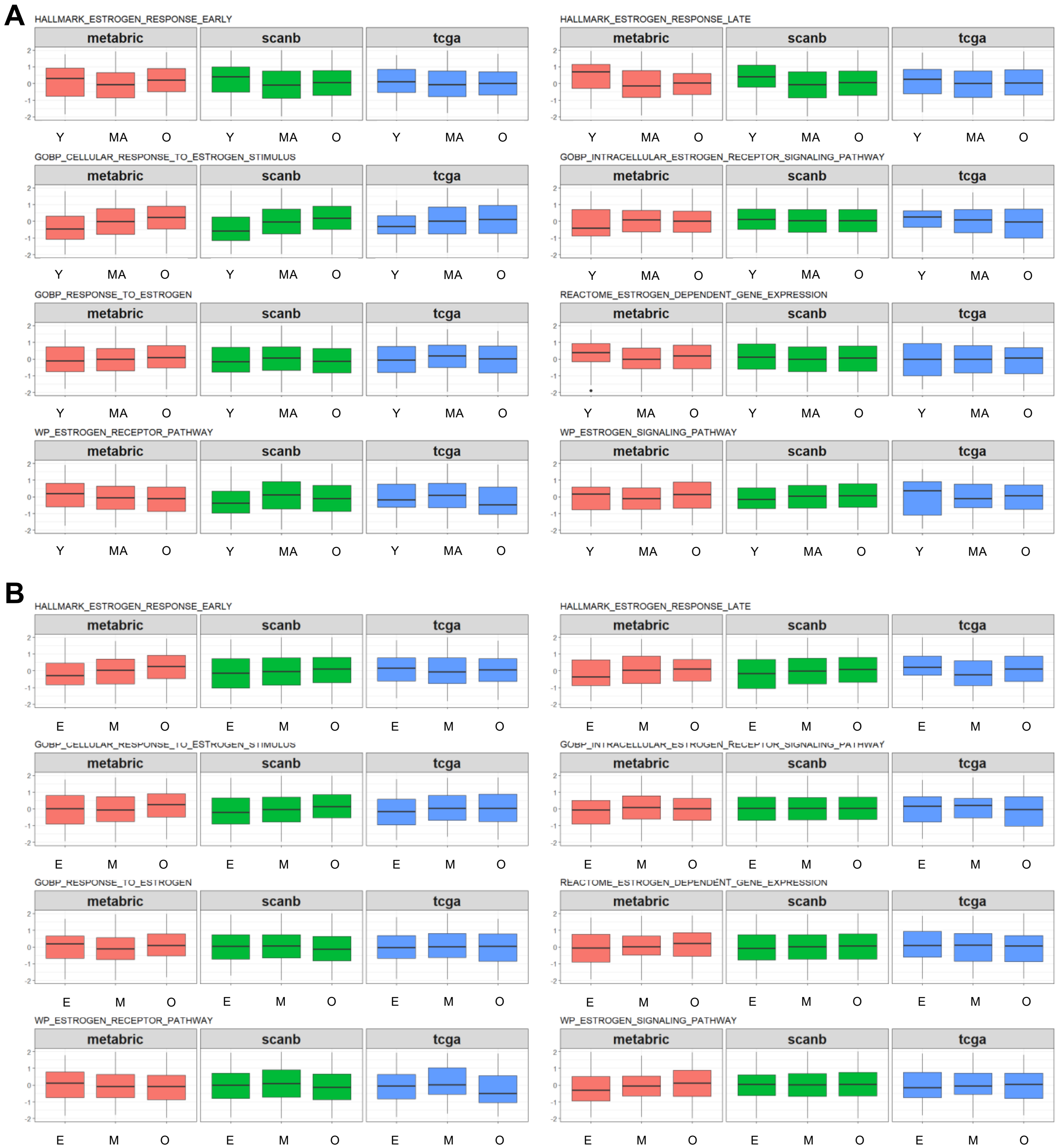

Supplementary Fig. 3: A panel of estrogen-related pathways analyzed through the mutual information concordance analysis (MICA) pipeline. (A) This analysis used the standard age cutoffs (Young, 35-45yo; Middle-Aged, 55-69yo; and Older, ≥ 70yo) to examine changes in estrogen-pathway activation across age groups. There were no significant changes across ages in any of the analyzed pathways. (B) This analysis only included post-menopausal patients and again found no significant differences in changes in estrogen-related pathways across ages (Early PostM ”E”, 55-60yo; Middle PostM “M”, 61-70; Older “O”, ≥ 70yo).

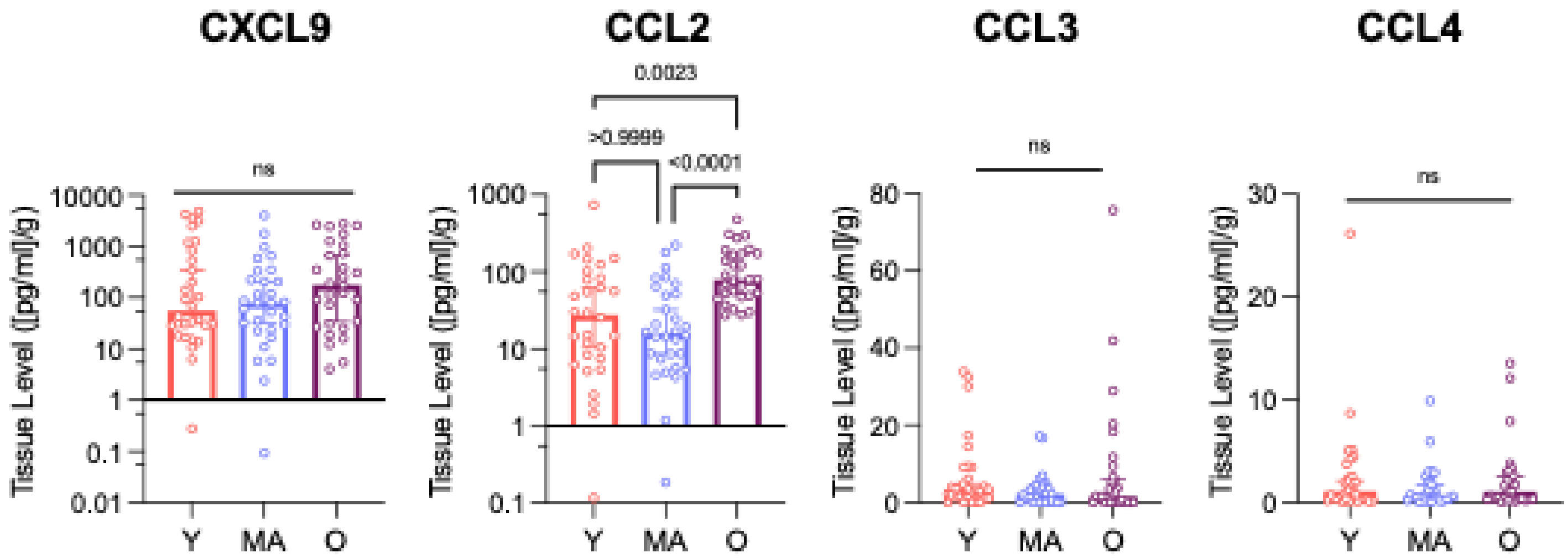

Supplementary Fig. 4: Chemokine levels across age from donors of normal breast tissue. Data shows measured CXCL9, CCL2, CCL3, and CCL4 across age as measured from the tissue lysates from normal breast tissue from donors of the Komen Tissue Bank. N = 25 for Y, N = 57 for MA, and N = 37 for O. ANOVA corrected for multiple comparisons used for statistical testing.

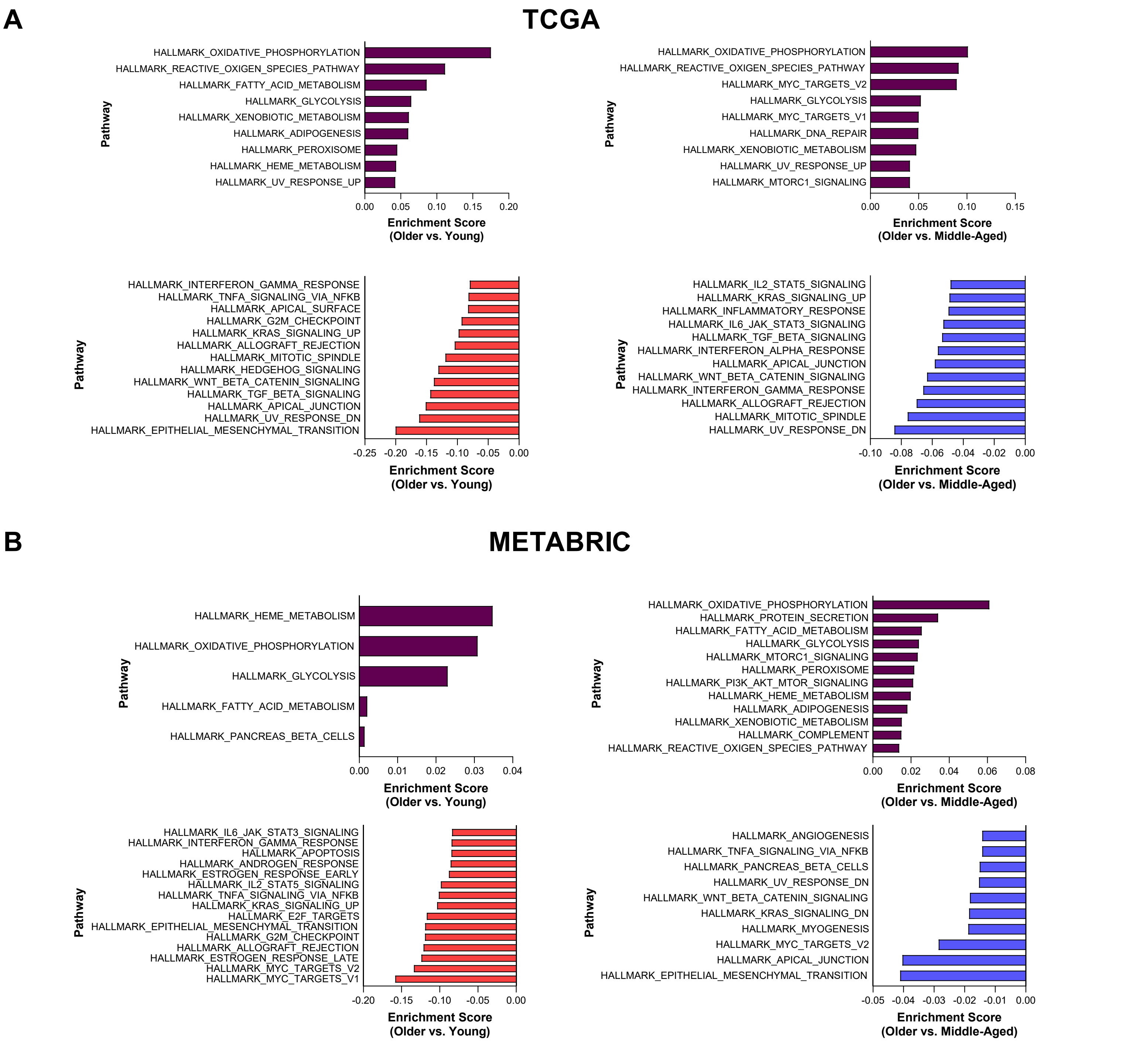

Supplementary Fig. 5: Pathway enrichment comparisons using TCGA and METABRIC data. (A) Shows top and bottom enriched pathways in older patients compared to both middle-aged and younger patients in TCGA. (A) Shows top and bottom enriched pathways in older patients compared to both middle-aged and younger patients in METABRIC.

A

Age_Group	Case ID	Age	Grade	Histological Subtype	Stage	ER	PR	HER2 IHC	HER2 ISH (ratio)	Ki67	Treatment status	Notable Pathological features
O	4290	88	2	IDC	pT4b, Nx	90% 3+	30% 2+	1+	Non-Amplified	10%	Naïve	Locally advanced, skin and chest wall muscle involvement.
O	4067	85	2	IDC	pT2, N1(sn), Mx	100% 3+	95% 3+	1+	Non-Amplified	3-4%	Naïve	Associated low grade DCIS and focal perineural invasion.
O	3948	82	3	IDC	pT2, N2a	90% 2-3+	80% 2+	0	Non-Amplified	10%	Naïve	Associated LCIS, with LVI and perineural invasion
MA	4463	58	2	IDC	pT3, N1, Mx	100% 2-3+	80% 2-3+	0	Non-Amplified	50%	Naïve	IDC with areas of lobular -like growth pattern, but is E-cadherin positive. Associated low through high grade DCIS and LVI.
MA	4040	57	3	IDC	pT2, N0	95% 3+	95% 2-3+	0	Non-Amplified	> 50%	Naïve	Associated high grade DCIS.
MA	4471	55	2	ILC	pT3, pN0 (i+)	100% 3+	100% 3+	0	Non-Amplified	20%	Naïve	None.
MA	4461	54	2	IDC	pT3, N1a, Mx	95% 3+	~5% 3+	2+	Non-Amplified	15%	Naïve	Associated intermediate to high grade DCIS, LVI and perineural invasion.
Y	3941	50	2	IDC	pT1c, N1a, Mx	90% 3+	90% 3+	2+	Non-Amplified	10%	Naïve	Multifocal tumour with associated high grade DCIS
Y	4535	47	2	ILC	pT2, pN0 (i+),Stage IIB	95% 3+	70% 2+	2+	Non-Amplified	10%	Naïve	None.
Y	4530	42	2	IDC	pT3, pN3, pMx, Stage IIIA	95% 2+	95% 3+	1+	Non-Amplified	5%	Naïve	Multifocal tumour with associated high grade DCIS and LVI.

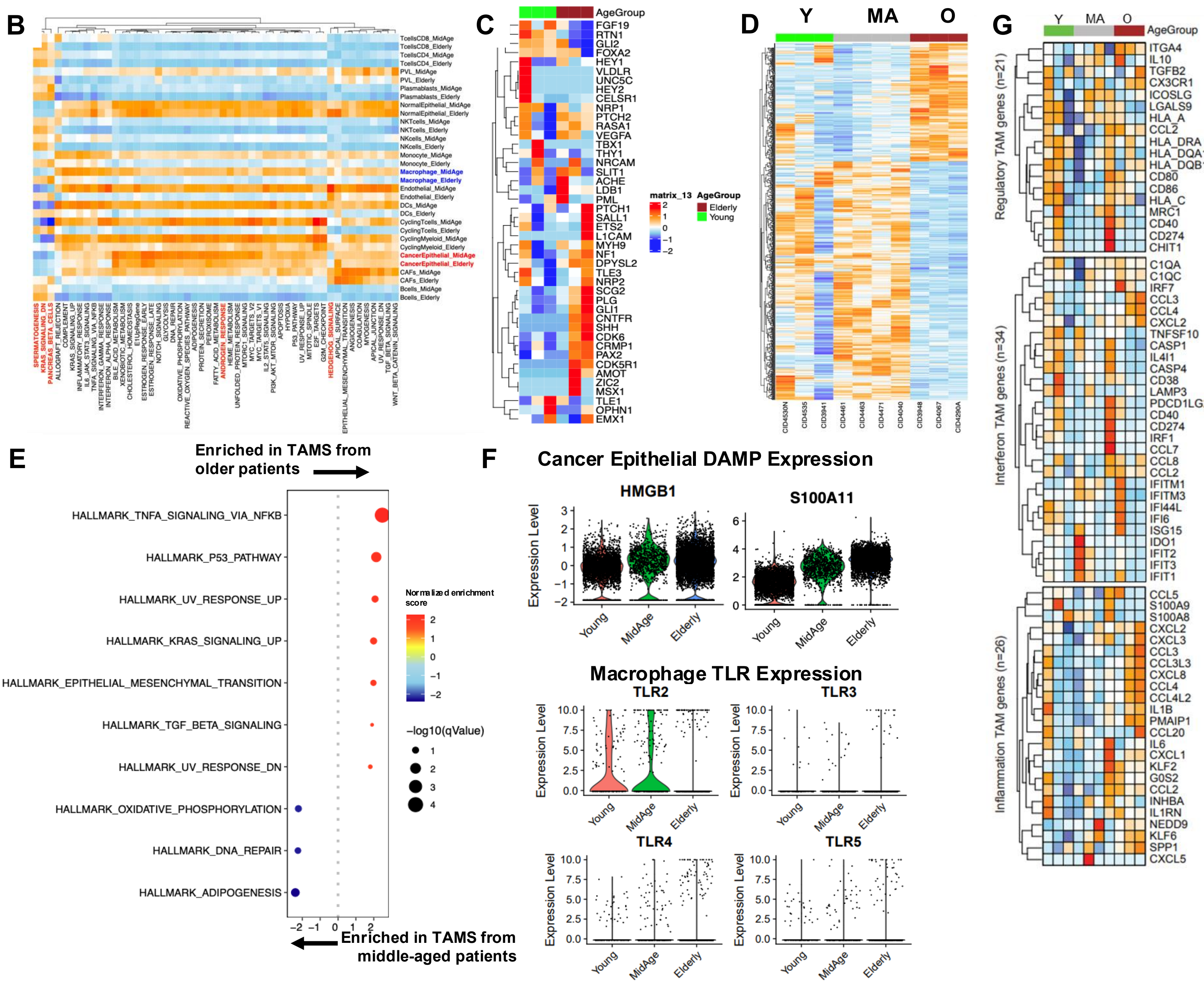

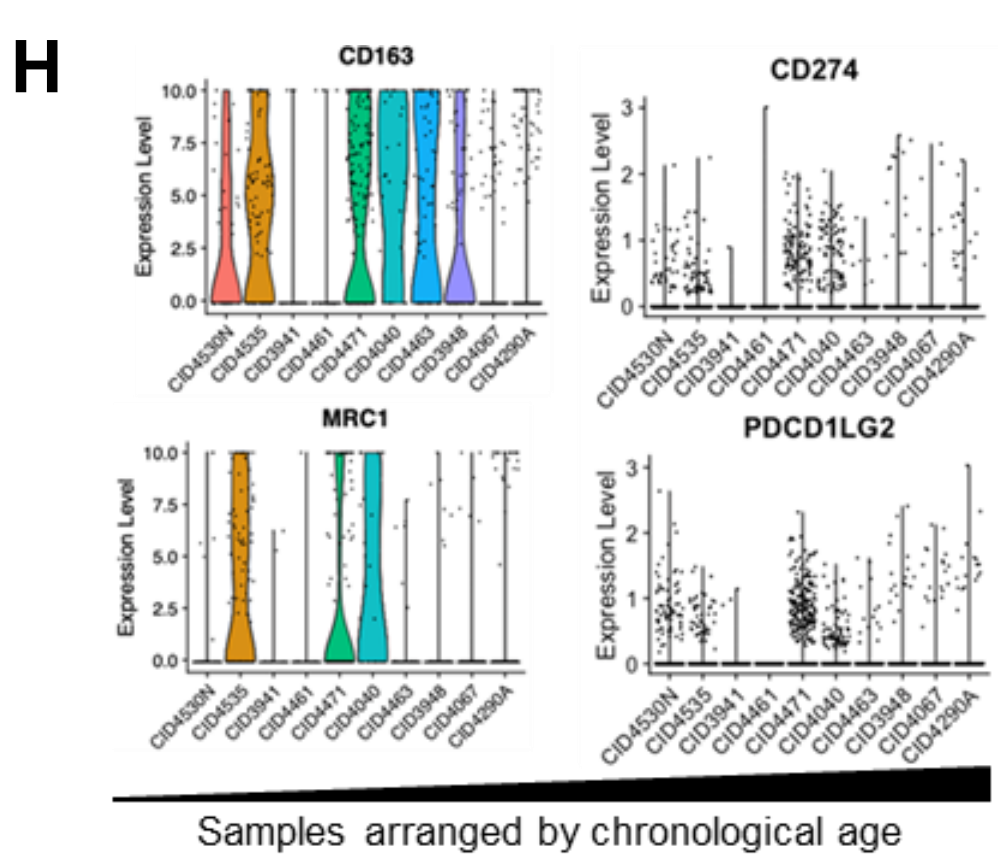

Supplementary Fig. 6: Analysis of macrophages in older patients using publicly-available scRNA-seq data. (A) We analyzed data derived from patients in Wu et al, only including those with treatment-naïve, ER+/HER2- tumors. Table shows the patients that were included. (B) Heatmap showing transcriptomic differences in immune cells from older and middle-aged patients. (C) Heatmap showing expression of Hedgehog pathway genes in macrophages from the tumors of older and younger patients. (D) Heatmap showing DEGs between macrophages in younger, middle-aged, and older tumors. (E) Pathway analysis of the DEGs in macrophages from older and middle-aged patients. (F) Expression of DAMP molecules from cancer epithelial cells across age groups and macrophage toll-like receptor (TLR) expression across age groups. (G) Heatmap showing subtyping of macrophages across age groups. (H) Expression of macrophage markers across tumors according to chronological age.

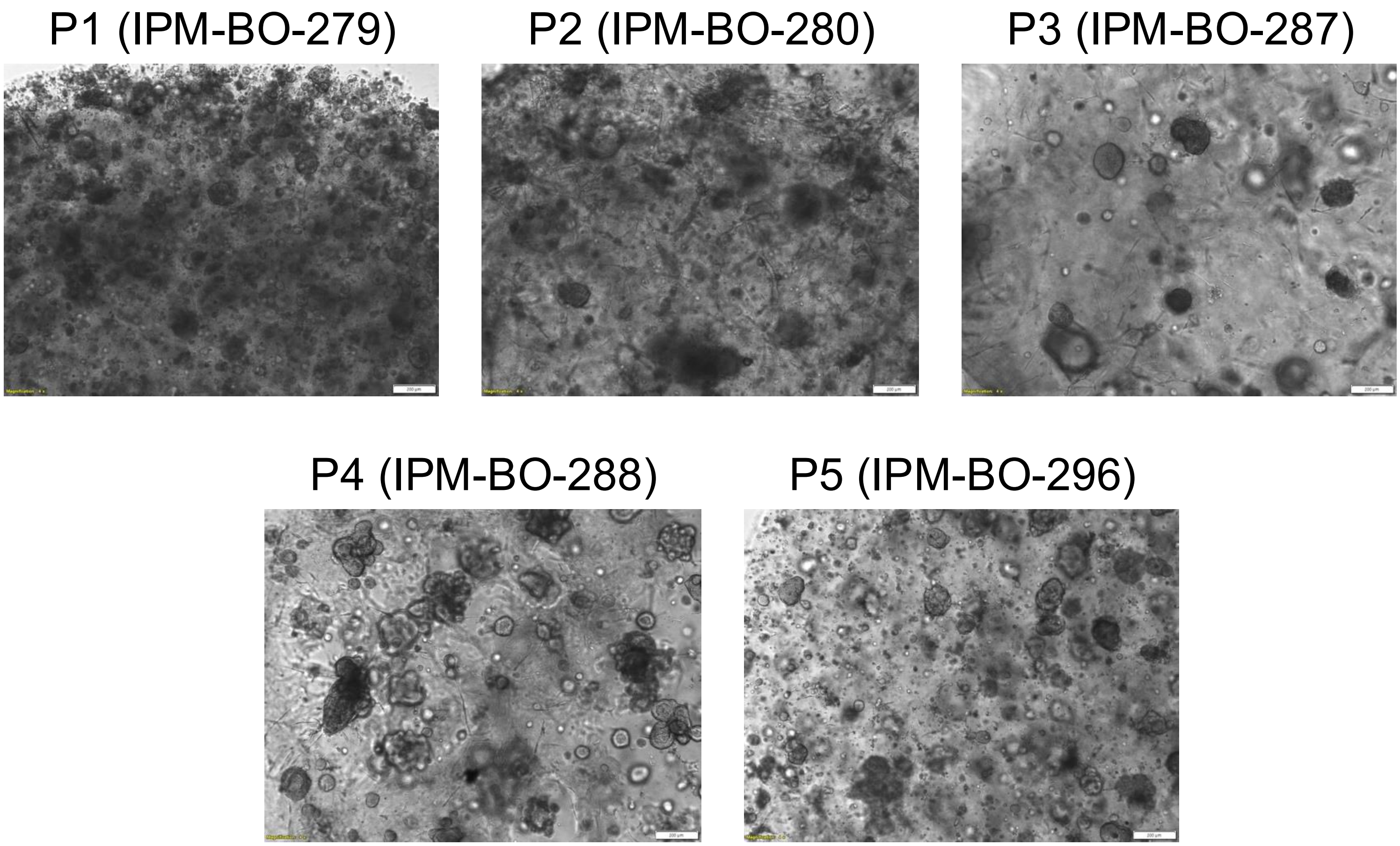

Supplementary Fig. 7: Brightfield images of just-in-culture patient-derived organoids (PDOs). PDOs freshly in culture a diverse milieu of cells (including tumor cells, endothelial cells, lymphocytes, and fibroblasts. Images take at 4x magnification; scale bar represents 200µm.

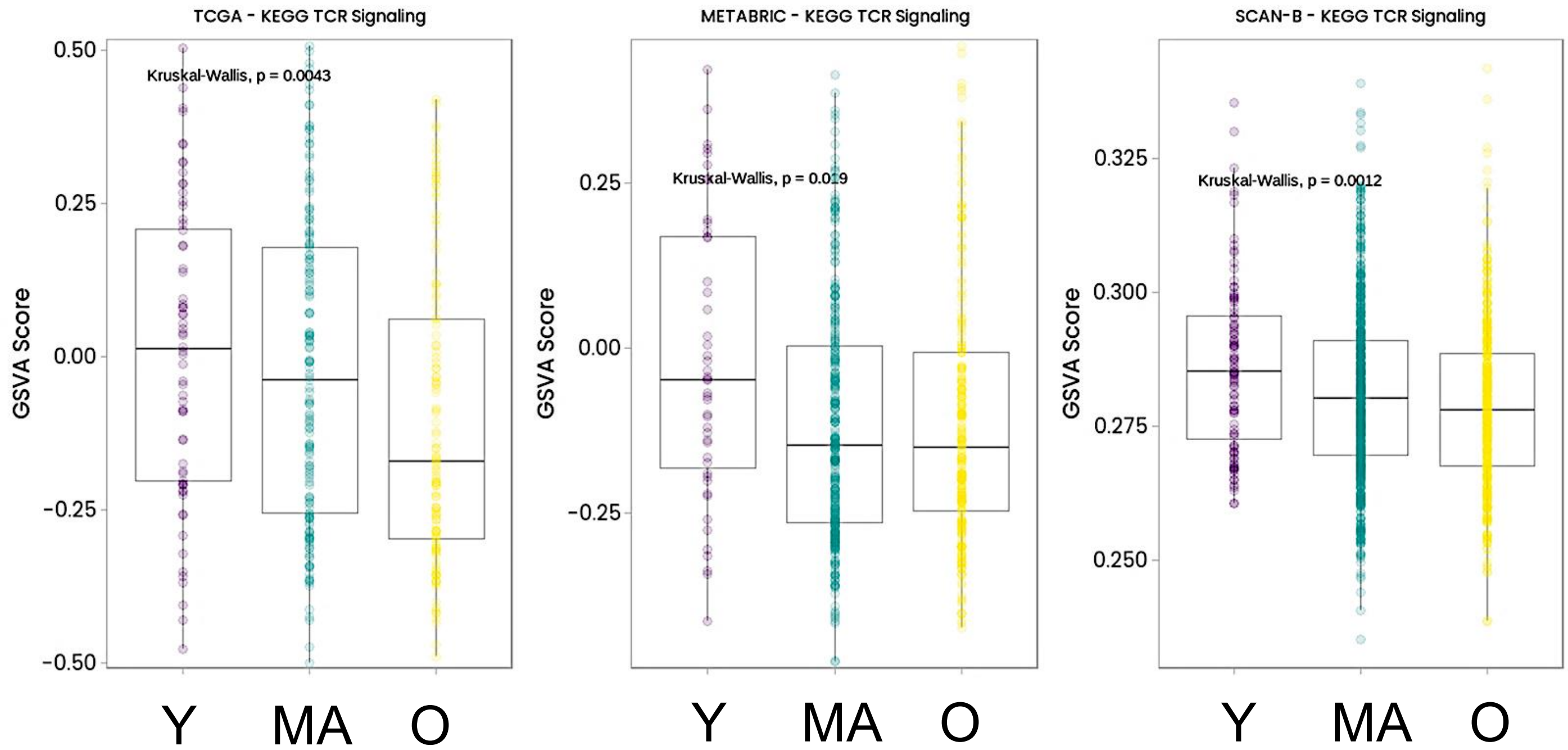

Supplementary Fig. 8: KEGG TCR signaling pathway enrichment across age. Plots show the pathway enrichment levels of the KEGG TCR signaling pathway across the three age groups, with the lowest pathway enrichment level in older patients.

		Young Patients N = 25	Middle-Aged Patients N = 57	Older Patients N = 33	P-value
**Median age at diagnosis, years (IQR)**		39 (37-42)	62 (58.5-65.5)	76 (72.5-79)	< 0.0001
**Median tumor size, mm (IQR)**		21 (17-25)	21 (15-37.5)	21 (12-29)	0.36
**Median BMI, kg/m^2 (IQR)**		23.9 (22.7-26.7)	29.4 (25.1-34.3)	27.5 (23.3-33.9)	0.0001
**Grade at Diagnosis, n (%)**					0.38
	I	3 (12.0%)	3 (5.3%)	2 (6.1%)	
	II	11 (44.0%)	36 (63.1%)	23 (69.7%)	
	III	10 (40.0%)	15 (26.3%)	8 (24.2%)	
	Unknown	1 (4.0%)	3 (5.3%)	0 (0%)	
**Stage at Diagnosis, n (%)**					0.66
	I	12 (48.0%)	24 (42.1%)	10 (30.2%)	
	II	6 (24.0%)	17 (29.8%)	13 (39.4%)	
	III	5 (20.0%)	11 (19.3%)	6 (18.2%)	
	Unknown	2 (8.0%)	5 (8.8%)	4 (12.1%)	
**Histological Subtype, n (%)**					0.67
	IDC / NST	16 (64.0%)	33 (57.9%)	23 (69.7%)	
	ILC	5 (20.0%)	10 (17.5%)	6 (18.2%)	
	Other / Multiple Types Present	4 (16.0%)	14 (24.6%)	4 (12.1%)	
**Menopausal Status, n (%)**					< 0.0001
	Pre-Menopausal	23 (92.0%)	4 (7.0%)	0 (0%)	
	Post-Menopausal	2 (8.0%)	53 (93.0%)	33 (100%)	
**Any History of Smoking, n (%)**					0.90
	Any prior / current use	10 (40.0%)	27 (47.3%)	15 (45.5%)	
	Never Used	14 (56.0%)	30 (52.7%)	18 (54.5%)	
	Unknown	1 (4.0%)	0 (0%)	0 (0%)	
**Any History of EtOH Use, n (%)**					0.51
	Any prior / current use	3 (12.0%)	8 (14.0%)	2 (6.1%)	
	No significant history	21 (84.0%)	49 (86.0%)	31 (93.9%)	
	Unknown	1 (4.0%)	0 (0%)	0 (0%)	
**Patient Race**					0.47
	White	23 (92.0%)	52 (91.2%)	31 (94.0%)	
	Black	2 (8.0%)	5 (8.8%)	1 (3.0%)	
	Other	0 (0%)	0 (0%)	1 (3.0%)	
**Patient Ethnicity**					0.08
	Non-Hispanic	22 (88.0%)	55 (96.5%)	33 (100%)	
	Hispanic	3 (12.0%)	2 (3.5%)	0 (0%)	

Supplementary Table 1: Patient and tumor characteristics from the 115 patients with ER+ breast cancer whose specimens (blood, tumor tissue, and tumor-adjacent tissue) were used in this study.

		Young Donors N = 30	Middle-Aged Donors N = 30	Older Donors N = 29	P-value
**Median age at time of tissue donation, years (IQR)**		37 (36-38.5)	58 (58-59)	74 (71.5-77)	< 0.0001
**Median BMI, kg/m² (IQR)**		24.7 (21.6-26.53)	24.4 (22.5-26.8)	25.8 (23.8-28.3)	0.09
**Age at Menarche, years (IQR)**		13 (11.8-14)	13 (12.8-14)	13 (12-14)	0.25
**Breast Cancer Risk Assessment Gail Score, median (IQR)**		11.3 (9.7-15.7)	8.9 (7.5-13.6)	4.9 (3.9-8.4) *	< 0.0001
**Number of Prior Pregnancies**					0.11
	0	8 (26.7.0%)	5 (16.7%)	1 (3.5%)	
	1	5 (16.7%)	3 (10.0%)	3 (10.3%)	
	≥ 2	17 (56.6%)	22 (73.3%)	25 (86.2%)	
**Any Prior HRT Use #**					< 0.0001
	Yes	0 (0%)	8 (26.7%)	15 (51.7%)	
	No	29 (96.7%)	22 (73.3%)	14 (48.3%)	
	Unknown	1 (3.3%)	0 (0%)	0 (0%)	
**Patient Race**					0.18
	White	28 (93.3%)	30 (100%)	29 (100%)	
	Black	2 (6.7%)	0 (0%)	0 (0%)	
**Patient Ethnicity**					0.35
	Non-Hispanic	26 (86.7%)	26 (86.7%)	28 (96.6%)	
	Hispanic	4 (13.3%)	4 (13.3%)	1 (3.4%)	

* Indicates five missing values for Gail Score within the older donor group.
# No donors with prior HRT use were taking HRT at the time of tissue donation. Information is not available as to the time off of HRT prior to tissue/blood donation.

Supplementary Table 2: Characteristics from the 89 donors (from the Komen Tissue Bank at Indiana University) whose specimens (blood and non-tumor, normal breast tissue) were used in this study. * Indicates five missing values for Gail Score within the older donor group.

Estrone (E1)			Estradiol (E2)	
Dunn's multiple comparisons test	Adjusted P Value		Dunn's multiple comparisons test	Adjusted P Value
Y_BC vs. MA_BC	>0.9999		Y_BC vs. MA_BC	>0.9999
Y_BC vs. O_BC	>0.9999		Y_BC vs. O_BC	>0.9999
Y_BC vs. Y_TA	>0.9999		Y_BC vs. Y_TA	>0.9999
Y_BC vs. MA_TA	0.0171		Y_BC vs. MA_TA	>0.9999
Y_BC vs. O_TA	0.1847		Y_BC vs. O_TA	>0.9999
Y_BC vs. Y_NTD	0.0046		Y_BC vs. Y_NTD	>0.9999
Y_BC vs. MA_NTD	<0.0001		Y_BC vs. MA_NTD	0.0022
Y_BC vs. O_NTD	<0.0001		Y_BC vs. O_NTD	<0.0001
MA_BC vs. O_BC	>0.9999		MA_BC vs. O_BC	>0.9999
MA_BC vs. Y_TA	>0.9999		MA_BC vs. Y_TA	>0.9999
MA_BC vs. MA_TA	>0.9999		MA_BC vs. MA_TA	>0.9999
MA_BC vs. O_TA	>0.9999		MA_BC vs. O_TA	>0.9999
MA_BC vs. Y_NTD	0.9231		MA_BC vs. Y_NTD	0.154
MA_BC vs. MA_NTD	<0.0001		MA_BC vs. MA_NTD	<0.0001
MA_BC vs. O_NTD	0.0003		MA_BC vs. O_NTD	<0.0001
O_BC vs. Y_TA	>0.9999		O_BC vs. Y_TA	>0.9999
O_BC vs. MA_TA	0.0897		O_BC vs. MA_TA	>0.9999
O_BC vs. O_TA	0.7334		O_BC vs. O_TA	>0.9999
O_BC vs. Y_NTD	0.0235		O_BC vs. Y_NTD	>0.9999
O_BC vs. MA_NTD	<0.0001		O_BC vs. MA_NTD	0.0016
O_BC vs. O_NTD	<0.0001		O_BC vs. O_NTD	<0.0001
Y_TA vs. MA_TA	0.0632		Y_TA vs. MA_TA	>0.9999
Y_TA vs. O_TA	0.4687		Y_TA vs. O_TA	>0.9999
Y_TA vs. Y_NTD	0.0167		Y_TA vs. Y_NTD	>0.9999
Y_TA vs. MA_NTD	<0.0001		Y_TA vs. MA_NTD	0.2659
Y_TA vs. O_NTD	<0.0001		Y_TA vs. O_NTD	<0.0001
MA_TA vs. O_TA	>0.9999		MA_TA vs. O_TA	>0.9999
MA_TA vs. Y_NTD	>0.9999		MA_TA vs. Y_NTD	>0.9999
MA_TA vs. MA_NTD	0.0027		MA_TA vs. MA_NTD	0.0004
MA_TA vs. O_NTD	0.0817		MA_TA vs. O_NTD	<0.0001
O_TA vs. Y_NTD	>0.9999		O_TA vs. Y_NTD	>0.9999
O_TA vs. MA_NTD	0.0046		O_TA vs. MA_NTD	0.0518
O_TA vs. O_NTD	0.0868		O_TA vs. O_NTD	<0.0001
Y_NTD vs. MA_NTD	0.2149		Y_NTD vs. MA_NTD	>0.9999
Y_NTD vs. O_NTD	>0.9999		Y_NTD vs. O_NTD	<0.0001
MA_NTD vs. O_NTD	>0.9999		MA_NTD vs. O_NTD	0.0844

Supplementary Table 3: P-values for all statistical comparisons conducted in Figure 4A after correction for multiple comparisons.

Label	Patient Age Range	Tumor Biomarkers	Histology	UPMC Institute of Precision Medicine PDO ID
Patient 1 (P1)	85-89	ER 100, PR 12, HER2 0; Ki-67 90%	IDC	IPM-BO-279
Patient 2 (P2)	80-84	ER 300, PR 1, HER2 0; Ki-67 30%	IDC	IPM-BO-280
Patient 3 (P3)	75-79	ER 290, PR 150, HER2 1+; Ki-67 55%	IDC	IPM-BO-287
Patient 4 (P4)	75-79	ER 300, PR 300, HER2 0; Ki-67 10%	mDLC	IPM-BO-288
Patient 5 (P5)	80-84	ER 250, PR 300, HER2 2+ (not amplified); Ki-67 65%	IDC	IPM-BO-296
Patient 6 (P6)	40-45 (PreM)	ER 290, PR 290, HER2 0; Ki-67 10%	IDC	IPM-BO-056
Patient 7 (P7)	50-54 (PreM)	ER 290, PR 290, HER2 0; Ki-67 5%	IDC	IPM-BO-126
Patient 8 (P8)	75-79	ER 0, PR 0, HER2 0; Ki-67 65%	IDC	IPM-BO-229

Supplementary Table 4: Age and tumor characteristics for the patient derived organoids (PDOs) that were used in this study. All PDOs were collected from specimens at the time of surgery; all patients did not receive neoadjuvant systemic therapy prior to PDO generation. We note that while P8 would have been eligible for neoadjuvant therapy with the Keynote-522 regimen, this patient had a number of comorbidities and opted for upfront surgery.

SUPPLEMENTARY METHODS

Additional details on rat sequencing studies

Rat single-nuclei RNA-sequencing

We performed 3'v3.1 Nuclei Isolation and GEX library preparation with the 10X Genomics Platform. Snap frozen tissues are dissociated with the 10X Genomics Nuclei Isolation Kit. After dissociation, nuclei suspension concentration and nuclei integrity are assessed using AOPI on the Nexcelom Cellometer Auto 2000. Libraries are then prepared according to the Chromium Next GEM Single Cell 3' Reagent Kits v3.1 (Dual Index) protocol using the 10X Genomics Chromium iX Instrument. The resulting full-length cDNA is checked for size and concentration on the Agilent Tapestation 4150 before proceeding with gene expression library preparation and sample indexing. Final libraries are checked for size on the Agilent Tapestation and concentration using the Invitrogen Qubit 2.0. Libraries are pooled and sequenced on the Illumina NovaSeq 6000 Instrument.

Rat whole exome sequencing

Genomic DNA samples were quantified using Qubit 2.0 Fluorometer (ThermoFisher Scientific, Waltham, MA, USA). Enrichment probes were designed against the region of interest and synthesized through Agilent Technologies–SureSelectXT CD Rat All Exon. Library preparation was performed according to the manufacturer's guidelines. Briefly, the genomic DNA was fragmented by acoustic shearing with a Covaris S220 instrument. Fragmented DNAs were cleaned up and end repaired, as well as adenylated at the 3'ends. Adapters were ligated to the DNA fragments, and adapter-ligated DNA fragments were enriched with limited cycle PCR. Adapter-ligated DNA fragments were validated using Agilent TapeStation (Agilent Technologies, Palo Alto, CA, USA), and quantified using Qubit 2.0 Fluorometer. Adapter-ligated DNA fragments were hybridized with biotinylated baits. The hybrid DNAs were captured by streptavidin-coated binding beads. After extensive wash, the captured DNAs were amplified and indexed with Illumina indexing primers. Post-captured DNA libraries were validated using Agilent TapeStation (Agilent, Santa Clara, CA, USA) and quantified using Qubit 2.0 Fluorometer and Real-Time PCR (KAPA Biosystems, Wilmington, MA, USA). The sequencing libraries were multiplexed and clustered onto a flowcell on the Illumina NovaSeq instrument according to manufacturer's instructions. The samples were sequenced using a 2x150bp Paired End (PE) configuration. Image analysis and base calling were conducted by the NovaSeq Control Software (NCS). Raw sequence data (.bcl files) generated from Illumina NovaSeq was converted into fastq files and demultiplexed using Illumina bcl2fastq 2.20 software. One mis-match was allowed for index sequence identification.

Sequencing results were aligned and analyzed using version 3.4.0 of the NF-Core Sarek Pipeline, using Nextflow version 23.10.1, Singularity 3.9.6. Pipeline settings used the Singularity profile, Rnor_6.0 Rat genome, CNVKit and Mutect2 tools, skipped base_recalibrator, WES-only mode with a minimal SLURM config file for the University of Pittsburgh Center for Research

Computing High Throughput Computing Cluster. Mutect2.vcf.gz outputs were then annotated using VEP 95. High impact and Moderate Impact mutations as defined by Ensembl were then extracted for analysis. Rat genes were made comparable to human homologs using PyBioMart, and then filtered down to a list of pan-cancer and breast cancer genes (See supplementary information), in cases where multiple gene mutations were found in the same gene, ties were broken by prioritizing greatest impact followed by frequency. Results were then plotted in an Oncoplot. Tumor Mutational Burden was calculated using the whole exome length of the rat genome of 71Mb. K-means clustering was done by selecting all genes affected by moderate and high impact mutations and binarized into a corresponding matrix. Predicted labels were then evaluated to see if the two populations of tumor were distinctly separable based on mutational characteristics. Mutational Signature Analysis was done using SigProfilerAssignment, with context 96 and genome Rnor_6.0. CNVKit analysis was run in tumor-only mode, without normal control due to a sample quality issue that affected CNVKit output.

Rat bulk RNA-sequencing

Total RNA was extracted from fresh frozen tissue samples using Qiagen RNeasy Plus Universal mini kit following manufacturer’s instructions (Qiagen, Hilden, Germany). RNA samples were quantified using Qubit 2.0 Fluorometer (ThermoFisher Scientific, Waltham, MA, USA) and RNA integrity was checked with 4200 TapeStation (Agilent Technologies, Palo Alto, CA, USA). ERCC Ex-fold RNA reagent (Cat: 4456739) from ThermoFisher Scientific, was added to normalized total RNA prior to library preparation following manufacturer’s protocol. The next steps included performing rRNA depletion using QIAseq® FastSelect™−rRNA HMR kit (Qiagen, Germantown, MD, USA), which was conducted following the manufacturer’s protocol. RNA sequencing libraries were constructed with the NEBNext Ultra II RNA Library Preparation Kit for Illumina by following the manufacturer’s recommendations. Briefly, enriched RNAs are fragmented for 15 minutes at 94°C. First strand and second strand cDNA are subsequently synthesized. cDNA fragments are end repaired and adenylated at 3’ends, and universal adapters are ligated to cDNA fragments, followed by index addition and library enrichment with limited cycle PCR. Sequencing libraries were validated using the Agilent Tapestation 4200 (Agilent Technologies, Palo Alto, CA, USA), and quantified using Qubit 2.0 Fluorometer (ThermoFisher Scientific, Waltham, MA, USA) as well as by quantitative PCR (KAPA Biosystems, Wilmington, MA, USA). The sequencing libraries were multiplexed and clustered onto a flowcell on the Illumina NovaSeq instrument according to manufacturer’s instructions. The samples were sequenced using a 2x150bp Paired End (PE) configuration. Image analysis and base calling were conducted by the NovaSeq Control Software (NCS). Raw sequence data (.bcl files) generated from Illumina NovaSeq was converted into fastq files and de-multiplexed using Illumina bcl2fastq 2.20 software. One mis-match was allowed for index sequence identification. We used FastQC/0.11.7 to check the quality of reads followed by Trimmomatic/0.38 to remove the adapter contents. High quality reads(Q>30) were used for alignment using Star/2.7.9a followed by Picard/2.18.12 to mark duplicate and then run HTSeq/0.13.5 to count the reads and then DESeq2 to identify differentially expressed genes.
