# Supplementary material for: Systemic and local chronic inflammation and hormone disposition promote a tumor-permissive environment for breast cancer in older women
Source: Nat Aging. 2026 Jul 27;6(8):1695–716. doi: 10.1038/s43587-026-01173-4 (PMC13472878; doi:10.1038/s43587-026-01173-4)
Supplement: Supplementary file 2 [file 43587_2026_1173_MOESM2_ESM.pdf]

# nature portfolio

Corresponding author(s): Adrian Lee, PhD

Last updated by author(s): Jun 10, 2026

# Reporting Summary

Nature Portfolio wishes to improve the reproducibility of the work that we publish. This form provides structure for consistency and transparency in reporting. For further information on Nature Portfolio policies, see our Editorial Policies and the Editorial Policy Checklist.

## Statistics

For all statistical analyses, confirm that the following items are present in the figure legend, table legend, main text, or Methods section.

| n/a | Confirmed | |
|---|---|---|
| ☐ | ☒ | The exact sample size ($n$) for each experimental group/condition, given as a discrete number and unit of measurement |
| ☐ | ☒ | A statement on whether measurements were taken from distinct samples or whether the same sample was measured repeatedly |
| ☐ | ☒ | The statistical test(s) used AND whether they are one- or two-sided<br>*Only common tests should be described solely by name; describe more complex techniques in the Methods section.* |
| ☒ | ☐ | A description of all covariates tested |
| ☐ | ☒ | A description of any assumptions or corrections, such as tests of normality and adjustment for multiple comparisons |
| ☐ | ☒ | A full description of the statistical parameters including central tendency (e.g. means) or other basic estimates (e.g. regression coefficient) AND variation (e.g. standard deviation) or associated estimates of uncertainty (e.g. confidence intervals) |
| ☐ | ☒ | For null hypothesis testing, the test statistic (e.g. $F$, $t$, $r$) with confidence intervals, effect sizes, degrees of freedom and $P$ value noted<br>*Give P values as exact values whenever suitable.* |
| ☒ | ☐ | For Bayesian analysis, information on the choice of priors and Markov chain Monte Carlo settings |
| ☒ | ☐ | For hierarchical and complex designs, identification of the appropriate level for tests and full reporting of outcomes |
| ☐ | ☒ | Estimates of effect sizes (e.g. Cohen's $d$, Pearson's $r$), indicating how they were calculated |

*Our web collection on statistics for biologists contains articles on many of the points above.*

## Software and code

Policy information about availability of computer code

| | |
|---|---|
| Data collection | No software was used to collect data in this study. |
| Data analysis | Data analysis included use of R and Python. All code is available through Github at: https://github.com/leeoesterreich/ERpBRCA_OlderWomen?tab=readme-ov-file. |

For manuscripts utilizing custom algorithms or software that are central to the research but not yet described in published literature, software must be made available to editors and reviewers. We strongly encourage code deposition in a community repository (e.g. GitHub). See the Nature Portfolio guidelines for submitting code & software for further information.

## Data

Policy information about availability of data

All manuscripts must include a data availability statement. This statement should provide the following information, where applicable:

- Accession codes, unique identifiers, or web links for publicly available datasets
- A description of any restrictions on data availability
- For clinical datasets or third party data, please ensure that the statement adheres to our policy

Source data is provided as supplementary material. All raw genomic data was deposited to Gene Expression Omnibus (GEO) under the following accession numbers:
• GSE276755 (human tumor and tumor-adjacent bulk RNA-seq)
• GSE276757 (rat tumor bulk RNA-seq)

- GSE276759 (rat tumor whole exome sequencing)
- GSE276758 (rat tumor single-nuclei RNA-seq)
- GSE325982 (single cell RNA-sequencing of patient derived organoid)

Proteomic data derived from the Luminex platform quantifying inflammatory panels is deposited to Figshare and is available for download at the following link: https://doi.org/10.6084/m9.figshare.32620215.

## Research involving human participants, their data, or biological material

Policy information about studies with human participants or human data. See also policy information about sex, gender (identity/presentation), and sexual orientation and race, ethnicity and racism.

| | |
|---|---|
| Reporting on sex and gender | This research only included female rats, women donors of non-cancerous breast tissue, and women patients with ER+/HER2- breast cancer given that the vast majority of breast cancer occurs in women. Gender information was collected from human subjects and thus the term women is used throughout the manuscript. |
| Reporting on race, ethnicity, or other socially relevant groupings | Details on race and ethnicity are included in the supplementary tables as reported by the donors or patients. We did not perform subgroup analysis according to race or ethnicity. |
| Population characteristics | Please see supplementary tables for population and clinical characteristics for the donors and patients included in this study. Age, gender, race and ethnicity, smoking history, details on breast cancer diagnoses, and hormone replacement therapy use are included. |
| Recruitment | Donors and patients were selected based on their age in a nonrandomized fashion. |
| Ethics oversight | University of Pittsburgh IRB; Indiana University IRB. All participants / donors of biospecimens provided written informed consent for their biospecimens to be included in the study. No participants were compensated. This study used biospecimens collection under a clinical trial (NCT05914792). |

Note that full information on the approval of the study protocol must also be provided in the manuscript.

# Field-specific reporting

Please select the one below that is the best fit for your research. If you are not sure, read the appropriate sections before making your selection.

☒ Life sciences ☐ Behavioural & social sciences ☐ Ecological, evolutionary & environmental sciences

For a reference copy of the document with all sections, see nature.com/documents/nr-reporting-summary-flat.pdf

# Life sciences study design

All studies must disclose on these points even when the disclosure is negative.

| | |
|---|---|
| Sample size | No samples size calculation was performed. Sample sizes were determined based on biospecimen availability. |
| Data exclusions | No data were excluded. |
| Replication | All experiments were replicated at least three times without discordant results. |
| Randomization | There was no randomization in this study. |
| Blinding | Experiments using archived frozen and FFPE human specimens were coded, processed, and analyzed in a blinded fashion. Studies using prospectively collected patient derived organoids were not blinded; however, data was analyzed using imaging methodologies that limited bias. |

# Reporting for specific materials, systems and methods

We require information from authors about some types of materials, experimental systems and methods used in many studies. Here, indicate whether each material, system or method listed is relevant to your study. If you are not sure if a list item applies to your research, read the appropriate section before selecting a response.

## Materials & experimental systems

| n/a | Involved in the study |
|---|---|
| ☐ | ☒ Antibodies |
| ☐ | ☒ Eukaryotic cell lines |
| ☒ | ☐ Palaeontology and archaeology |
| ☐ | ☒ Animals and other organisms |
| ☐ | ☒ Clinical data |
| ☒ | ☐ Dual use research of concern |
| ☒ | ☐ Plants |

## Methods

| n/a | Involved in the study |
|---|---|
| ☒ | ☐ ChIP-seq |
| ☒ | ☐ Flow cytometry |
| ☒ | ☐ MRI-based neuroimaging |

# Antibodies

| | |
|---|---|
| Antibodies used | Rat Tumor IHC: ER (abcam, #ab32063, 1:10,000 dilution) and Ki-67 (Novus Biologicals, NB600-1252, 1:100 dilution); IF for macrophage marker staining: CD206 (Biolegend, #321102) and PD-L1 (Biolegend, #329701). |
| Validation | All antibodies used were validated by the manufacturers. |

# Eukaryotic cell lines

Policy information about cell lines and Sex and Gender in Research

| | |
|---|---|
| Cell line source(s) | MCF7, T47D, and MM231. |
| Authentication | All cell lines were authenticated through IDEXX BioAnalytics. |
| Mycoplasma contamination | We confirm all cell lines used were tested and free of contamination from mycoplasma. |
| Commonly misidentified lines (See ICLAC register) | NA. |

# Animals and other research organisms

Policy information about studies involving animals; ARRIVE guidelines recommended for reporting animal research, and Sex and Gender in Research

| | |
|---|---|
| Laboratory animals | All animals were housed in The Ford / Assembly Research Building at the University of Pittsburgh in an approved animal facility. All animal experiments were performed following protocols 20108061 and 23103979, which were both approved by the University of Pittsburgh Institutional Animal Care and Use Committee. Virgin Fischer 344 (F344) rats aged 6-8 weeks old were purchased from Envigo and served as the "young" rats in the study. Aged F344 rats were obtained from the National Institutes of Aging (NIA) Rodent Colonies at 19-21 months old and served as the "older" rats in the study. All rats were housed in, ventilated autoclaved caging systems (Allentown Caging) on hard chip bedding, had 12hr/12hr light-dark cycles, were provided with hyperchlorinated RO water, and were fed ad libitum rodent chow (Purina). Rats lived in the facility for at least two months prior to any experimental treatment to allow for equilibration. |
| Wild animals | Study did not involve wild animals. |
| Reporting on sex | Findings only apply to one sex (females). |
| Field-collected samples | Study did not involve samples collected from the field. |
| Ethics oversight | All animal experiments were performed following protocols 20108061 and 23103979, which were both approved by the University of Pittsburgh Institutional Animal Care and Use Committee. |

Note that full information on the approval of the study protocol must also be provided in the manuscript.

# Clinical data

Policy information about clinical studies

All manuscripts should comply with the ICMJE guidelines for publication of clinical research and a completed CONSORT checklist must be included with all submissions.

| | |
|---|---|
| Clinical trial registration | This study used biospecimens collected under a previously completed clinical trial (NCT05914792). |
| Study protocol | The study protocol / details is published under Carleton N et al, Clinical Cancer Research, 2026. |
| Data collection | Data was collected at the University of Pittsburgh from 2022-2025. |

| | |
|---|---|
| Outcomes | Trial details are published under Carleton N et al, Clinical Cancer Research, 2026. |

## Plants

| | |
|---|---|
| Seed stocks | NA |
| Novel plant genotypes | NA |
| Authentication | NA |
