# Supplementary material for: Systemic and local chronic inflammation and hormone disposition promote a tumor-permissive environment for breast cancer in older women
Source: Nat Aging. 2026 Jul 27;6(8):1695–716. doi: 10.1038/s43587-026-01173-4 (PMC13472878; doi:10.1038/s43587-026-01173-4)
Supplement: Supplementary file 3 [file 43587_2026_1173_MOESM3_ESM.pdf]

nature portfolio

Peer Review File

Systemic and local chronic inflammation and hormone disposition promote a tumor-permissive environment for breast cancer in older women

Corresponding Author: Dr Adrian Lee

This manuscript has been previously reviewed at another journal that is not operating a transparent peer review scheme. The manuscript was considered suitable for publication without further review at Nature Aging.

Version 1:

Reviewer comments:

Reviewer #1

(Remarks to the Author)
The authorss present a revised version of their manuscript examining how aging associated changes in systemic inflammation and hormone metabolism shape the tumor microenvironment in breast cancer. The study integrates an aged rat model, human biospecimens, large transcriptomic datasets, and patient-derived organoids , and proposes that a chemokine-enriched inflammatory state and local estrogen conversion (via HSD17B7) together promote an immunosuppressive, tumor-permissive environment in older patients.

The revised manuscript is substantially improved- In particular, the authors have clarified the conceptual framework around chronic inflammation, better distinguished correlation from mechanism, and added meaningful functional data (notably organoids-based inhibition studies and expanded macrophage analyses). These additions strengthen the overall narrative and enhance the translational relevance of the work.

Despite these improvements, several important issues remain, primarily related to the strength of mechanistic conclusions relative to the available data.

Major Comments

1. The revised manuscript more clearly distinguishes association from mechanism, which is appreciated. However, the central model—linking aging, systemic inflammation, local estrogen conversion, and immune suppression—still relies largely on associative human and animal data, and tissue-level functional experiments (organoids and macrophage assays).

There remains no direct in vivo evidence demonstrating that HSD17B7 is required for tumor development or progression in aged systems, or chemokine signaling (eg, CCL2) causally drives macrophage-mediated immune suppression in vivo. While the authors appropriately acknowledge these limitations, some statements in the manuscript still imply a level of causality and translational readiness that is not fully supported.

Please further temper causal and therapeutic claims throughout, and consistently distinguish between association, ex vivo mechanism, and untested in vivo causality.

2. The authors provide a clearer justification for the use of the F34 rat model, which is helpful. However as acknowledged-- this model does not fully recapitulate human postmenopausal estrogen biology, particularly the shift toward estrone predominance. Given that the HSD17B7 hypothesis depends on E1-to-E2 conversion, this limitation continues to weaken the integration between the animal model and the human findings.
More explicitly separate conclusions derived from the rat model (aging and immune context) from those derived from human and PDO analyses (estrogen metabolism).

2. The addition of organoid-based inhibition experiments is a clear strength of the revision and provides important support for

the proposed role of HSD17B7.

However, validation remains limited to pharmacologic inhibition, no genetic perturbation (e.g knockdown/CRISPR) is included and some effects in E2-treated conditions raise questions about specificity

Thus, HSD17B7 is well-supported as a candidate target but not yet fully validated as a therapeutic mechanism.

Please moderate claims accordingly and clearly frame HSD17B7 as a promising but not yet definitive target.

3. The revised manuscript includes stronger evidence for immunosuppressive macrophage states, including spatial analyses and improved pathway characterization. However, the current framework still simplifies macrophage biology, which is highly heterogeneous. In addition, as far as I can tell, functional consequences for T cells remain largely inferred and the relative importance of specific chemokines is not fully resolved. Expand discussion of macrophage heterogeneity and avoid overgeneralizing polarization states.

Minor Comments

1. Provide clearer explanation of analytical approaches (for instyance, MICA) for broader readership.
2. Ensure all sample sizes are explicitly stated in figures and legends.
3. Clarify inclusion criteria and handling of missing data in human cohorts.
4. Consider adding a schematic distinguishing associative findings, functional (ex vivo) evidence
and untested causal mechanisms..

Acceptable after further revision focused on refining interpretation and appropriately calibrating mechanistic and translational claims.

(Remarks on code availability)

Reviewer #2

(Remarks to the Author)
The authors have satisfactorily addressed the prior critiques.

(Remarks on code availability)

Reviewer #3

(Remarks to the Author)
Thank you for addressing my comments, no further concerns.

(Remarks on code availability)

Open Access This Peer Review File is licensed under a Creative Commons Attribution 4.0 International License, which permits use, sharing, adaptation, distribution and reproduction in any medium or format, as long as you give appropriate credit to the original author(s) and the source, provide a link to the Creative Commons license, and indicate if changes were made.
In cases where reviewers are anonymous, credit should be given to 'Anonymous Referee' and the source.
The images or other third party material in this Peer Review File are included in the article's Creative Commons license, unless indicated otherwise in a credit line to the material. If material is not included in the article's Creative Commons license and your intended use is not permitted by statutory regulation or exceeds the permitted use, you will need to obtain permission directly from the copyright holder.
To view a copy of this license, visit https://creativecommons.org/licenses/by/4.0/

24 March 2026

NATAGING-A09190A – Systemic and local chronic inflammation and hormone disposition promote a tumor-permissive environment for breast cancer in older women

Response to Editor & Reviewers

Dear Dr. Thuault & Dr. Walters,

We enclose the reviewer comments from our *Nature Cancer* (NATCANCER-A16752) submission. We thank the editors of *Nature Cancer* and the reviewers for a thorough evaluation of our submission and appreciate the editors of *Nature Aging* for considering a revised version of the manuscript.

We were encouraged to see many positive comments from all the reviewers, such as the translational implications of the work (noted by all three of the reviewers) and the use of multiple model systems (specifically the aged rat model and the broad range of human specimens and human patient derived organoids). We enclose our point-by-point response to the reviewers below (reviewer comments in black, our response in red) and believe that the reviewer comments have strengthened the manuscript. We note a number of new experiments and data, including testing of the HSD17B7 inhibitor in patient derived organoids and new spatial transcriptomic analysis of the aged human breast TME.

The cancer and aging field (and in particular, the breast cancer and aging field) has seen a growing interest in understanding the intricacies of these intertwined topics, for example, as highlighted in the recent review by Trastus and Fagagna in *Nature Aging*. *Nature Aging* is at the forefront of this work, highlighted by multiple recent papers that investigate the aged mouse mammary gland (Angarola et al, 2024) and the cell interactome in the aged breast (Parsons et al, 2025). We believe our paper contributes to this growing field and substantially extends upon the prior findings with human translational models and results not found in these prior studies. This is a strength of our work, highlighted by a large cohort of patients with ER+ breast cancer, normal breast tissue from individuals without breast cancer from the Komen Tissue Bank, and prospectively collected and generated breast cancer patient-derived organoids from older women. Given these strengths, we envision attracting readership interest not only in the cancer and aging field, but also among clinicians treating patients with breast cancer.

We thank you again for considering our revised manuscript. We look forward to hearing from you soon.

Sincerely,

Adrian Lee, PhD
Steffi Oesterreich, PhD
Neil Carleton, MD, PhD

Reviewer #1:

This study provides a strong and well-researched analysis of how chronic inflammation and hormone metabolism contribute to a tumor-permissive environment in estrogen receptor-positive (ER+) breast cancer in older women. By combining human samples with an aged rat model, the authors effectively highlight the interplay between systemic inflammation, local estrogen conversion, and immune suppression. The data suggest that HSD17B7 plays a central role in estrogen metabolism within the tumor microenvironment and that chemokine-mediated immune suppression, particularly through CD206+ macrophages, may further promote tumor progression.

[R1-1] While these findings are compelling, I suggest a major revision of the study, since it would benefit from a clearer distinction between correlation and causation. Chronic inflammation, as discussed here, is a broadly used and sometimes ill-defined term, and its role in aging and cancer has been explored in prior studies. The manuscript should integrate this perspective more explicitly to position its novel contributions within the broader scientific landscape. A refined definition of chronic inflammation and its distinct molecular pathways would enhance the clarity and impact of the findings.

We appreciate the reviewer's point regarding the need to more clearly distinguish correlation from causation and to more precisely define chronic inflammation in the context of aging. We agree that the term is often used broadly and can blur biologically distinct inflammatory programs.

In this revised manuscript, we have refined our use of the term "chronic inflammation" to reflect an operational definition aligned with aging biology: a low-grade, persistent, chemokine-biased inflammatory state that alters immune cell recruitment and function rather than inducing canonical acute inflammatory signaling. While prior studies have emphasized IL-1β, IL-6, and IL-8 as markers of inflammaging, our data indicate that age-associated inflammation in both humans and aged rats is most strongly characterized by chemokines, particularly CCL2 and related axes, though this is less well-defined in prior reviews and reports on chronic inflammation.

As a response to the reviewer, we now explicitly distinguish association from mechanism throughout the manuscript. Systemic chemokine elevation is described as correlational, whereas mechanistic inference is restricted to the local tumor microenvironment: using in vitro macrophage polarization assays and patient-derived organoid conditioned media, we demonstrate that the combination of chemokines and elevated local E2 is sufficient to drive immunosuppressive macrophage polarization, establishing a causal link at the tissue level. To further strengthen this mechanism, we test a number of relevant inhibitors, including carlumab (CCL2-targeting mAb, anti-estrogen receptor degrader fulvestrant, and a novel HSD17B7 inhibitor).

To address this comment, we have added a new paragraph to the introduction in lines 122-134 on chronic inflammation as well as a new paragraph on limitations in the discussion in lines 663-675.

[R1-2] The methodology is robust, incorporating single-cell RNA sequencing, tumor characterization, and inflammatory marker analysis. However, some aspects require further justification and refinement. The choice of Fischer 344 (F344) rats is reasonable, but it would be useful to elaborate on why this model was selected over others. Additionally, longitudinal immune profiling before and after tumor development would offer deeper insights into how inflammation and hormonal shifts and trajectories evolve over time.

The F344 rats were selected for several reasons: first and most notably, immuno-competent models of ER+ tumors in mice are rare. Mice also have additional limitations, such as lack of a true estropause period in their lifecycle where estradiol decreases. Our preliminary experiments suggested that rat estradiol decreased with age (likely around the 12-13mo mark), and we could also reliably generate ER+ tumors with the DMBA/MPA model of carcinogenesis. An additional reason is that the F344 strain is one that the National Institute on Aging (NIA) and investigators commonly use for aging studies, and as such, NIA provides them to researchers at advanced ages. Under our grant, we could obtain these aged rats at 19-21 months (which are only available commercially aged to 12-13 months). F344 rats have been increasingly used in aging and cancer research,

highly by a recent paper by Yan et al, 2024, Cancer Cell; PMID: 39366375). We have added a new paragraph to the methods as elaboration (lines 726-730).

Additionally, while we agree that before/after analysis would be valuable to assess circulating and local TME changes in the rats, this is unfortunately not possible at this time as we did not collect the specimens. While we did consider doing this, we noticed that while the aged rats were living prior to any experimental manipulation, a few did pass away from unknown / age-related causes. Thus, we did not want to risk comprising our ultimate experimental goal (observing changes in tumor development and growth) by drawing blood or collecting tissue prior to these endpoints.

[R1-3] The study's findings on HSD17B7 and estrogen metabolism are particularly intriguing, but stronger experimental validation is needed. An in vivo knockdown or pharmacological inhibition study would help confirm its role in tumor progression.

We appreciate the reviewer's comment on this. This is a comment shared by Reviewer 2 – we also agree that this is an important finding with potential translational opportunity. However, we recognize that there are a number of limitations at the current time that preclude further investigation without significant optimization.

We have certainly considered an in vivo pharmacological inhibition experiment but felt that the timeline to perform such studies, as the carcinogen treatment takes 1-2 years to develop mammary carcinomas, was not practical for this submission. An additional technical challenge is that aged rats from the NIA are only available in limited supply. We have tried to overcome this technical challenge by developing transplantable tumors but thus far have not been successful. A further consideration is that while the F344 rats do in fact have a physiological decrease in E2 as they age (similar to humans), E1 does NOT become the predominant circulating estrogen. In fact, our data suggest that E1 is quite low throughout the rat's lifespan (as we describe in **Extended Data Figure 1C**). This is a limitation of the F344 rat model, and supplemental E1 would be needed to overcome this limitation. Lastly, in collaboration with our medicinal chemist and co-author, Dr. Donald Poirier, we would need to resynthesize the HSD17B7 at quantities sufficient for daily dosing over many days and in large enough quantities sufficient for the rats' weight. Given the need for new synthesis, this would require additional rats from the NIA and time to complete the experiment. Collectively, while we do agree that this would be a much-needed next step towards translation of this therapeutic approach, we believe this may be outside of the scope of this work but important for future studies.

However, to address the reviewer's comments, we have now completed additional in vitro experiments evaluating the HSD17B7 inhibitor in the PDOs collected from older patients with ER+/HER2- breast cancer. In these PDOs, we show across numerous readouts, such as qRT-PCR (**Fig. 5B and 5C**), FACS for cell cycle analysis (**Fig. 5B**), and scRNA-seq (**Figs. 5D through 5I and Extended Data Fig. 8**), that HSD17B7 inhibition after E1 treatment shows significant anti-estrogen and anti-proliferative effects. In the scRNA-seq experiment, we treated a PDO with seven conditions: vehicle, E1 alone, E1 with the HSD17B7 inhibitor, E1 with fulvestrant, E2 alone, E2 with the HSD17B7 inhibitor, and E2 with fulvestrant. We were excited to see efficacy of the inhibitor in this setting and show that the inhibitor targets the transcriptional and metabolic sub-programs of estrogen response. At the pathway level, broad stress programs are activated with the inhibitor while pathways related to the cell cycle and intracellular metabolism are suppressed.

Given this new data, we have moved all of the studies on HSD17B7 inhibition to a new figure, **Figure 5**. We believe that these results greatly strengthen the manuscript, specifically the findings related to the novel inhibitor.

[R1-4] Similarly, the presence of CD206+ macrophages in the tumor microenvironment raises interesting questions about immune suppression, but the underlying signaling pathways governing their polarization remain unclear. A transcriptomic analysis of these macrophages could shed light on their functional role in shaping the immune landscape.

We agree that this additional data would be beneficial. To this end, building on our unique access to patient samples and the rich cohort we built, we collected a cohort (n=6) of specimens from a recently completed trial (NCT05914792; published in *Clinical Cancer Research*) that our group executed and performed spatial transcriptomics and proteomics from older patients with ER+/HER2- breast cancer. In this analysis, where we specifically focused on cancer epithelial cells, T cells, and macrophages, we were excited to find that in all of the tumors, there was colocalization with the cancer epithelial cells and immunosuppressive macrophages. Within proximity were the T cells (specifically the CD4 T cells), and we were able to elucidate specific pathway enrichment in both the macrophages and the T cells. We show that these macrophages have enrichment of classical anti-inflammatory pathways, such as those involving the upregulation of scavenger receptors, IL-4, and IL-13, and that this decreases antigen processing and anti-tumor effects of the associated T cells. **This new data can be found in Fig. 7, panels G through I**.

Additionally, we also re-analyzed our rat snRNA-seq data and performed a deeper analysis on the macrophages and T cells. Our findings corroborate what we observe in humans – macrophages from tumors in older rats have increased sensitivity to chemokines and have a decreased ability to promote T cell differentiation and activation. Similarly, T cells from tumors in older rats show decreased enrichment in a number of anti-tumor pathways. We have also added this data to the manuscript – it can be found in **Fig. 2G**.

[R1-5] From a statistical perspective, the manuscript is sound, though some improvements could be made. Greater clarity on how uncertainties in inflammatory marker measurements were addressed would strengthen confidence in the findings. It would also be helpful to explain how sample sizes were determined for different experimental groups and to consider potential confounding factors, such as metabolic differences in aged rats or variations in hormone replacement therapy among human participants.

We appreciate the comment and are happy to provide additional clarification.

First, with respect to uncertainty in inflammatory marker measurements, all circulating and tissue inflammatory factors were quantified using validated multiplex immunoassays and were analyzed using standardized preprocessing, normalization, and quality control procedures. With respect to chemokine identification, these factors were not selected a priori. Instead, they emerged from unbiased regression-based analyses across multiplex inflammatory panels, with age as the primary predictor. The same chemokines were independently identified in aged rats, healthy donors, and patients with ER+/HER2- breast cancer and were subsequently shown to have functional consequences in macrophage polarization assays, supporting their biological relevance.

Sample sizes for human specimens were determined by the availability of matched plasma and tissue samples within our institutional biospecimen repositories and the Komen Tissue Bank, reflecting the largest cohorts with such matched specimens available to us. Given the observational nature of these cohorts, power calculations were not performed. Sample size determination and inclusion criteria are specified in the Study Design section in the Methods.

Finally, we carefully considered potential confounding factors. In the rat studies, although metabolic parameters were not directly measured, the absence of age-associated increases in tumor mutational burden or oncogenic pathway activation argues against systemic metabolic acceleration as the primary driver of the observed phenotypes. In the human cohorts, prior hormone replacement therapy use was recorded and is reported (**Supplementary Table 2**); importantly, no participants were receiving hormone replacement therapy at the time of specimen collection. While residual confounding cannot be fully excluded in cross-sectional human studies, the convergence of systemic, local, and functional data supports an aging-associated inflammatory and hormonal phenotype that is not readily explained by these factors alone.

[R1-6] The conclusions drawn from the study are largely well-supported but would benefit from additional mechanistic validation. In particular, the study should more explicitly differentiate correlation from causation when discussing inflammatory markers and tumor development. Further in vivo experiments testing whether

interventions targeting chronic inflammation can alter tumor progression would enhance the manuscript's translational relevance.

We appreciate the reviewer's guidance on distinguishing correlation from causation and on translational relevance. We have revised the discussion (with a specific limitations section in lines 663-675) to more explicitly delineate these boundaries. Specifically, associations between circulating inflammatory markers and age are described as correlational, whereas mechanistic inference is restricted to the local tumor microenvironment, where functional experiments demonstrate that chemokines and E2 are sufficient to drive immunosuppressive macrophage polarization.

As we describe above, while we did not perform in vivo pharmacologic modulation of inflammation, we show that targeted intervention at the tissue level using chemokine blockade and fulvestrant reverses macrophage polarization and alters downstream cytokine secretion in human-derived organoids. We do agree that in vivo targeting of chronic inflammation represents an important future direction but is perhaps outside of the scope of this work.

[R1-7] To build upon the existing findings, the authors should consider:
- Conducting **in vivo inhibition studies** on HSD17B7 to confirm its role in tumor estrogen metabolism.
- Measuring Eotaxin (CCL11) levels, as it has been implicated in aging-related immune dysregulation and could provide a more complete picture of inflammatory changes.
- Performing functional assays on T cells, particularly cytokine secretion profiling, to determine whether immune suppression extends beyond macrophage activity.
- Expanding on CCL2 blockade experiments, considering its potential as a therapeutic target.

Again, we appreciate the reviewer's detailed review of the manuscript and these suggestions.

Regarding Eotaxin, we appreciate that this is an important factor. However, since we have already analyzed all of the plasma samples included in the study, we are not able to go back and analyze them again for Eotaxin.

While we did not perform in vivo inhibition studies with the HSD17B7 inhibitor (as noted above this would take 1-2 years with the carcinogen-induced tumors and there are several other experimental caveats), but we did perform in vitro pharmacological inhibition studies with the PDOs from older patients (as described above).

As detailed above, we also completed spatial sequencing on a new cohort of older patients with ER+/HER2- breast cancer, which specifically focuses on the detailed interactions between cancer cells, macrophages, and T cells. While functional assays on the T cells are out of the scope of this work given the focus on aged macrophages, we did include T cell-directed analyses in the spatial sequencing data and in our mIHC data (all detailed in **Fig. 8G, 8H, and 8I**). We have now also performed a deeper analysis on the T cells in the tumors from older rats (**new data in Fig. 2G**).

We appreciate the reviewer's suggestion to further expand on the CCL2 axis, given its therapeutic potential. In the current manuscript, we demonstrate that CCL2: (i) emerges from unbiased regression-based analyses as one of the most consistently age-associated inflammatory mediators across species and cohorts; (ii) is enriched in the aged tumor microenvironment; (iii) is sufficient, in combination with estradiol, to promote immunosuppressive macrophage polarization in vitro; and (iv) is partially reversible through pharmacologic CCL2 blockade. Importantly, combination treatment with CCL2 blockade and estrogen receptor antagonism produced a greater restoration of anti-tumor cytokine balance than either intervention alone, supporting cooperative inflammatory and hormonal signaling in the aged tumor microenvironment. We believe these data provide mechanistic and translational support for the CCL2 axis as a therapeutic target in ER+ breast cancer in older patients. While additional in vivo interrogation of CCL2-directed strategies would be valuable, such studies are beyond the scope of the present manuscript.

[R1-8] The manuscript does a good job of referencing prior work, but Sayed et al., Nature Medicine 2019

should be included to provide a stronger theoretical grounding for the discussion on chronic inflammation. Proper credit to previous research will help situate the study more effectively within the existing literature.

We edited both the introduction and discussion to provide a stronger theoretical foundation for the use of chronic inflammation in this paper. Of note, Sayed et al Nat Med 2019 is not the correct citation – we believe the reviewer meant Furman et al Nat Med 2019, which we already cited. Sayed et al Nat Aging – the paper that describes the iAge clock – is also cited given its findings of circulating CXCL9 as an age-related marker of inflammation.

[R1-9] Overall, the manuscript is well-written and presents an important contribution to the field. The abstract should more explicitly highlight the novel aspects of the study, and the introduction could do more to frame the findings in the context of past research. The conclusion should emphasize the translational implications of the work, particularly in terms of how targeting inflammation and estrogen metabolism could inform new therapeutic approaches. This study provides valuable insights into the intersection of inflammation, estrogen metabolism, and immune suppression in ER+ breast cancer. However, refining the conceptual framework around chronic inflammation, expanding experimental validation, and incorporating additional inflammatory markers like Eotaxin (CCL11) would further strengthen its impact. Addressing these points would enhance the robustness and applicability of the findings, ensuring that they contribute meaningfully to the broader understanding of cancer and aging.

Thanks to the reviewer for their thorough review. In response, we have revised the abstract, introduction, and conclusion to more clearly emphasize the novel conceptual and translational aspects of this work. As noted above, we strengthened the chronic inflammation conceptual framework to more clearly position our findings within the context of prior aging and cancer research, explicitly defining chronic inflammation as a heterogeneous, chemokine-biased process in aging and distinguishing association from mechanism. We have also strengthened the abstract and conclusion to underscore the translational implications of our findings, particularly the potential for targeting local estrogen conversion and chemokine-driven inflammation as age-informed therapeutic strategies. We believe these revisions substantially strengthen the manuscript and clarify its contribution to understanding how aging-associated systemic factors shape tumor biology and immune function.

Reviewer #2:

Carleton et al. present solid data providing molecular explanations for the development of estrogen receptor alpha (ER)-positive breast cancer in older women. The vast majority of breast tumors that developed in the elderly are ER+ despite low circulating levels of estrogens. The authors show that HSD17B7 in tumors enables local conversion of estrone to 17b-estradiol as a potent estrogen. In addition, a chronically inflamed and immunologically compromised tumor immune microenvironment help drive tumor growth. Pharmacologic targeting of HSB17B7 decreased local E2 levels and prevented macrophage polarization towards an immunosuppressive phenotype in vitro. Preclinical findings in an aged rat model of ER+ BC were validated with correlative analysis of human tissue specimens. A major concern is the lack of animal experiments testing whether HSB17B7 inhibition/loss affects mammary tumor phenotypes. The following issues should also be addressed:

We appreciate this concern, which is shared by Reviewer 1. Please see our response to R1-3 above. In short, while in vivo HSD17B7 inhibition was thought to be a significant undertaking not possible in the time frame of a resubmission (it takes 1-2 year to develop and analyze carcinogen-induced tumors), we were able to perform additional pharmacological inhibition studies in our prospectively collected human PDOs from older patients. These experiments, with all the new data in **Figure 5 and Extended Data Fig. 8**, greatly strengthened our conclusions about HSD17B7 inhibition.

[R2-1] Lines 157-8, it is stated about rats that “Four months of age corresponds to about the age of 20-30 years for humans while 21-23 months of age is the equivalent to about the age of 70 years for humans (Fig. 1A).” This conclusion and figure should be supported by data, such as DNA methylation to determine chronological age. If data are unavailable, it should be explained how (i.e., the basis by which) rats at the ages studied are thought to reflect the human condition.

Such methylation data are not available; however, we derived this based on the survivorship curves, equating the percentage living across the two species. We have edited the figure legend of **Figure 1** to provide additional details on how the rat age was thought to reflect the human condition: “Survivorship curve showing the human equivalent age for the younger and older rats used in the study, which is based on general human population survivorship curves and F344 rat survivorship curves from the National Institute on Aging (NIA).”

[R2-2] Fig. 1C suggests that only a minority of young rats formed tumors, but such tumors grew relatively fast in Fig. 1E. Did young rats develop more carcinomas and fibroepithelial lesions? It would be helpful to include more information on young rats given that it seems only 3 tumors were used in Fig. 1E-G.

The reviewer is correct – only three rats developed tumors, and all were sequenced and included throughout **Figure 1 and 2**. Given all of the downstream analyses we performed, we speculatively thought the reason behind this was the intact immune system in younger rats that prevented the outgrowth of additional lesions. No young rats developed fibroepithelial lesions. Fibroepithelial lesions are known to spontaneously form in aged F344 rats – however, we also anecdotally believe that the fibroepithelial lesions we observed were accelerated by the carcinogen.

We have included more information in both the manuscript main text (lines 189-193) and in the figure legend of **Figure 1** to address this comment.

[R2-3] In Fig. 1F-G, please indicate in legend the n of tumors examined, and the n of HPF analyzed. It is currently unclear whether the graphed data points represent tumors or fields.

We have edited the legend in **Fig. 1F and 1G** (lines 1449-1451) to address this comment.

[R2-4] Fig. 3E shows a Composite Circulating Chemokine Score for each patient/donor. The meaning of this Score is unclear because it assigns equal weight to each chemokine, and resultant data are binned (obscuring

the linear distributions of chemokines shown in Fig. 3D). Please justify the use of this scoring algorithm or consider removing these data from the manuscript.

Thanks for this comment – we can provide additional information on this scoring algorithm. Calculation of the composite circulating chemokine score was done to determine the collective burden of circulating CCL2, CCL3, CCL4, and CXCL9. It was calculated as the number of markers two standard deviations above the marker mean for each patient. This is a common methodology in immunology to address when certain factors are changing together. We have added details to the methods (lines 925-927) and in the legend of Figure 3.

[R2-5] Fig. 4D compared gene expression levels across age groups in 3 cohorts. It needs to be clarified whether this analysis was limited to patients with ER+ (HER2-negative?) disease, as the ER+ subpopulation is most relevant to this study and should at least be presented in suppl. data. Curiously, tumor ESR1 levels increase with age, and this should be mentioned; is this caused by a preponderance of ER+ disease in older patients when all cancer subtypes are analyzed together? The data are shown as a heatmap, but should also be presented (in suppl.) as individual data points for each tumor with relevant statistical comparisons; it is not obvious from the heatmap why HSD17B7 was selected for further study. Fig. 4E shows violin plots with dots (for outliers?). It looks like the bulk of data are in the compressed violins. Please adjust this graph to better illustrate the difference in levels between groups.

We thank the reviewer for this important point and apologize for the lack of clarity. All human tumor analyses presented in this manuscript, including those shown in **Fig. 4D** and **Fig. 4E**, were restricted exclusively to ER+/HER2- breast cancers. This includes tumors from the TCGA, METABRIC, and SCAN-B datasets, as well as all scRNA-seq analyses, mIHC analysis, and spatial transcriptomic analysis. We have now explicitly stated throughout the manuscript that tumors analyzed were ER+/HER2-.

Regarding *ESR1*, we did mention this in the text, and **Extended Data Figure 6** has more data from TCGA (mRNA expression, methylation of ESR1, and proteomics of ER) and from METABRIC and SCAN-B about this. All of this analysis only shows data from ER+/HER2- breast cancer as well. The increase in *ESR1* with age is a known phenomenon and has reported elsewhere recently (Parsons et al, 2025, Nature Aging). We left Figure 4D as is given that it shows the panel of genes of interest. However, we do also have an extended data figure (**Extended Data Figure 4**) that individually analyzes all the hydroxysteroid enzymes involved in E1 and E2 biosynthesis. We believe that the combination of the heatmap in **Fig. 4D**, the individual values in **Extended Data Figure 4**, and the violin plot in Fig. 4E all justify further study of HSD17B7.

With respect to the violin plots shown in **Fig. 4E**, these data are derived from single-cell RNA sequencing, where gene expression is inherently sparse and zero-inflated. As a result, violin plots are commonly used to display the full distribution of expression values across thousands of individual cells, including the large proportion of cells with zero detectable expression. In this context, the apparent compression of the violins reflects the underlying biology of cell-type restricted expression rather than data truncation or outlier-driven effects. Importantly, HSD17B7 expression is restricted to a subset of cancer epithelial cells, and the observed differences across age groups arise from shifts in the fraction of expressing cells and their expression levels, both of which are appropriately captured by this visualization. For clarity, we have ensured that the figure legend explicitly notes that the violins represent single-cell expression values, and that zero-expression cells are included in the distributions.

[R2-6] Line 284- please change to "… panel of genes encoding enzymes…."

Thank you for catching this – we made this edit.

[R2-7] Figs. 4G and 6I are cut off on right and left sides. In Fig. 4G, ER IHC score ranges from 150-300, implying that all tumors were ER+ here; is this correct?

It appears that the PDF cut off some of the axes. Yes, in **Fig. 4G**, all tumors analyzed were ER+/HER2-. The maximum H-score value is 300. We have edited these figures to ensure that the sides are clearly visible.

[R2-8] Line 345- the associated data seem to be in Extended Data Fig. 9 (not S11).

We apologize for this error – we have ensured that the Extended Data and Supplementary figures are correctly labeled throughout the manuscript.

[R2-9] Fig. 5B- to emphasize results, consider moving the "Chemokine Tissue Inflammation" row to top of figure, use a different pair of colors for that row, and add a scale bar for the heatmap.

Thank you for this suggestion. We have moved Chemokine Tissue Inflammation to the top row and added a scale bar to the heatmap. We have rendered a new heatmap, which hopefully makes the chemokine row stand out more.

[R2-10] For Fig. 6G, please include H&E or IHC images depicting "tumor and macrophage enriched neighborhoods."

We have included the mIHC images in **Extended Data Figure 10**. The specific neighborhoods of interest are indicated with a white box.

Reviewer #3:

This is a well written manuscript that stories a beautiful translational story deciphering the relationship between E2, inflammation and age. The conclusions are translationally very relevant. Minot comments below:

[R3-1] Spilt age groups omitted 45-55yo, this is a key patient population that is perimenopausal. Can you explain why omitted?

Thanks for this comment. We intentionally omitted the perimenopausal group. Estrogen levels change variably across these ages, and we wanted to have stronger young vs. middle-age vs. old comparison without potential confounding from the peri-menopausal group. We have further clarified this in the manuscript in lines 786-788.

[R3-2] No differences in tumor grade across age groups many studies suggesting that the incidence of aggressive breast cancers is higher in younger patients, why different?

We appreciate this comment. This may just be a limitation of our sample size across the age groups, as the data we show in **Supplementary Table 1** shows a clear enrichment of grade I and II tumors in the older group (though not statistically different).

[R3-3] How do you explain higher peripheral E2 levels in postMP ER+ pts, vs donors, who are presumably on AI therapy? If data is available regarding AI use this could help understand this observation.

First, we should note that all patients with ER+/HER2- breast cancer included in the study were untreated at the time of surgery and blood collection (i.e., no patients received neoadjuvant AI therapy). We do, however, agree with the reviewer that this is an intriguing piece of data that we have been trying to better understand. We don't have a suitable explanation for this. Because this blood was taken at the time of surgery, we do not know if these patients had higher circulating E2 levels are we thus more likely to develop breast cancer, or if this increase in E2 occurred after tumor formation (perhaps a spillover effect from the breast TME?). This is an excellent point that we have added to the discussion (lines 589-593).

[R3-4] Figure 3E shows increasing inflammatory cytokine score with age in ER+ breast cancer, was this also the case in healthy donors? Ie is this a differential observation according to breast cancer status vs an association with age?

Yes, this did also occur in the donors. We think this is more of an association with age rather than a function of breast cancer status – if you look at the data in **Fig. 3D**, most of these markers increase in donors in addition to in patients with breast cancer.

[R3-5] Interesting observations between BMI and E2 in TME; if data on breast density is available this could be further evaluated as this is a known risk factor for breast cancer, and may offer a radiologically quantifiable biomarker.

Unfortunately, we do not have breast density data at this time and are thus not able to provide further data on this comment, though we do appreciate the role of breast density as a risk factor.

26 May 2026

NATAGING-A09190B – Systemic and local chronic inflammation and hormone disposition promote a tumor-permissive environment for breast cancer in older women

Response to Editor & Reviewers

Dear Dr. Thuault & Dr. Walters,

We thank you and the reviewers for a thorough evaluation of our revised submission and are thrilled to see the manuscript accepted in principle.

We have addressed all of the remaining edits from Reviewer 1 and detailed our response to the reviewer below in red.

We thank you again for considering our revised manuscript. We look forward to hearing from you soon.

Sincerely,

Adrian Lee, PhD
Steffi Oesterreich, PhD
Neil Carleton, MD, PhD

Reviewer #1 (Remarks to the Author):

The authors present a revised version of their manuscript examining how aging associated changes in systemic inflammation and hormone metabolism shape the tumor microenvironment in breast cancer. The study integrates an aged rat model, human biospecimens, large transcriptomic datasets, and patient-derived organoids , and proposes that a chemokine-enriched inflammatory state and local estrogen conversion (via HSD17B7) together promote an immunosuppressive, tumor-permissive environment in older patients.

The revised manuscript is substantially improved- In particular, the authors have clarified the conceptual framework around chronic inflammation, better distinguished correlation from mechanism, and added meaningful functional data (notably organoids-based inhibition studies and expanded macrophage analyses). These additions strengthen the overall narrative and enhance the translational relevance of the work.

Despite these improvements, several important issues remain, primarily related to the strength of mechanistic conclusions relative to the available data.

We thank you again for your thorough evaluation of our manuscript! We sincerely appreciate your effort in making the manuscript stronger.

Major Comments

1. The revised manuscript more clearly distinguishes association from mechanism, which is appreciated. However, the central model—linking aging, systemic inflammation, local estrogen conversion, and immune suppression—still relies largely on associative human and animal data, and tissue-level functional experiments (organoids and macrophage assays).

There remains no direct in vivo evidence demonstrating that HSD17B7 is required for tumor development or progression in aged systems, or chemokine signaling (eg, CCL2) causally drives macrophage-mediated immune suppression in vivo. While the authors appropriately acknowledge these limitations, some statements in the manuscript still imply a level of causality and translational readiness that is not fully supported.

Please further temper causal and therapeutic claims throughout, and consistently distinguish between association, ex vivo mechanism, and untested in vivo causality.

We have reviewed the entirety of the manuscript and have further tempered causal claims and language throughout. We have also, where appropriate, indicated the whether the conclusion is based on in vitro, ex vivo, or in vivo data. Changes to the abstract, the end of the introduction, and the results were all made to address this comment.

2. The authors provide a clearer justification for the use of the F34 rat model, which is helpful. However as acknowledged-- this model does not fully recapitulate human postmenopausal estrogen biology, particularly the shift toward estrone predominance. Given that the HSD17B7 hypothesis depends on E1-to-E2 conversion, this limitation continues to weaken the integration between the animal model and the human findings.
More explicitly separate conclusions derived from the rat model (aging and immune context) from those derived from human and PDO analyses (estrogen metabolism).

Thanks for this comment. In response, we have added text to lines 239-244 at the end of the first results section that presents the rat data to better explain the strengths and limitations of the rat model.

3. The addition of organoid-based inhibition experiments is a clear strength of the revision and provides important support for the proposed role of HSD17B7. However, validation remains limited to pharmacologic inhibition, no genetic perturbation (e.g knockdown/CRISPR) is included and some effects in E2-treated conditions raise questions about specificity. Thus, HSD17B7 is well-supported as a candidate target but not yet

fully validated as a therapeutic mechanism. Please moderate claims accordingly and clearly frame HSD17B7 as a promising but not yet definitive target.

Thanks for this comment. We have now edited the results (especially the text that describes Figures 4 and 5) as well as the discussion (lines 621-625) to moderate claims about the promise (yet not definitive) target of HSD17B7. We have also made a number of small wording edits throughout where appropriate to moderate language around this target.

4. The revised manuscript includes stronger evidence for immunosuppressive macrophage states, including spatial analyses and improved pathway characterization. However, the current framework still simplifies macrophage biology, which is highly heterogeneous. In addition, as far as I can tell, functional consequences for T cells remain largely inferred and the relative importance of specific chemokines is not fully resolved. Expand discussion of macrophage heterogeneity and avoid overgeneralizing polarization states.

Thanks again for this comment. This makes sense and we agree. We did try to avoid using the M1/M2 nomenclature and tried to stick with descriptions of macrophages based on their markers (i.e., CD206+). In Supplementary Figure 5G, we did try to identify if there was a predominant macrophage subtype in the aged tumors from the scRNA-seq data – yet the sample size was low and we did not identify a clear pattern.

To address this comment, we added a section of text to the discussion (lines 654-662) discussing macrophage heterogeneity as well as text the limitations paragraph in the discussion (lines 693-695) discussing the inferred T cell data.

Minor Comments

1. Provide clearer explanation of analytical approaches (for instance, MICA) for broader readership.

We have added text to the methods to clarify use of the MICA approach (lines 998-1002) that would be understandable to broader readership.

2. Ensure all sample sizes are explicitly stated in figures and legends.

We have checked the figures and legends throughout to explicitly state sample sizes.

3. Clarify inclusion criteria and handling of missing data in human cohorts.

We have added text the methods (lines 727-731) to clarify specimen inclusion criteria and handling of missing data.

4. Consider adding a schematic distinguishing associative findings, functional (ex vivo) evidence and untested causal mechanisms.

We have edited our schematic in Figure 8J to dampen causal language.

Acceptable after further revision focused on refining interpretation and appropriately calibrating mechanistic and translational claims.

Thanks again for your review of our manuscript.

Reviewer #2 (Remarks to the Author):

The authors have satisfactorily addressed the prior critiques.

Reviewer #3 (Remarks to the Author):

Thank you for addressing my comments, no further concerns.
